# Localized expression of the *Dwarf14-like2a* gene in rice roots on infection of arbuscular mycorrhizal fungus and hydrolysis of *rac*-GR24 by the encoded protein

**DOI:** 10.1080/15592324.2021.2009998

**Published:** 2021-12-14

**Authors:** Thongkhoun Sisaphaithong, Megumi Yanase, Tsubasa Mano, Shigeru Tanabe, Eiichi Minami, Aiko Tanaka, Shingo Hata, Yoshihiro Kobae

**Affiliations:** a Graduate School of Bioagricultural Sciences, Nagoya University, Nagoya, Japan; b School of Agricultural Sciences, Nagoya University, Nagoya, Japan; c National Institute of Agrobiological Sciences, Tsukuba, Japan; d Ryukoku University, Seta Oe-cho, Otsu, Japan

**Keywords:** D14-like2, *in vitro* hydrolysis, *Oryza sativa*, *Rhizophagus irregularis*, strigolactone, symbiosis

## Abstract

Strigolactones (SLs) are plant hormones that control diverse aspects of the shoot and root growth and are exuded into the soil as recruitment signals for arbuscular mycorrhizal (AM) fungi. SL signaling in plants is transduced *via* the α/β-hydrolase receptor Dwarf14 (D14). The D14 family consists of D14, Dwarf14-like (D14L), and Dwarf14-like 2 (D14L2) clades in rice. The D14L receptor is known to condition pre-symbiotic perception of AM fungi. In this study, it was found that the *Dwarf14-like2a* (*D14L2a*) gene expression was significantly induced by AM fungal colonization. The transcript of *D14L2a* appeared not only in mature arbuscule-containing cells but also in epidermal/cortical cells at an early colonization stage and near the elongating intercellular hyphae. *D14L2a* transcript was detected normally in mycorrhizal roots of *str1-2* mutant that form stunted arbuscules, suggesting that the gene expression is independent of arbuscule development. Moreover, the recombinant D14L2a protein exhibited hydrolase activity of synthetic SL, *rac*-GR24. Based on these results, we discussed the role of D14L2 in the establishment of AM symbiosis.

## Introduction

Arbuscular mycorrhizal (AM) fungi of the subphylum, Glomeromycotina, establish symbiotic associations with most land plant species. In exchange for photosynthates from the host plants, fungi transport phosphate and other mineral nutrients from the soil *via* extraradical and intraradical hyphae to plant roots.^1–3^ Typically, fungal intraradical hyphae form highly branched structures called arbuscules within the root cortical cells of the host plants. Fungal arbuscules are enveloped by plant plasma membrane-connected periarbuscular membranes, which are the primary site of nutrient exchange between the two partners.^1–3^ At the beginning of the AM symbiosis, the exchange of chemical compounds occurs for partner recognition. The roots of host plants exude strigolactones (SLs), a class of carotenoid-derived plant hormones,^4^ that activate the hyphal branching of AM fungi and respiratory metabolism.^5,6^ In return, AM fungi secrete lipochitooligosaccharides or short-chain chitooligosaccharides; the reception triggers transcriptomic remodeling in the plants, which eventually leads to fungal colonization.^7–9^ Although the biosynthesis of SLs was reported to be elevated under AM symbiosis, and SLs were necessary for efficient hyphopodium formation of AM fungi,^10,11^ the roles of SLs, other than acting as recruitment signals for AM fungi, are unclear.

In rice (*Oryza sativa* L.), SL signal transduction occurs *via* the α/β-hydrolase receptor Dwarf14 (D14). Rice D14 and its orthologs in other plants not only transduce SL signals but also hydrolyze SL molecules.^4,12^ Although there have been contradictory reports on the timing and necessity of the hydrolysis, D14 receives SL and forms a complex with F-box protein D3 and the negative regulator of SL signaling D53, which results in ubiquitination and rapid degradation of D53 through the 26S proteasome pathway.^13–16^ Finally, the degradation of D53 allows the expression of downstream genes.^17^

The D14 family consists of three clades, namely, D14, Dwarf14-like (D14L/KAI2/HTL), and Dwarf14-like 2 (D14L2/DLK2).^18–20^ Among them, D14L is evolutionarily the oldest^18,21^ and necessary for the pre-symbiotic perception of AM fungi.^10,22^ Although its *Arabidopsis* homolog, AtKAI2, was reported to bind smoke-derived karrikins,^23^ it is currently unclear whether rice D14L receives signals from AM fungi or endogenous ligands of plants.^24^ Anyway, D14L triggers the degradation of a rice ortholog of *Arabidopsis* Suppressor of MAX2-1 (SMAX1),^25^ allowing the expression of downstream genes for both AM symbiosis and SL biosynthesis.^10^ The signal transduction pathway of D14L2 is unknown.^19^ Also, it has long been unclear whether or not D14L2 is involved in the process of AM colonization. Very recently, however, Ho-Plágaro et al. revealed that a tomato ortholog of *D14L2* (*SlDLK2*) negatively regulates arbuscular hyphal branching *via* knockdown and overexpression experiments.^20^

In this study, we performed transcriptome analysis of rice AM roots using the microarray approach and found that the *Dwarf14-like2a* (*D14L2a*) gene expression was significantly induced by the colonization of an AM fungus. In order to gain insights into the role of *D14L2a* in the establishment of AM symbiosis, we examined timing and location of the *D14L2a* gene expression. To characterize the D14L2a protein, we also examined its hydrolase activity.

## Materials and methods

### Plant growth, fungal material, and inoculation

Rice seeds (*Oryza sativa* cv. Nipponbare) were immersed in deionized water containing 1% (w/v) Benrate (Sumitomo Chemicals, Tokyo, Japan) for 3 d. The germinated seeds were rinsed with deionized water three times and then transplanted individually into 40-mL cells in a plastic tray containing an autoclaved Kanuma soil/vermiculite/Kureha soil (Kureha, Tokyo, Japan) mixture (1:1:1, by volume) with a modified Hoagland solution the phosphate concentration of which was reduced to 0.1 mM. Each mycorrhizal plant was inoculated with approximately 1000 spores of *Rhizophagus irregularis* DAOM (197198) (Premier Tech, Riviere-du-Loup, Canada) and then grown in a greenhouse with a 16-h d/8-h night cycle at 27°C for 25 d. Water was supplied from the bottom by maintaining a water level of up to 5 mm. Control non-mycorrhizal plants were also grown in parallel without inoculation of the fungus. The mycorrhizal seedlings of *str1-2* mutant (a kind gift from Dr. Caroline Gutjahr at the University of Munich and Dr. Uta Paszkowski at the University of Cambridge) were prepared in a similar manner.^26^

### Extraction of total RNA, microarray analysis, and reverse-transcription (RT)-PCR

Total RNA was extracted from three biological replicates of mycorrhizal or non-mycorrhizal roots using a plant RNA extraction kit (Viogene, Sunnyvale, CA). Labeling of cRNA with cyanine-3 (Cy3), fragmentation, and hybridization of the cRNA with a slide of rice 4 × 44 K microarray RAP-DB (G2519F#15241; Agilent Technologies) were conducted as described by Takehisa et al.^27^ Washing and scanning of the hybridized slide were also as performed.^27^ For semi-quantitative RT-PCR, the total RNA samples were reverse-transcribed using an oligo(dT) primer and ReverTra Ace (Toyobo, Osaka, Japan), and then subjected to PCR. PCR was performed using KOD-FX DNA polymerase (Toyobo), and the PCR products were visualized after agarose gel electrophoresis. The specific primer sequences are presented in Supplementary Table S1.

### Expression of the D14L2a promoter-β-glucuronidase (GUS) reporter

A genomic fragment of *D14L2a* (Os05g0590300/LOC_Os05g51240) containing a promoter region (2,280 bp in size) and a short coding region (33 bp in size) was amplified from *O. sativa* (cv. Nipponbare) *via* PCR using a primer pair *D14L2a* promoter_F and R. The amplified gene was ligated into an entry vector, pENTR/D-TOPO (Invitrogen, Waltham, MA). It was then introduced upstream of the promoterless GUS gene with a nopaline synthase terminator in a binary vector, pGWB203, using the Gateway system (Invitrogen). The resulting construct encodes a fusion protein composed of 11 amino acids from the N-terminus of D14L2, extra 25 amino acids, and entire GUS. An *OsPT11* (Os01g0657100/LOC_Os01g46860) promoter-GUS construct containing 2,659-bp promoter region was prepared in a similar manner. The resulting construct encodes a fusion protein composed of 15 amino acids from the N-terminus of OsPT11 and entire GUS. Finally, the binary plasmid constructs of pGWB203 were introduced into *Agrobacterium* (*Rhizobium radiobacter* EHA105). Transformation of rice (cv. Nipponbare) was performed as reported by Toki et al.,^28^ and mycorrhizal seedlings of the transformants were prepared as described above. The transformed roots were cut into 2–5 cm in length; stained with 100-mM phosphate buffer (pH 7.0) containing 2-mM 5-bromo-4-chloro-3-indolyl-β-D-glucuronide, 0.5-mM potassium ferricyanide, and 0.5-mM potassium ferrocyanide for around 3 h; and then observed *via* microscopy. Next, the root samples were incubated in 50% ethanol, cleared with 20% KOH, neutralized with 0.1 M HCl, rinsed with distilled water, and then stained with 0.2-µg/mL wheat germ agglutinin-Alexa Fluor 488 conjugate (Invitrogen) in phosphate-buffered saline. They were observed using a fluorescence microscope (Nikon, Tokyo, Japan).

## *Detection of* rac*-GR24 hydrolysis in vitro*

The coding region of *D14L2a* was amplified *via* RT-PCR from the cDNA of mycorrhizal rice roots using the *D14L2a* coding region_F and R primer. It was inserted into a cold-shock expression vector pCold II (Takara Bio, Otsu, Japan) with the aid of the *Eco* RI and *Pst* I sites included in the primers. The resulting recombinant protein consists of N-terminal extra 22 amino acids containing a His-tag and the entire D14L2a. To prepare the coding region of *DAD2*,^12^ total RNA was extracted from the leaves of commercial dwarf petunia (*Petunia hybrida*), cDNA was synthesized, and RT-PCR was performed as described above using the *DAD2*-coding region_F and R primer. It was inserted into pCold II using *Xho* I and *Hin*d III sites included in the primers. The resulting protein consists of N-terminal extra 18 amino acids containing a His-tag and the entire DAD2. The pCold II constructs were introduced into *Escherichia coli* BL21. The *E. coli* cells were grown at 37°C in LB containing 50-µg/mL ampicillin until OD_600_ = 0.5 and then incubated at 15°C for 24 h in the presence of isopropyl β-D-thiogalactopyranoside to express the recombinant proteins under controls of *cspA* promoter and *lac* operator. The concentrations of the β-galactosidase inducer were 1 and 0.1 mM for D14L2a and DAD2, respectively. The recombinant proteins were extracted from the *E. coli* cells using xTractor Buffer (Takara Bio), bound to TALON spin columns (Takara Bio), washed once without imidazole, washed several times with 20-mM imidazole, and then eluted with 150-mM imidazole. The removal of imidazole and concentration of the recombinant proteins were conducted using Vivaspin 500–10 K columns (GE Healthcare Japan, Tokyo, Japan). Synthetic *rac*-GR24 (a kind gift from Dr. Kohki Akiyama at Osaka Prefecture University) is a nearly equimolar mixture of GR24^5DS^ and GR24*^ent^*^−5DS^.^4,29^ Hydrolysis of 1-mM *rac*-GR24 was performed at 25°C for 18 h as described in the supplemental information of Hamiaux et al.,^12^ except that 73-µM (instead of 50-µM) recombinant proteins were added to the reaction mixtures. Subsequently, thin-layer chromatography was performed as described by Hamiaux et al.^12^

## Results

### Induction of D14L2a gene in AM roots

As the initial experiment, we investigated transcriptomic alterations of rice roots associated with colonization of AM fungus. A portion of the results of the microarray experiments is presented in Table 1 (for the full results, see Supplementary Table S2). Among the genes involved in SL biosynthesis, *D17* that encodes carotenoid cleavage dioxygenase CCD7 was highly induced, and the expression of other genes was increased by the colonization of the AM fungus. Among the SL signaling genes and SL-related putative signaling genes presented in Table 1, *D14L2a* exhibited the highest induction rate by the colonization of the AM fungus. Although the expression of *D14L2b*, a close paralog of *D14L2a*, was also elevated, hereafter, we will focus our attention to *D14L2a*. Other genes involved in SL signaling demonstrated modest changes. The microarray experiments also showed that *OsPT11* (Os01g0657100/LOC_Os01g46860), a functional marker of arbuscules,^30,31^ was highly induced by AM symbiosis (461–1240 fold; Supplementary Table S2). *OsSTR1* (Os09g0401100/LOC_Os09g23640) encodes a half-size ABC transporter and is thought to be involved in lipid supply from host plants to AM fungi.^26,32–34^ This gene was also highly induced by AM colonization (802-fold; Supplementary Table S2).

**Table 1.** Expression of genes involved in SL biosynthesis, SL signaling, and SL-related putative signaling

Gene				
(Synthesis)	RAP ID	Myc- ^1^	Myc+ ^1^	Myc+/Myc-
D27	Os11g0587000	− ^2^	− ^2^	− ^2^
D17	Os04g0550600	576 + 43	6633 + 1076	11.5 *
D10	Os01g0566500	75 + 18	139 + 36	1.8 ns
MAX1 homolog	Os01g0700900	2545 + 428	9184 + 1890	3.6 *
MAX1 homolog	Os01g0701400	584 + 122	1874 + 358	3.2 *
(Signaling)				
D14	Os03g0203200	3085 + 110	2953 + 58	1.0 ns
D14L	Os03g0437600	57 + 4	68 + 24	1.2 ns
D14L2a	Os05g0590300	310 + 77	16874 + 3214	54.4 *
D14L2b	Os01g0595600	238 + 2	5610 + 459	23.6 **
D3	Os06g0154200	193 + 16	196 + 9	1.0 ns
D53	Os11g0104300	52 + 10	50 + 16	1.0 ns
SMAX1	Os08g0250900	963 + 18	2025 + 83	2.1 **

^1^Fluorescence of Cy3. Mean + SD (n = 3). Arbitrary unit. Myc-, non-mycorrhizal roots; and Myc+, mycorrhizal roots.

^2^This gene (Os11g0587000/LOC_Os11g37650) was not included in the microarray.

** *P* < 0.01; * 0.01 < *P* < 0.05; ns, no significant difference, Welch’s t test (Myc- versus Myc+).

### Semi-quantitative RT-PCR

**Figure 1.**
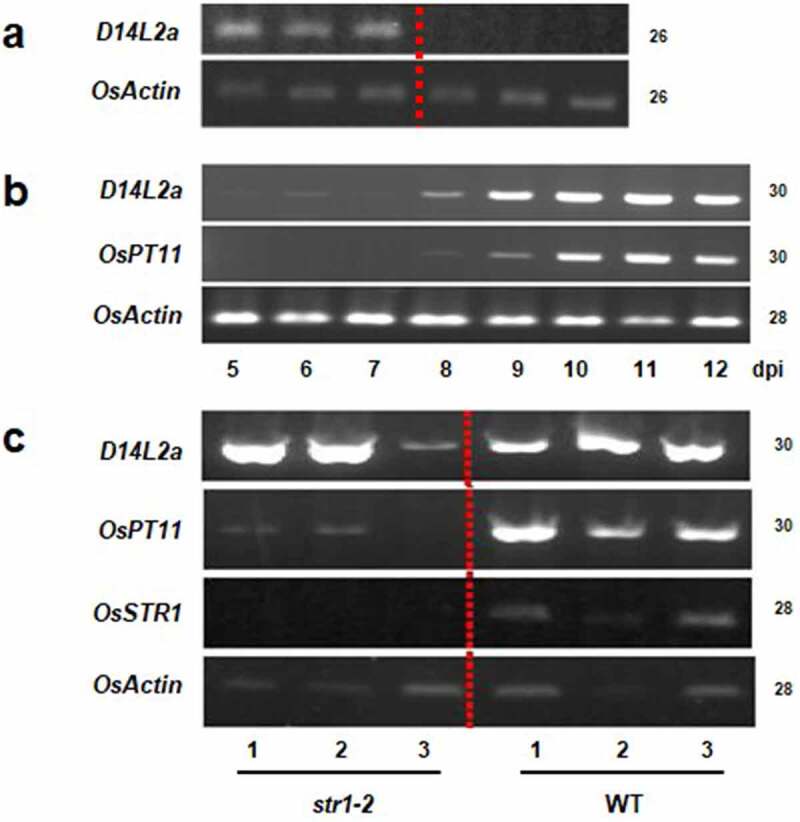
Expression of the *D14L2a* gene in rice roots. Cycle numbers of RT-PCR are indicated on the right. (a) Confirmation of *D14L2a* induction on the colonization of the AM fungus, using *D14L2a* cDNA_F and R primer. Total RNA was extracted from three biological replicates of mycorrhizal or non-mycorrhizal roots at 3 w post inoculation of the AM fungus. Left, mycorrhizal roots; right, non-mycorrhizal roots. Constitutive *OsActin* gene (Os03g0718100/LOC_Os03g50885) was amplified as a control using *OsActin* cDNA_F and R primer. (b) The time course of *D14L2a* induction. Rice roots were collected on 5–12 d post inoculation of the AM fungus, total RNA samples were extracted, and then RT-PCR was performed. Induction of *OsPT11* gene (Os01g0657100/LOC_Os01g46860), a marker of arbuscule development, was also examined, using *OsPT11* cDNA_F and R primer. (c) Expression of the *D14L2a* in *str1-2* mutant roots. Total RNA was extracted from three biological replicates each of mycorrhizal roots of *str1-2* (left) and wild type (right) at 3 w post inoculation of the AM fungus. Note that the transcript levels of *D14L2a, OsPT11* and *STR1* in the mutant are similar, much lower and none, respectively, compared to those of wild type.

Next, we characterized expression of *D14L2a* gene. The induction of *D14L2a* was confirmed *via* semi-quantitative RT-PCR (Figure 1a). Furthermore, a time-course experiment indicated that the transcript of *D14L2a* appears earlier than that of *OsPT11* (Figure 1b). Interestingly, the transcript level of *D14L2a* in mycorrhizal roots of *str1-2* mutant, which has a T-DNA insertion in the third exon of the *STR1* gene and shows stunted arbuscule growth,^26^ was nearly identical to that of wild type (Figure 1c). These results indicate that *D14L2a* gene was induced by infection of AM fungus, independently of arbuscule development.

## *Localized expression of* D14L2a

**Figure 2.**
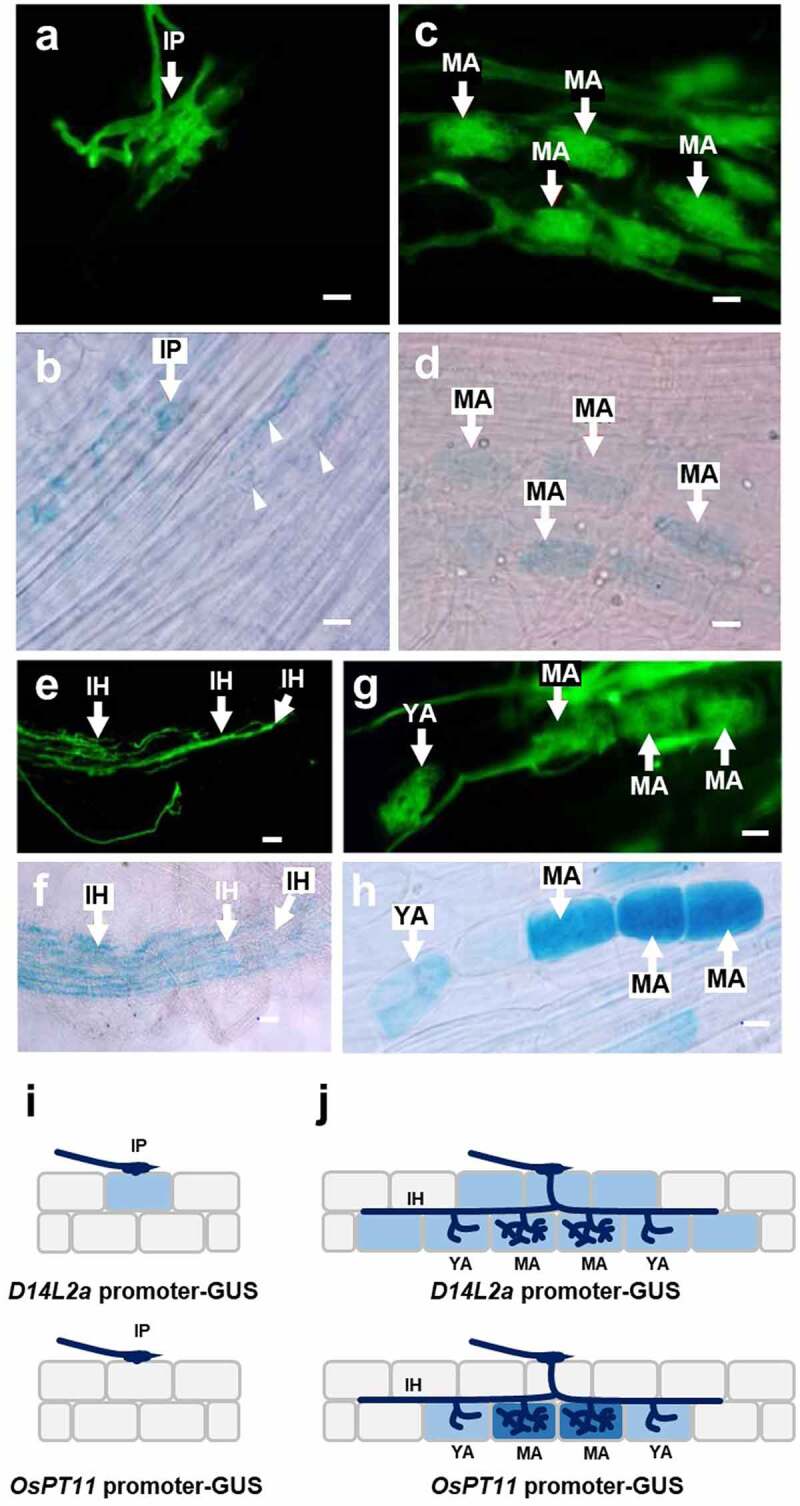
Double staining with wheat germ agglutinin-Alexa Fluor 488 conjugate and 5-bromo-4-chloro-3-indolyl-β-D-glucuronide of AM roots transformed by the *D14L2a* promoter-GUS. Rice roots were collected on 10–15 d post inoculation of the AM fungus. Scale bars = 50 µm. (a and b) Fungal structure and GUS staining, respectively, around the infection point (IP) of the AM fungus. Arrowheads indicate the position of GUS staining in cortical cells. (c and d) Detection of GUS activity in mature arbuscule (MA)-containing cells. (e and f) Detection of GUS activity around the elongating intercellular hyphae (IH) of the AM fungus. (g and h) Detection of weak and strong GUS activity in young arbuscule (YA)- and mature arbuscule (MA)-containing cells, respectively, in the AM roots of positive control, *OsPT11* promoter-GUS transformant. (i and j) A model illustrating the infection process of AM fungus and *D14L2a* expression at early colonization stage (i) and later stage (j)

We further examined spatial pattern of *D14L2a* expression. The transgenic rice roots with the *D14L2a* promoter-GUS construct exhibited GUS activity in epidermal/cortical cells around the infection point of the AM fungus (Figure 2a and b). The activity was detected again in the cortical cells that contain mature arbuscules (Figure 2c and d). The GUS activity was also detected in the cortical cells near the elongating intercellular hyphae with no visible arbuscules (Figure 2e and f). Alternatively, the transgenic roots with the *OsPT11* promoter-GUS construct exhibited both weak and strong GUS activities limited only in young arbuscule- and mature arbuscule-containing cells, respectively (Figure 2g and h). As expected,^31^ GUS activity was not detected in early colonization stage (e.g., epidermal cells). The intensity of the blue color among cells of *D14L2a* promoter-GUS and *OsPT11* promoter-GUS is different, indicating that the area and cell specificity of the expressions of two genes are different. As illustrated in Figure 2i and j, these results prove accumulation of *D14L2a* transcript not only in arbuscule-containing cells but also in other cortical/epidermal cells without arbuscules.

## *Hydrolysis of* rac*-GR24*

**Figure 3.**
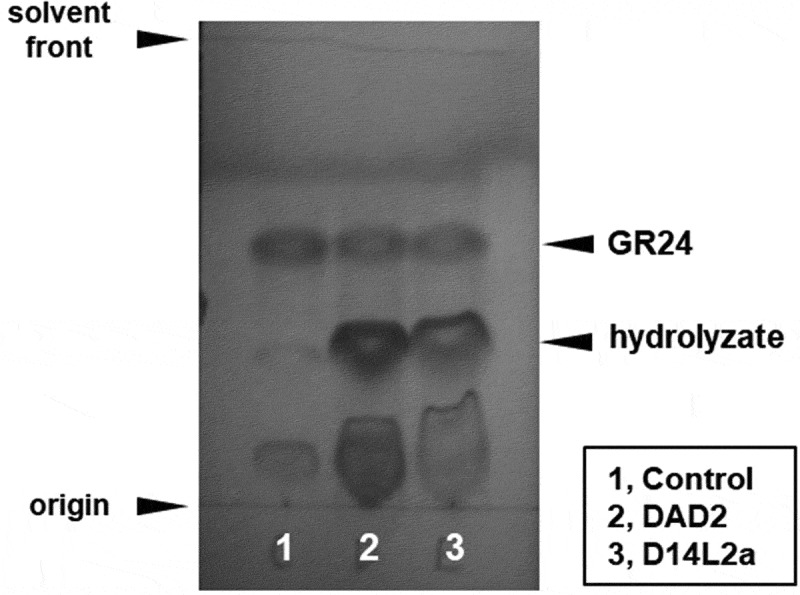
Detection of *rac*-GR24 hydrolysis by recombinant proteins with the aid of thin-layer chromatography. *rac*-GR24 (1 mM) was incubated with 73 µM of recombinant protein at 25°C for 18 h, extracted with ethyl acetate, concentrated under N_2_, spotted, and developed on silica gel 60 F_254_ plates (Merck, Darmstadt, Germany) using chloroform/acetone 4:1 (v/v) containing 5/1000 acetic acid. After development, the plates were air-dried at room temperature overnight, and then the spots were visualized under UV light.

In order to gain insight into the relationship between the expression of *D14L2a* and its function, we investigated the hydrolase activity of the protein. Because recombinant D14L2a and DAD2, a positive control, were accumulated in *E. coli* cells (Supplementary Figure S1a and c), we purified them (Supplementary Figure S1b and d) and examined their ability to hydrolyze the synthetic *rac*-GR24. As presented in Figure 3, D14L2a degraded *rac*-GR24. We also tried preparing rice D14 protein as the second positive control, but the recombinant D14 was not accumulated in the *E. coli* cells for an unknown reason.

## Discussion

The microarray experiments indicated that gene expression related to SL production in rice roots is elevated during AM symbiosis (Table 1), which is consistent with the previous report.^10^ We also found that the expression level of the rice *D14L2a* gene was significantly induced during the colonization of the AM fungus in roots (Table 1 and Figure 1a). The RiceXPro database indicated that *D14L2a* expression was negligible in non-mycorrhizal roots compared to that in leaves, stems and glumes of field-grown rice plants (https://ricexpro.dna.affrc.go.jp/GGEP/graph-view.php?featurenum=36077)^35^ while the database does not contain AM root data. Induction of *D14L2a* occurred slightly earlier than the appearance of *OsPT11* (Figure 1b, Figure 2), which is specifically expressed in arbuscule-containing cells.^30,31^ *D14L2a* was also induced in a *str1-2* mutant in which AM fungi cannot develop arbuscules well because of deficiency of plant-derived lipids (Figure 1c).^26,32–34^ Also, the expression of *D14L2a* was detected around the infecting and elongating hyphae of the AM fungus (Figure 2). In this context, it is noteworthy that while the expression level of *D14L2a* in entire mycorrhizal roots was higher than that of arbuscule-containing cell-specific *OsPT11* (Supplementary Table S2), the intensity of GUS staining in mature arbuscule-containing cells of *D14L2a* was much lower than that of *OsPT11* (Figure 2). Taken together, it was indicated that D14L2a functions not only in mature arbuscule-containing cells but also in other cortical/epidermal cells prior to the initiation of arbuscule formation in rice roots. Our results are in contrast to the claim of Ho-Plágaro et al.^20^ that the function of SlDLK2 is restricted in the regulation of arbuscule life cycle.

Although the ligand of D14L2 is still unknown, D14L2 is known to have catalytic triad (Ser, Asp, and His) residues.^19^ This study also demonstrated that recombinant D14L2a hydrolyzes *rac*-GR24 (Figure 3), strongly suggesting that D14L2a can work as a receptor and transduce some signaling. Because we did not quantify the band intensity on the thin-layer chromatography plate, the hydrolytic activity of D14L2a cannot be compared to that of DAD2, a typical member of the D14 clade that hydrolyzes both GR24^5DS^ and GR24*^ent^*^−5DS^.^12^

Since the discovery that a rice mutant lacking *D14L* does not respond to AM fungi,^22^ the signal transduction *via* D14L has attracted considerable attention among researchers on AM symbiosis.^10^ Notably, the transcript level of *D14L2* is elevated several folds during the D14L signaling, but the meaning of this elevation is unclear.^18,36^ Therefore, we cannot exclude the possibility that the high induction of *D14L2a* is, in part, a secondary effect of the D14L signaling during the colonization of AM fungi. Interpretation of the *rac*-GR24 hydrolysis also requires caution as it may represent non-natural strigolactone signaling.^29^ Nevertheless, because *D14L2* was expressed around the developing hyphae of AM fungi (Figure 2), it is tempting to speculate that not only D14L but also D14L2 are involved in the preliminary rearrangement in the host cells for AM colonization. No discernible change was observed in the morphology of Arabidopsis mutant lacking *D14L2*,^18^ but Arabidopsis is a non-mycorrhizal plant. Our view is that the mechanism of the D14L2 action, and that of D14L, should be elucidated carefully using mycorrhizal plants, such as rice.

If D14L2 is a signal transducer in AM symbiosis, how does it work? As previous reports already pointed out, it is unlikely for D14L2 to associate with D3/MAX2, a component of ubiquitin ligase, and to target other proteins such as D53.^19,21^ Nevertheless, Ho-Plágaro et al. made a breakthrough, demonstrating interaction of tomato SlDLK2 with a DELLA (aspartic acid–glutamic acid–leucine–leucine–alanine) protein SlGAI1 *via* co-immunoprecipitation and split-luciferase assay.^20^ Because DELLA proteins are a group of the GRAS (GIBBERELIC ACID INSENSITIVE REPRESSOR OF *ga1-3* SCARECROW) family of transcription factors, it is possible for D14L2 to capture and hydrolyze some compounds, and then interact with one or several GRAS regulators, ultimately modulating expression of many plant genes. Although we assume that D14L2 may be involved in the earlier process than the development of arbuscule, the mechanism of D14L2 action remains to be elucidated in the future.

## Supplementary Material

Supplemental Material
