# Supplementary material for: Localized expression of the Dwarf14-like2a gene in rice roots on infection of arbuscular mycorrhizal fungus and hydrolysis of rac-GR24 by the encoded protein
Source: Plant Signal Behav. 2021 Dec 14;16(12):2009998. doi: 10.1080/15592324.2021.2009998 (PMC9208777; doi:10.1080/15592324.2021.2009998)
Supplement: Supplemental Material [file KPSB_A_2009998_SM5652.zip › Supplementary FigS1.pdf]

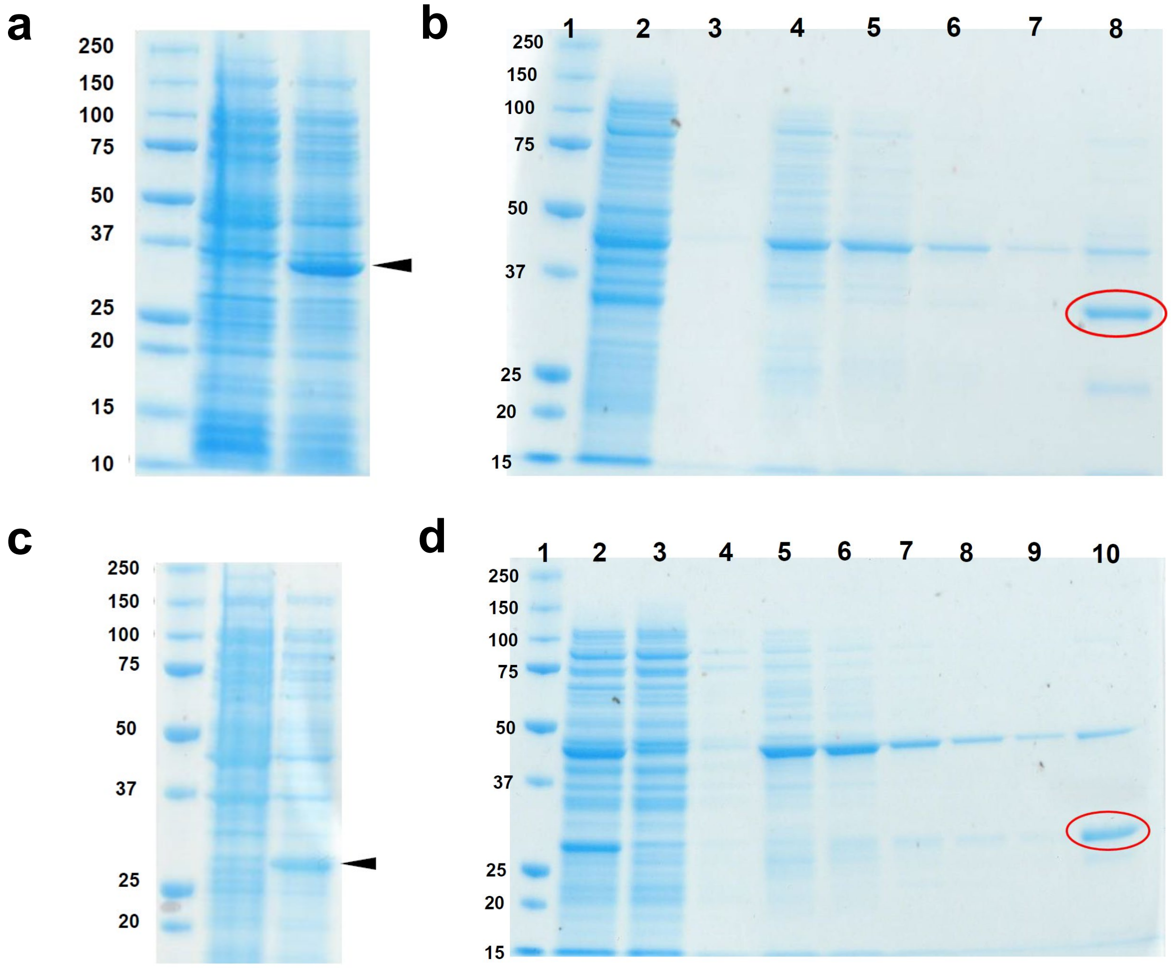

Supplementary Fig. S1

Accumulation and purification of the recombinant proteins. (a) Detection of D14L2a *via* SDS-PAGE. Left, molecular-size marker (the size of each band is given in kDa); center, proteins of *E. coli* BL21 before induction; right, those after induction. D14L2a is indicated by an arrowhead. (b) Lane 1, molecular-size marker; Lane 2, crude extract of D14L2a-accumulating *E. coli*; Lane 3, wash without imidazole; Lanes 4–7, wash with 20-mM imidazole; Lane 8, eluate with 150-mM imidazole. D14L2 is circled red. (c) Detection of DAD2. Left, molecular-size marker (in kDa); center and right, before and after induction, respectively. DAD2 is indicated by an arrowhead. (d) Lane 1, molecular-size marker; Lane 2, crude extract of DAD2-accumulating *E. coli*; Lane 3, crude extract of *E. coli* before the accumulation of proteins; Lane 4, wash without imidazole; Lanes 5–9, wash with 20-mM imidazole; Lane 10, eluate with 150-mM imidazole. DAD2 is circled.
