# Supplementary material for: Localized expression of the Dwarf14-like2a gene in rice roots on infection of arbuscular mycorrhizal fungus and hydrolysis of rac-GR24 by the encoded protein
Source: Plant Signal Behav. 2021 Dec 14;16(12):2009998. doi: 10.1080/15592324.2021.2009998 (PMC9208777; doi:10.1080/15592324.2021.2009998)
Supplement: Supplemental Material [file KPSB_A_2009998_SM5652.zip › Supplementary Table S1.pdf]

Supplementary Table S1

PCR primers

Gene name	Direction	Sequence (5' – 3')
D14L2a cDNA_F	Forward	GGTGGAAAGGAGAGGTGGTC
_R	Reverse	GCAAGCACCACGCTAATTACT
OsPT11 cDNA_F	Forward	GCTCCTGTACGCGCTCACTT
_R	Reverse	TGCGTGCATGGATGTCTGCCATTC
STR1 cDNA_F	Forward	GCTCTCCACACTGGACATCA
_R	Reverse	CCAAACACATATACTCCACATATGCCG
OsActin cDNA_F	Forward	CTCTCAGCACATTCCAGCAGATG
_R	Reverse	GGACGGCGATAACAGCTCCTC
D14L2a promoter_F	Forward	CCAAAATAGGAGCATCTATCACGCTT
_R	Reverse	CACTCTCGCGTTGGCGCGCCACATCTT
OsPT11 promoter_F	Forward	CACCTTCCAGCAGCAGTAGAGC
_R	Reverse	CGCGTCCAGCACCGCCAGGTTC
D14L2a coding region_F	Forward	GGGAATTCATGAAGAAGATGTGGCGCGCC
_R	Reverse	CCCTGCAGCTATATGGCAACATCGATGCC
DAD2 coding region_F	Forward	TACCCTCGAGATGGGACAGACCCTTTTAGA
_R	Reverse	TCGACAAGCTTCACCTATGTGAAAGAGCTC
