# Supplementary material for: Localized expression of the Dwarf14-like2a gene in rice roots on infection of arbuscular mycorrhizal fungus and hydrolysis of rac-GR24 by the encoded protein
Source: Plant Signal Behav. 2021 Dec 14;16(12):2009998. doi: 10.1080/15592324.2021.2009998 (PMC9208777; doi:10.1080/15592324.2021.2009998)
Supplement: Supplemental Material [file KPSB_A_2009998_SM5652.zip › Supplementary Table S2.pdf]

accessions	Description	myc-.1	myc-.2	myc-.3	myc+.1	myc+.2	myc+.3	myc- Avr.	myc+ Avr.	myc+/myc-
Os09g0568700|AK109	Germin family protein.	30	21	82	55625	53445	56649	44.2568933	55239.5133	1248.156144
**Os01g0657100|AK062**	**OsPT11**	**10**	**8**	**7**	**10725**	**10148**	**9677**	**8.21114467**	**10183.2297**	**1240.171752**
**Os09g0401100|Os09g**	**OsABCG21 (STR1)**	**17**	**21**	**6**	**12140**	**12105**	**10759**	**14.5461377**	**11667.9633**	**802.1348072**
Os04g0134800|Os04g	Haem peroxidase, plant/fungal/bacterial family protein.	74	67	52	33078	30048	27154	64.4299233	30093.19	467.0685365
**Os01g0657100|AF536**	**OsPT11**	**41**	**19**	**15**	**12893**	**11240**	**10387**	**24.9256267**	**11506.7167**	**461.6420209**
Os09g0568500|AK108	Germin family protein.	10	6	6	1681	3025	3067	7.55811033	2591.29333	342.8493657
Os03g0103000|CI4411	(No Hit)	9	7	6	1939	1590	1520	7.12364467	1682.955	236.2491504
Os04g0429600|Os04g	Cellulose synthase CesA-1.	18	10	8	3499	1856	3168	12.213227	2841.12333	232.6267524
Os05g0579200|AK106	AMP-dependent synthetase and ligase domain containing protein.	9	7	6	1813	1675	1728	7.64085667	1738.75	227.5595625
Os03g0103000|CI4471	(No Hit)	9	7	6	1674	1736	1513	7.417645	1641.051	221.2361201
Os03g0582300|AK121	SCAMP family	33	24	24	6338	4146	5797	26.9312133	5427.035	201.5146861
Os02g0533300|CI4157	Carbonic anhydrase, eukaryotic family protein.	49	62	51	9223	7863	9733	53.8360767	8939.59967	166.0522129
Os12g0138100|Os12g	GRAS transcription factor domain containing protein.	69	78	71	10239	10457	9700	72.7247533	10131.693	139.3156049
Os03g0735900|Os03g	Phospholipid/glycerol acyltransferase family protein.	82	71	53	9315	8189	8072	68.5133467	8525.477	124.435273
Os01g0255100|AK058	Soluble epoxide hydrolase.	39	24	20	3419	2732	3372	27.3949033	3174.312	115.8723563
Os01g0303100|AK062	Chitinase (EC 3.2.1.14).	19	20	9	1819	1770	1936	16.00089	1841.474	115.0857234
Os03g0703600|AK107	Conserved hypothetical protein.	10	17	8	1338	1132	1449	11.704045	1306.24367	111.6061726
Os10g0331700|CI1471	(No Hit)	23	22	11	1978	1905	1765	18.80671	1882.76533	100.111361
Os02g0184700|CI2231	Cytochrome P450 family protein.	108	95	132	11232	8928	10940	111.539187	10366.532	92.94071716
Os10g0525000|AK105	Cytochrome P450 family protein.	85	82	75	7270	5158	6480	80.60886	6302.87333	78.19082584
Os10g0525000|AK069	Cytochrome P450 family protein.	82	70	69	6344	4713	5944	73.44842	5667.126	77.1579021
Os10g0525000|AK104	Cytochrome P450 family protein.	77	93	58	6446	4562	5905	75.8210633	5637.47133	74.35231169
Os01g0870300|AB083	OsAMT3.1	213	308	285	21923	19684	18215	268.853133	19940.9367	74.17037108
Os05g0372300|AK120	Cytochrome P450 monooxygenase CYP72A5 (Fragment).	22	31	58	3087	2003	2922	37.13734	2670.70367	71.91424229
Os11g0594700|CI0276	Protein of unknown function DUF538 family protein.	8	6	5	433	365	529	6.21653533	442.204467	71.13358856
Os06g0564700|AK070	Cysteine synthase (EC 4.2.99.8).	11	16	37	1730	938	1764	21.4350067	1477.62183	68.93498361
Os06g0654600|Os06g	Protein kinase domain containing protein.	24	14	24	1640	990	1264	20.56402	1297.7469	63.10764627
Os11g0139600|AK073	Scarecrow-like 9 (Fragment).	24	29	15	1443	1328	1179	22.81007	1316.71767	57.72527952
Os01g0870300|AK120	OsAMT3.1	782	1047	1019	55161	52939	53621	949.2081	53907.0967	56.79165261
**Os05g0590300|AK067**	**Dwarf14-like2a**	**271**	**260**	**399**	**18187**	**13211**	**19223**	**309.9603**	**16873.5167**	**54.43767046**
Os02g0281000|AK062	Protein phosphatase 2C family protein.	7	6	5	6	966	10	6.045934	327.222409	54.12272264
Os06g0602900|CI5373	(No Hit)	208	176	349	13767	9332	12998	244.260167	12032.3893	49.26054664
Os06g0602900|CI5373	(No Hit)	190	163	342	12849	8651	12584	231.465833	11361.648	49.08563755
Os07g0649500|CI5209	ARM repeat fold domain containing protein.	28	28	22	1558	908	1310	25.7482167	1258.7942	48.88859746
Os11g0594800|CI3937	Protein of unknown function DUF538 family protein.	10	10	8	451	271	612	9.24808467	444.752167	48.09127324
Os08g0519900|Os08g	Exo70 exocyst complex subunit family protein.	43	21	20	1596	1176	1261	28.0612867	1344.328	47.9068553
Os08g0566700|AK119	Conserved hypothetical protein.	23	18	33	1368	1023	1118	24.4182533	1169.393	47.89011663
Os12g0223700|Os12g	Hypothetical protein.	9	18	6	552	380	593	10.6987067	508.343467	47.51447839
Os07g0575500|CI4630	Glycoside hydrolase, family 20 protein.	37	39	31	1809	1498	1530	35.7056667	1612.25133	45.15393448
Os07g0528900|Os07g	Syntaxin-like protein (Fragment).	9	7	20	673	296	585	11.906889	517.780667	43.48580613

Os10g0481500|Os10g	FLOT	82	65	47	3204	2853	2261	64.69074	2772.959	42.86485206
Os01g0332200|CI4647	(No Hit)	115	108	198	6438	4076	6151	140.412633	5555.27433	39.56392101
Os01g0229700|Os01g	CERBERUS	68	52	33	2316	1684	1899	50.9344867	1966.47167	38.60786268
Os04g0190000|AK107	Conserved hypothetical protein.	9	7	6	7	819	11	7.225131	278.896869	38.60094292
Os06g0613600|CI4222	Cytochrome P450 family protein.	371	233	259	11886	9461	11444	287.863767	10930.3173	37.970452
Os01g0332200|CI4647	(No Hit)	132	122	224	6711	4334	6443	159.244367	5829.43233	36.60683549
Os03g0643700|AK110	Glutathione transferase (EC 2.5.1.18).	456	385	518	17020	15635	16385	453.0719	16346.9933	36.08035134
Os05g0137700|Os05g	Thioesterase superfamily domain containing protein.	74	58	51	2419	1633	2298	61.1241033	2116.65633	34.62883246
Os01g0800700|CI5378	Hypothetical protein.	8	7	6	336	131	224	6.86971267	230.317067	33.52644832
Os02g0630300|AK072	2OG-Fe(II) oxygenase domain containing protein.	89	87	89	3648	1996	3174	88.3444267	2939.39533	33.27199512
Os03g0138200|AK105	Cytochrome P450 family protein.	8	6	5	6	590	10	6.212817	201.881163	32.49430385
Os03g0100800|AK068	Plasma membrane H+-ATPase.	305	331	337	12068	8949	10564	324.151433	10526.9527	32.47541607
Os01g0860400|AK111	Acidic endochitinase precursor (EC 3.2.1.14).	906	1167	901	34568	28634	31258	991.261367	31486.72	31.76429654
Os07g0564100|AK107	UDP-glucuronosyl/UDP-glucosyltransferase family protein.	49	28	31	1034	1226	926	35.8261167	1061.7383	29.63587457
Os01g0561600|AK069	Cytochrome P450 family protein.	7	6	5	107	270	117	5.89744533	165.006133	27.9792561
Os12g0263800|AK066	Pinoresinol-lariciresinol reductase TH1.	10	8	6	17	15	582	7.832843	204.547953	26.11413931
Os07g0120300|Os07g	Protein of unknown function DUF538 family protein.	217	261	277	7202	4532	7713	251.7683	6482.21667	25.74675472
Os04g0405300|AK110	Stem secoisolariciresinol dehydrogenase (Fragment).	2328	2637	3535	83674	50870	81469	2833.15667	72004.3733	25.41489293
Os05g0217800|AK104	BURP domain containing protein.	8	8	5	16	505	16	7.08379767	178.810033	25.24211472
Os04g0473900|CI1586	(No Hit)	76	68	51	1642	1679	1515	64.9950133	1612.02133	24.80223098
Os01g0724500|AK067	PDR-like ABC transporter.	391	360	638	13463	8942	12057	463.341433	11487.452	24.79262845
Os04g0448500|CI4690	Peptidase A1, pepsin family protein.	36	15	18	604	618	479	22.9129933	566.9972	24.7456625
Os12g0193100|AK101	Subtilisin inhibitor I (ASI-I) [Contains: Subtilisin inhibitor II (ASI-II)].	154	151	209	4798	3130	4686	171.492233	4204.61	24.51778671
Os07g0107500|Os07g	Plant disease resistance response protein family protein.	80	79	100	2662	1426	2267	86.4026167	2118.16967	24.51511017
Os03g0611200|AK065	Aldo/keto reductase family protein.	8	18	6	290	185	286	10.4760487	253.555967	24.20339717
Os02g0805400|CI5162	Galactose oxidase, central domain containing protein.	125	86	69	2570	1901	2140	93.3201067	2203.76567	23.6151216
**Os01g0595600|AK066**	**Dwarf14-like2b**	**241**	**236**	**237**	**4961**	**5948**	**5922**	**238.168533**	**5610.26467**	**23.55586016**
Os09g0459500|AB118	Hypothetical protein.	66	61	50	1417	1360	1408	59.24053	1394.966	23.54749358
Os04g0481700|AK107	Multidrug resistance p-glycoprotein; ABC transporter-like protein.	454	376	558	12399	8929	11228	462.539633	10852.1097	23.46201035
Os05g0372900|AK072	Conserved hypothetical protein.	95	83	130	2596	2203	2406	102.54971	2401.336	23.41631195
Os02g0208300|CI4445	PDR5-like ABC transporter.	71	111	82	2217	1617	2271	87.76164	2034.99267	23.18772378
Os10g0378100|AK120	Cytochrome P450 family protein.	165	158	275	5149	3201	5457	199.270333	4602.20033	23.09526088
Os03g0799900|AK111	OsPHFmyc	51	61	67	1504	1039	1528	59.5047233	1357.00067	22.80492355
Os09g0376600|Os09g	Protein kinase domain containing protein.	143	188	126	4023	3037	3301	152.3168	3453.59	22.67373002
Os10g0469100|AK121	Conserved hypothetical protein.	9	7	6	7	496	11	7.58867	171.386649	22.58454362
Os03g0136400|AK121	OsPT7	9	6	6	6	435	10	6.944452	150.365172	21.65256126
Os11g0514500|CI4514	Sorghum bicolor leucine-rich repeat-containing extracellular glycoprote	41	97	43	839	1107	1853	60.0248733	1266.53777	21.10021557
Os07g0120100|AK063	Protein of unknown function DUF538 family protein.	50	59	67	1250	943	1509	58.49712	1233.91043	21.09352449
Os10g0157400|CI4889	(No Hit)	9	6	7	58	332	49	7.38911167	146.507593	19.82749753
Os11g0659600|AK107	Pectinesterase family protein.	23	13	15	7	995	10	17.1638567	337.203694	19.64614948
Os01g0930900|CI2392	Tropinone reductase homolog (EC 1.1.1.-) (P29X).	164	153	160	3464	2442	3458	158.9002	3121.07133	19.64170802

Os10g0525000|AK099	Cytochrome P450 family protein.	48	51	25	850	742	820	41.38107	803.953633	19.42805329
Os11g0582000|CI2019	(No Hit)	8	7	5	121	125	135	6.609348	126.794033	19.18404559
Os09g0466300|AK102	GRAM domain containing protein.	71	75	76	1513	1261	1445	73.9465967	1406.38167	19.01888295
Os06g0244100|AK063	TPR-like domain containing protein.	9	7	6	6	14	390	7.23379367	136.64372	18.88963467
Os04g0540300|Os04g	Phytochelatin synthetase-like conserved region family protein.	38	31	32	709	538	618	33.6386467	621.516233	18.47625558
osa-miR426|Os05|NA|	miRNA	14	8	5	11	47	437	9.11379533	165.26692	18.13370983
Os10g0149200|CI4790	(No Hit)	10	8	7	7	15	422	8.29259933	148.019555	17.84959682
Os02g0159700|AK111	Electron transport protein SCO1/SenC family protein.	9	7	6	6	14	362	7.17498767	127.319137	17.74485791
Os10g0378000|Os10g	E-class P450, group I family protein.	685	676	1001	15902	9700	15435	787.412533	13679.049	17.37215046
Os04g0534600|AK073	Peroxisomal biogenesis factor 11 family protein.	31	23	16	387	440	354	23.0659867	393.527867	17.0609596
Os11g0687100|AK065	von Willebrand factor, type A domain containing protein.	280	235	355	2959	4785	7076	289.8142	4940.00267	17.04541277
Os04g0180400|AK071	Cytochrome P450 CYP99A1 (EC 1.14.-.-) (Fragment).	50	44	34	626	716	831	42.7190267	724.317733	16.95538943
Os04g0179200|AK106	Stem secoisolariciresinol dehydrogenase (Fragment).	51	67	49	868	937	990	55.3880333	931.5818	16.81918898
Os06g0273400|AK108	Conserved hypothetical protein.	9	7	6	18	338	11	7.33790133	122.627837	16.71156794
Os03g0339300|AK104	Peroxidase (EC 1.11.1.7).	12	8	8	14	438	10	9.31388133	153.969822	16.53122003
Os02g0605900|AK064	Chitinase (EC 3.2.1.14) A.	10	27	16	289	257	325	17.7057757	290.151933	16.38741724
Os04g0179200|AK120	Stem secoisolariciresinol dehydrogenase (Fragment).	52	74	51	858	963	1048	58.6622867	956.324767	16.30220745
Os11g0706000|Os11g	GRAS transcription factor domain containing protein.	84	75	58	1209	1209	1069	72.1421767	1162.30833	16.11135659
Os07g0249800|AK110	IAA-amino acid hydrolase 1 (EC 3.5.1.-).	9	13	6	125	158	164	9.46936	149.2977	15.76639815
Os01g0700500|AK072	Cytochrome P450 family protein.	1125	1017	1658	22117	15324	22192	1266.78333	19877.9767	15.69169419
Os09g0472700|CI2798	(No Hit)	44	41	31	583	493	738	38.63245	604.648067	15.65130005
Os03g0638300|CI5276	(No Hit)	12	7	6	335	31	11	8.325662	125.632383	15.08977705
osa-miR169e|Os02|NA	miRNA	8	6	5	6	271	10	6.41514067	95.6330357	14.90739496
Os10g0186800|CI4328	Nucleic acid-binding OB-fold domain containing protein.	11	7	6	6	329	11	7.82202933	115.410566	14.75455551
Os10g0542100|AK063	EC protein I/II (Zinc-metallothionein class II).	21	10	7	192	208	162	12.7108453	187.5094	14.75192209
Os12g0582900|AK109	AP2 domain containing protein RAP2.11 (Fragment).	25	34	30	436	418	436	29.4204333	430.074467	14.61822339
Os07g0604100|CI4539	(No Hit)	8	11	5	328	12	10	8.016097	116.72743	14.56162893
Os08g0120600|AK069	Aldolase C-1.	152	247	164	3046	2232	2896	187.504633	2725.022	14.53309154
Os04g0658700|Os04g	Protein kinase domain containing protein.	13	7	11	202	134	118	10.4461267	151.1653	14.47094266
Os11g0607900|CI4412	Protein kinase domain containing protein.	7	10	5	69	103	152	7.53914033	108.02836	14.32900241
Os01g0883800|CI0023	Gibberellin 20-oxidase.	10	8	10	209	89	114	9.64264267	137.45841	14.25526329
Os02g0634700|AK071	Serine carboxypeptidase II-1 precursor (EC 3.4.16.6) (CP-MII.1) (Fragr	525	549	584	8263	7312	8013	552.914333	7862.46933	14.22004976
Os08g0137900|CI4370	(No Hit)	10	7	7	98	79	154	7.85747867	110.34614	14.04345397
Os02g0278700|AK100	Kaurene synthase A.	53	85	132	1474	723	1590	90.2547833	1262.36237	13.98665334
Os11g0286300|Os11g	Terpenoid cylases/protein prenyltransferase alpha-alpha toroid domain	53	28	36	616	433	584	38.99559	544.260367	13.95697223
Os08g0460000|AK065	Germin-like protein 5 (Germin-like protein 1).	15	17	7	14	519	9	12.9744177	180.813468	13.93615284
Os11g0226800|AF456	NBS-LRR disease resistance protein homologue.	9	7	6	7	14	288	7.46545667	102.726824	13.76028673
Os01g0602400|AK107	Cytochrome P450 monooxygenase CYP72A5 (Fragment).	25	34	22	320	368	403	26.89661	363.494767	13.51451974
Os01g0323100|AK065	Pto kinase interactor 1.	50	74	67	919	718	935	63.5963267	857.048433	13.47638265
Os03g0284700|AK069	Hypothetical protein.	17	15	15	213	210	215	15.8046767	212.597767	13.4515733
Os05g0215000|AK060	BURP domain containing protein.	67	74	61	964	795	951	67.4214267	903.161033	13.39575678

Os02g0661200|CI5142	(No Hit)	8	6	6	6	13	253	6.83076633	90.8411423	13.29882152
Os01g0700500|AK119	Cytochrome P450 family protein.	992	803	1377	14840	11703	15579	1057.41707	14040.38	13.2779964
Os05g0215000|AK104	BURP domain containing protein.	61	79	47	893	680	900	62.22521	824.352367	13.24788404
Os11g0595000|Os11g	Protein of unknown function DUF538 family protein.	18	31	17	315	198	354	21.8763533	289.227867	13.22102739
Os03g0386600|AK069	Conserved hypothetical protein.	8	6	5	226	12	10	6.28854767	82.448395	13.11088019
Os08g0547900|AK103	Cytochrome P450 family protein.	677	923	951	12608	8290	12074	850.348867	10990.3797	12.92455379
Os05g0394000|CI3128	(No Hit)	8	6	5	6	224	10	6.19457167	79.8459267	12.88966065
Os05g0141300|Os05g	Transferase family protein.	53	48	45	678	503	686	48.46841	622.323433	12.83977406
Os02g0569900|AK105	Cytochrome P450 family protein.	113	89	87	937	1412	1325	95.9727067	1224.45903	12.75840888
Os06g0191700|CI0867	Cytochrome P450 family protein.	469	673	697	8437	7080	7747	613.010067	7754.66567	12.65014408
Os04g0355300|Os04g	Conserved hypothetical protein.	18	19	5	8	518	10	14.1514467	178.977085	12.64726419
Os02g0546800|CI2263	(No Hit)	10	7	7	7	281	12	7.906192	99.9528163	12.64234619
Os03g0226800|CI1447	C-5 cytosine-specific DNA methylase family protein.	7	6	5	81	63	89	6.18019233	77.6498467	12.56430908
Os06g0582600|AK067	Cysteine proteinase.	10	8	6	93	82	120	7.88106133	98.33113	12.47688932
osa-miR169i|Os08|NA|	miRNA	10	12	10	13	16	370	10.667813	132.85668	12.45397534
Os06g0560700|AK063	Lipolytic enzyme, G-D-S-L family protein.	9	7	6	7	100	173	7.48534333	93.1156493	12.43972991
Os05g0215000|AK104	BURP domain containing protein.	94	106	82	1292	937	1263	94.0356833	1164.02573	12.37855346
Os01g0245700|CI0982	(No Hit)	8	9	5	59	12	203	7.41416433	91.4486233	12.33431297
Os11g0224400|AK100	Hypothetical protein.	11	8	7	8	299	13	8.67368333	106.855017	12.31945106
Os09g0433900|AK107	Alanine aminotransferase.	58	28	64	690	446	703	50.0782333	612.882967	12.23851014
Os05g0215000|AK103	BURP domain containing protein.	65	83	65	957	707	935	70.9418367	866.387267	12.21264218
Os07g0137500|AK119	Conserved hypothetical protein.	9	7	6	6	14	245	7.23338667	88.305708	12.20807238
Os07g0575600|AK101	Resistance protein candidate (Fragment).	69	105	85	1150	917	1073	86.3845733	1046.51263	12.11457779
Os06g0191800|AK106	Cytochrome P450.	304	414	477	4869	4275	4890	398.2905	4678.18167	11.74565215
Os06g0570600|Os06g	Ent-kaurene oxidase (EC 1.14.13.78) (AtKO1) (Cytochrome P450 701	42	43	75	782	347	719	53.22544	616.120567	11.57567822
Os04g0550600|AK109	Retinal pigment epithelial membrane protein family protein.	546	556	625	7232	5392	7276	575.543533	6633.15167	11.52502162
Os06g0251100|CI2495	Membrane attack complex component/perforin/complement C9 family	10	12	10	131	123	120	10.8200887	124.234	11.48179131
Os01g0546000|CI4326	(No Hit)	34	25	24	345	268	332	27.5273033	315.0359	11.44448827
Os09g0559500|CI0030	(No Hit)	9	7	12	13	291	11	9.22518333	105.202553	11.40384419
Os12g0274700|AK061	Ribulose-1,5-bisphosphate carboxylase/oxygenase small subunit (Frag	42	32	24	79	75	960	32.69962	371.231327	11.35277189
Os12g0154900|Os12g	Germin-like protein precursor.	54	48	48	728	332	627	49.86347	562.429867	11.27939685
Os10g0542100|AK102	EC protein I/II (Zinc-metallothionein class II).	24	15	11	194	205	161	16.5714167	186.611033	11.26101872
Os10g0118700|CI4334	(No Hit)	8	6	5	106	20	77	6.20153867	67.5987767	10.90032334
Os01g0546000|CI4326	(No Hit)	36	41	28	390	339	404	34.99064	377.5859	10.79105441
Os05g0215000|AK104	BURP domain containing protein.	93	81	85	975	796	998	86.3129933	922.994367	10.69357383
Os05g0215000|AK104	BURP domain containing protein.	91	92	70	960	775	933	84.2267433	889.475533	10.56048825
Os04g0557500|AB021	Basic helix-loop-helix dimerisation region bHLH domain containing prot	7	6	5	6	174	10	6.01697433	63.2221567	10.50730037
Os05g0149300|CI2771	1-aminocyclopropane-1-carboxylate oxidase.	8	7	6	6	198	11	6.84678267	71.707355	10.47314607
Os02g0571100|AK072	Terpene synthase, metal-binding domain containing protein.	18	14	6	65	178	151	12.5355947	131.207757	10.46681551
Os02g0491800|Os02g	Germin-like protein 3 (Fragment).	54	50	32	535	447	432	45.03474	471.3671	10.46674412
Os05g0215000|AK109	BURP domain containing protein.	76	83	78	915	714	856	79.14349	828.268467	10.46540236

Os07g0416900|AK070	Omega-6 fatty acid desaturase, endoplasmic reticulum isozyme 2 (EC	93	163	103	866	1291	1576	119.443597	1244.4133	10.41841785
Os04g0565200|AK111	Cis-zeatin O-glucosyltransferase.	297	253	269	2902	2459	3141	273.040533	2833.94067	10.379194
Os05g0577200|AK069	Esterase/lipase/thioesterase domain containing protein.	7	11	5	6	12	229	7.948044	82.0884473	10.32813197
Os03g0283000|AY332	Glutathione S-transferase.	8	6	5	6	12	176	6.38158767	64.657606	10.13189967
Os03g0258000|AK107	Resistance protein candidate (Fragment).	9	6	6	57	91	67	7.09819333	71.8238567	10.11861093
Os04g0653700|AK062	Hypothetical protein.	10	8	7	80	85	77	7.97703867	80.5913967	10.10292165
Os03g0164400|AK063	FAR1 domain containing protein.	386	159	156	212	6618	214	233.494067	2347.74937	10.05485664
Os12g0155000|Os12g	Germin-like protein precursor.	79	104	99	1193	541	1102	94.0513867	945.339167	10.05130493
Os01g0701500|Os01g	Cytochrome P450 family protein.	255	325	347	3794	2039	3409	309.072133	3080.8	9.967899619
Os07g0146800|AK106	Non-protein coding transcript, uncharacterized transcript.	9	7	9	16	220	12	8.35522767	82.78868	9.908608515
Os05g0153200|AK063	Peptidase M14, carboxypeptidase A family protein.	7	6	5	27	11	140	6.00539067	59.39299	9.8899461
Os07g0188000|AK100	Argonaute protein.	8	14	16	155	142	73	12.5225133	123.504637	9.862607719
Os10g0118800|CI2586	Cytochrome b561 family protein.	34	115	63	801	611	664	70.29143	691.8105	9.842031952
Os01g0778100|CI2181	(No Hit)	53	76	63	689	621	577	64.0171333	628.717433	9.821080711
Os12g0148700|AK107	Hypothetical protein.	11	15	14	141	120	126	13.2442533	128.860167	9.729515392
Os11g0707000|AF349	Ribulose-bisphosphate carboxylase activase (EC 6.3.4.-) (Fragments).	11	9	5	6	24	207	8.142614	78.823671	9.680389
Os10g0110800|AK070	Nitrate transporter (Fragment).	8	6	5	12	169	10	6.61110267	63.4519867	9.597791755
Os01g0585200|AK107	Conserved hypothetical protein.	16	33	25	240	205	268	24.7843567	237.440433	9.58025405
Os02g0754300|AK099	50S ribosomal protein L29, chloroplast precursor.	9	7	6	36	164	11	7.382057	70.4539	9.543938769
Os01g0111700|AK108	Conserved hypothetical protein.	19	16	5	120	111	159	13.621185	129.989367	9.543176065
Os03g0358800|Os03g	UDP-glucuronosyl/UDP-glucosyltransferase family protein.	253	253	225	2618	2101	2253	243.850033	2323.916	9.53010327
Os05g0268500|AK119	Serine carboxypeptidase II-2 precursor (EC 3.4.16.6) (CP-MII.2) (Fragr	8	9	5	6	12	188	7.248509	68.6009107	9.464140924
Os01g0584900|AY341	WRKY transcription factor 67.	8	6	6	50	63	78	6.72569167	63.4972767	9.441003218
Os05g0215000|AK104	BURP domain containing protein.	73	81	60	721	599	695	71.1843033	671.569667	9.434238101
Os08g0137900|CI5530	(No Hit)	12	10	15	111	70	161	12.134856	113.843673	9.381542998
Os10g0118700|CI4108	(No Hit)	8	6	6	107	16	66	6.74385867	63.0957133	9.356025452
Os05g0215000|AK104	BURP domain containing protein.	81	69	61	704	598	666	70.16473	655.815433	9.346796223
Os08g0174100|CI3124	Transferase family protein.	105	120	127	1136	999	1140	117.426267	1091.58817	9.295945427
Os05g0500600|AK070	GRAS transcription factor domain containing protein.	536	771	685	6748	5332	6242	664.011033	6107.087	9.197267355
Os11g0594000|CI5524	General substrate transporter family protein.	9	12	23	23	361	17	14.6174153	133.734673	9.148995926
Os01g0778100|CI4365	(No Hit)	79	82	74	723	683	731	78.1062567	712.5174	9.12241132
Os01g0371800|Os01g	Zn-finger, CCHC type domain containing protein.	22	19	14	18	474	13	18.48592	168.239697	9.100964229
Os12g0221400|AU075	Hypothetical protein.	10	8	7	61	55	105	8.18545433	73.7941167	9.015274371
Os01g0177400|AB056	GA 3beta-hydroxylase.	284	333	303	3197	2439	2646	306.743133	2760.676	8.999960227
Os05g0215000|AK104	BURP domain containing protein.	84	72	63	704	568	694	73.03992	655.417967	8.973421201
Os10g0331600|Os10g	UDP-glucuronosyl/UDP-glucosyltransferase family protein.	31	22	22	259	220	193	25.0080333	223.9597	8.955510296
Os07g0141400|AF052	23 kDa polypeptide of photosystem II.	8	10	5	78	60	70	7.72636133	69.0733033	8.939952502
Os04g0629600|AK099	Zn-finger, CCHC type domain containing protein.	26	31	38	51	776	25	31.8184467	283.80417	8.919485384
Os05g0170600|AK103	Hypothetical protein.	7	6	5	12	12	142	6.20111533	55.2621667	8.911649549
Os06g0210900|AK110	Lipase, class 3 family protein.	9	11	6	49	106	77	8.732255	77.33528	8.856278247
Os01g0627800|AK101	Cytochrome P450 monooxygenase CYP72A5 (Fragment).	69	48	30	261	548	486	48.8920867	431.418133	8.823884656

Os05g0551700|AK107	tRNA isopentenyltransferase family protein.	7	6	8	6	170	10	7.02104567	61.9349207	8.821324288
Os01g0177400|AB056	GA 3beta-hydroxylase.	300	335	304	3295	2316	2655	313.124233	2755.48867	8.799985352
Os01g0800500|AK066	Metallophosphoesterase domain containing protein.	194	285	261	2344	1992	2168	246.480067	2168.00633	8.795868821
Os03g0424000|AK107	Protein kinase domain containing protein.	9	7	14	7	15	251	10.3618523	91.132978	8.795046973
Os05g0215000|AK104	BURP domain containing protein.	79	72	78	700	593	727	76.6251533	673.582433	8.790617755
Os04g0559000|Os04g	Peptidase S8 and S53, subtilisin, kexin, sedolisin domain containing pr	613	550	534	5978	3891	5043	565.607033	4970.88667	8.788587082
Os06g0196500|CI2232	(No Hit)	10	7	7	86	55	64	7.83727733	68.3815667	8.725168673
Os04g0186800|Os04g	OsPT13	101	67	55	664	762	520	74.4208733	648.404433	8.712668964
Os01g0959200|CI4356	Abscisic stress ripening protein 1.	191	233	115	1636	1654	1393	179.8274	1561.08567	8.681022284
Os04g0179700|AK119	Terpene synthase.	16	11	17	91	146	141	14.54844	126.08294	8.666423342
Os12g0626500|CI2531	Seed maturation protein domain containing protein.	100	87	133	984	662	1101	106.727653	915.488367	8.577799081
Os10g0498100|AK071	Epoxide hydrolase family protein.	1593	1680	2202	16916	12448	17357	1824.88133	15573.4133	8.533932069
Os07g0452100|CI5356	Alpha-galactosidase (EC 3.2.1.22) (Fragment).	9	7	7	75	50	73	7.76531167	66.07727	8.509287565
Os01g0177400|AB056	GA 3beta-hydroxylase.	322	368	313	3369	2403	2745	334.4433	2838.953	8.488592835
Os09g0452900|AK068	Glycosyl transferase, family 31 protein.	129	178	170	1510	1003	1513	158.7569	1341.97767	8.453035217
Os06g0486300|Os06g	Mlo.	7	12	21	145	93	106	13.588199	114.589847	8.433041543
Os03g0838500|AK121	Proteinase inhibitor I9, subtilisin propeptide domain containing protein.	8	6	7	6	12	161	7.11135733	59.890554	8.421817551
Os05g0215000|AK104	BURP domain containing protein.	84	76	63	645	553	677	74.3522733	624.803533	8.403287557
Os08g0436700|AK073	NAC-domain containing protein 19 (ANAC019) (ANAC) (Abscicic-acid-	83	107	120	963	619	1014	103.307253	865.212567	8.375138616
Os08g0508800|D1400	Lipoxygenase, chloroplast precursor (EC 1.13.11.12).	7	13	8	76	77	76	9.165576	76.6172067	8.359235324
Os05g0215000|AK104	BURP domain containing protein.	62	127	44	729	494	718	77.59348	647.113367	8.339790491
Os01g0602200|Os01g	Cytochrome P450 monooxygenase CYP72A5 (Fragment).	34	46	51	344	328	418	43.6219933	363.553833	8.334186624
Os09g0357400|Os09g	Disease resistance protein family protein.	16	6	5	14	182	32	9.14435267	76.1574367	8.328357342
Os01g0952000|AK060	Rhicadhesin receptor precursor (Germin-like protein).	9	7	6	7	14	171	7.72788167	64.1298733	8.298506124
osa-miR439j|Os10|NA|	miRNA	8	6	5	133	12	10	6.247948	51.7237777	8.278522431
POsControl0043|art|NA	NONE	8	18	5	230	12	10	10.2103123	84.0362013	8.230522103
Os07g0663700|AK070	Short-chain dehydrogenase/reductase SDR family protein.	190	356	85	1020	1885	2287	210.510117	1730.65267	8.221232756
Os06g0271400|CI4076	Hypothetical protein.	8	6	5	6	140	10	6.34116833	51.952259	8.192852842
Os07g0100300|AK109	Glycosyl transferase, group 1 domain containing protein.	8	6	5	6	135	10	6.16363	50.3556843	8.169809728
Os08g0277200|AK106	Cinnamoyl-CoA reductase (EC 1.2.1.44).	47	20	22	162	180	388	29.83108	242.947533	8.144107868
Os12g0218100|AK063	Non-protein coding transcript, unclassifiable transcript.	15	19	16	83	133	191	16.63523	135.24226	8.129870161
Os07g0663700|AK104	Short-chain dehydrogenase/reductase SDR family protein.	198	345	90	977	1855	2228	211.095743	1686.45737	7.989063825
Os11g0618200|CI4334	(No Hit)	9	7	6	98	15	69	7.61240667	60.78331	7.984769162
POsControl0019|genom	NONE	8	8	6	151	13	10	7.260028	57.9123133	7.976871898
Os04g0662600|CI2528	Flavanone-3-hydroxylase (Fragment).	33	32	25	207	253	255	29.95048	237.975767	7.945641161
Os06g0681200|AK107	Plastocyanin-like domain containing protein.	68	62	69	534	478	555	66.2302867	522.4921	7.889020663
Os02g0655200|AY339	Ethylene responsive element binding factor3 (OsERF3).	300	355	374	3139	2395	2569	342.7294	2701.158	7.881313946
Os02g0634700|AK104	Serine carboxypeptidase II-1 precursor (EC 3.4.16.6) (CP-MII.1) (Fragr	44	27	18	202	283	209	29.60113	231.4093	7.817583315
Os12g0217800|AK110	Non-protein coding transcript, unclassifiable transcript.	79	67	89	436	660	727	78.0702433	607.399167	7.780162335
Os05g0215000|AK119	BURP domain containing protein.	89	80	64	654	505	654	77.84576	604.360233	7.763560062
Os12g0555500|AK071	Probenazole-inducible protein PBZ1.	1379	1676	1344	7086	10951	15979	1466.47133	11338.7003	7.731961802

Os07g0520300|AK109(	Cytochrome P450 family protein.	1239	1131	1724	10934	9566	11083	1364.46767	10527.8727	7.715736271
Os12g0639400|AK110!	AAA ATPase, central region domain containing protein.	37	74	71	498	459	452	60.8674667	469.518967	7.713791823
Os12g0555500|D3817(	Probenazole-inducible protein PBZ1.	873	1034	893	4148	7624	9739	933.307133	7170.24733	7.682623519
Os04g0600300|AK107:	Alternative oxidase (OSJNBa0083N12.12 protein).	56	51	56	427	361	452	53.8835167	413.388367	7.671889146
Os07g0141200|CI2460	(No Hit)	7	6	5	32	49	59	6.10394	46.5547867	7.627005945
Os01g0128100|AK071!	Bifunctional nuclease (Fragment).	44	44	39	360	302	297	42.3147133	319.7224	7.555821009
Os03g0699000|U4393	Oleosin family protein.	38	14	28	212	169	221	26.66728	200.757367	7.528228101
Os12g0506700|CI2754	VMP3 protein.	8	6	5	6	125	10	6.250764	47.049977	7.52707621
Os01g0959100|AK063(	Abscisic stress ripening protein 1.	9	37	6	143	102	143	17.295025	129.2223	7.471645748
Os05g0215000|AK060	BURP domain containing protein.	83	77	61	575	483	572	73.92167	543.3994	7.351016285
Os05g0474800|AK119	WRKY transcription factor 70.	13	11	18	119	62	125	13.9828433	101.99149	7.294045107
Os02g0112900|AF324	Aminotransferase-like protein.	9	7	6	47	38	71	7.09986667	51.65261	7.275152116
Os01g0237500|AK110(	Hypothetical protein.	510	657	551	2943	4268	5220	572.6004	4143.747	7.236716915
Os05g0194900|AK071	Pyrophosphate-fructose-6-phosphate 1-phosphotransferase-like protei	177	211	169	1333	1416	1274	185.787033	1340.85333	7.217152399
Os12g0638200|AK060!	Peptide transporter.	104	135	81	920	674	713	106.721017	769.1288	7.206910354
Os05g0122700|CI4412	Small hydrophobic protein 2.	9	10	7	12	171	10	8.902805	64.1266333	7.202969551
Os04g0465900|Os04g(	Protein kinase domain containing protein.	152	170	187	1496	880	1291	169.724333	1222.46317	7.202639378
Os04g0600300|AB004	Alternative oxidase (OSJNBa0083N12.12 protein).	59	68	62	458	399	504	63.0212	453.870133	7.201864346
Os02g0570400|AB089	Terpene synthase, metal-binding domain containing protein.	43	39	30	188	306	305	37.2472133	266.388133	7.151894316
Os02g0634700|AK104	Serine carboxypeptidase II-1 precursor (EC 3.4.16.6) (CP-MII.1) (Fragr	51	37	36	212	460	207	41.17869	293.098567	7.117724402
Os08g0492100|AK100	Paraneoplastic encephalomyelitis antigen family protein.	23	18	12	17	346	15	17.7405867	125.993597	7.101997191
Os10g0136500|AK120(	Protein kinase domain containing protein.	45	42	45	293	283	357	43.96441	311.040133	7.074816501
Os07g0119800|Os07g(	Protein of unknown function DUF538 family protein.	939	1181	1437	10075	6756	8324	1185.2939	8384.76733	7.073998553
Os01g0627900|AB038!	Cytochrome P450 monooxygenase CYP72A5 (Fragment).	47	34	46	48	77	773	42.4909767	299.408543	7.04640295
Os02g0634700|AK106	Serine carboxypeptidase II-1 precursor (EC 3.4.16.6) (CP-MII.1) (Fragr	43	41	25	218	326	228	36.5787467	257.169367	7.030568024
Os10g0150700|AK061!	Protein of unknown function DUF1210 family protein.	11	10	5	20	136	31	8.91580467	62.39214	6.997925856
Os07g0141400|AK104	23 kDa polypeptide of photosystem II.	10	12	7	106	34	60	9.53440467	66.60358	6.985604485
Os04g0317400|AU182!	Pollen allergen/expansin, C-terminal domain containing protein.	12	16	7	6	227	10	11.6104593	81.055324	6.981233186
Os06g0578200|Os06g(	Hypothetical protein.	9	6	6	6	130	10	6.999152	48.8203553	6.97518147
Os02g0491600|Os02g(	Germin family protein.	56	53	31	358	301	323	46.9913367	327.545733	6.970342973
Os10g0101200|AK072!	Peptidase S10, serine carboxypeptidase family protein.	82	101	107	679	667	666	96.2331167	670.6537	6.9690531
Os05g0176800|AC144	HCO3-transporter domain containing protein.	11	8	7	10	26	147	8.78658333	60.9426003	6.935870067
Os03g0378500|Os03g(	P-type R2R3 Myb protein (Fragment).	25	17	14	44	339	10	18.9748	131.186197	6.913706425
Os03g0679700|AK062(	Thiamine biosynthesis protein thiC.	9	7	5	34	20	89	6.96563433	47.9763433	6.887577073
Os05g0560500|AK105	Hypothetical protein.	57	50	48	383	249	437	51.72977	356.244067	6.886635426
Os01g0939500|CI5163	Eukaryotic peptide chain release factor subunit 1-3 (eRF1-3) (Eukaryot	52	62	80	494	327	513	64.8027667	445.019567	6.867292703
Os04g0587200|Os04g(	Plant invertase/pectin methylesterase inhibitor domain containing prote	74	80	60	551	434	481	71.1950967	488.868133	6.86659835
Os04g0179700|AY347	Terpene synthase.	40	39	21	186	250	243	33.19514	226.558833	6.825060335
Os07g0629000|AK101(	P-type R2R3 Myb protein (Fragment).	7	6	5	47	26	48	5.88716367	40.17454	6.824090899
Os10g0574500|CI2797	Replication factor C conserved region domain containing protein.	153	193	185	1404	991	1213	176.920933	1202.64117	6.797619389
Os08g0120600|AK104!	Aldolase C-1.	66	87	56	506	419	483	69.39098	469.311033	6.763285853

Os04g0556500|AK1207	Cis-zeatin O-glucosyltransferase.	187	201	200	1511	915	1539	195.927267	1321.8824	6.746801619
Os10g0101200|AK104	Peptidase S10, serine carboxypeptidase family protein.	296	375	389	2422	2267	2448	353.389833	2379.302	6.732796973
Os07g0141400|AK065	23 kDa polypeptide of photosystem II.	16	12	7	97	63	73	11.5767407	77.6151133	6.704401141
Os04g0518400|AK067	Phenylalanine ammonia-lyase 2 (EC 4.3.1.5).	75	103	90	565	533	696	89.3418133	597.6115	6.68904601
Os01g0728100|AK104	Lipolytic enzyme, G-D-S-L family protein.	9	12	6	9	164	11	9.18913467	61.4634867	6.688713235
Os07g0550600|AK109	Transferase family protein.	8	8	5	33	92	13	6.91115167	46.1723267	6.680844075
Os10g0418100|AK109	Calcium-transporting ATPase 8, plasma membrane-type (EC 3.6.3.8) (	9	18	29	149	115	108	18.651464	124.312233	6.665012105
Os05g0241100|AK065	Leucyl-tRNA synthetase, cytoplasmic (EC 6.1.1.4) (Leucine--tRNA liga	31	19	25	52	430	21	25.1692833	167.72466	6.663863161
Os11g0644900|CI5212	(No Hit)	19	8	6	7	195	10	10.7019327	70.7621553	6.612091249
Os01g0678000|Os01g	Conserved hypothetical protein.	28	35	31	155	165	294	31.0983033	204.6107	6.579481131
Os08g0107600|AK119	DNA repair metallo-beta-lactamase domain containing protein.	10	11	5	7	157	10	8.86957333	57.964702	6.53523003
Os10g0117800|AK119	Guanine nucleotide binding protein (G-protein), alpha subunit family pr	19	10	31	122	259	9	19.9256187	130.193466	6.533973583
Os12g0170800|AK059	24 kDa protein SC24 (24 kDa seed coat protein).	149	261	296	1429	1137	2045	235.284	1537.27567	6.533702533
Os07g0216700|AK107	Cereal seed allergen, trypsin/alpha-amylase inhibitor family protein.	18	18	12	24	46	241	15.9387533	104.034247	6.52712571
osa-miR167a|Os12|NA	miRNA	12	22	6	7	26	227	13.237298	86.39558	6.526677876
POsControl0014|genon	NONE	13	13	9	6	212	10	11.6912137	76.279832	6.524543488
osa-miR167i|Os06|NA|	miRNA	23	15	9	21	236	46	15.599827	100.884593	6.467032829
Os01g0841100|AK119	Beta-Ig-H3/fasciclin domain containing protein.	27	26	22	46	64	370	24.76732	160.023147	6.461060247
Os11g0622800|Os11g	Zinc-containing alcohol dehydrogenase superfamily protein.	1700	2027	2470	16624	8141	15246	2065.78333	13336.915	6.456105432
Os09g0459600|AK108	Calmodulin-binding protein phosphatase.	8	15	12	57	111	52	11.375245	73.3577133	6.448890844
osa-miR166h|Os02|NA	miRNA	17	7	8	13	182	10	10.7042223	68.4452197	6.394226272
Os02g0570700|AK070	Cytochrome P450 family protein.	40	38	43	150	258	361	40.3625767	256.5038	6.354990716
Os03g0823200|AK072	Major facilitator superfamily protein.	9	7	6	115	13	11	7.36117333	46.7093467	6.345367043
Os03g0240600|AK109	U box domain containing protein.	14	11	10	29	73	121	11.73596	74.2780033	6.329094794
Os04g0474800|AK105	Glycoside hydrolase, family 1 protein.	8	6	5	26	12	80	6.23007367	39.42995	6.328970107
POsControl0034|rando	NONE	8	6	5	6	103	10	6.34740967	39.792322	6.269064719
POsControl0010|genon	NONE	18	17	15	11	288	10	16.4766	102.70006	6.233085689
Os04g0643500|Os04g	2OG-Fe(II) oxygenase superfamily protein.	121	127	172	994	699	927	140.2803	873.682533	6.228119938
POsControl0007|genon	NONE	10	6	5	6	117	10	7.17763333	44.2238067	6.161335445
Os07g0417600|CI5335	(No Hit)	34	34	31	280	158	169	32.88615	202.204567	6.148623863
Os12g0170800|AK104	24 kDa protein SC24 (24 kDa seed coat protein).	147	274	296	1311	1129	1954	238.659533	1464.56933	6.136647101
Os03g0664800|CI5431	GCN5-related N-acetyltransferase domain containing protein.	97	115	120	612	684	720	110.83518	671.755233	6.06084849
Os04g0100300|CI3469	Actin-binding FH2 domain containing protein.	10	7	7	7	14	123	7.94033467	48.087126	6.056057839
Os11g0116000|AK059	Plant lipid transfer protein/Par allergen family protein.	9	7	6	7	14	116	7.52314167	45.520866	6.050778786
Os01g0761500|AK069	TGF-beta receptor, type I/II extracellular region family protein.	51	36	25	180	232	264	37.3140033	225.234933	6.036203924
Os06g0215000|AK070	Conserved hypothetical protein.	8	6	6	6	103	10	6.69430933	39.9632487	5.969734393
Os09g0417600|AK068	DNA-binding WRKY domain containing protein.	218	224	168	1032	1268	1335	203.493067	1211.626	5.954138978
Os06g0671600|AK111	Non-protein coding transcript, putative npRNA.	8	6	8	34	49	45	7.20295833	42.74903	5.934926737
Os01g0932000|CI4076	(No Hit)	10	17	6	14	23	158	10.998907	65.1876067	5.926734963
Os02g0240300|AK104	Plant peroxidase family protein.	33	22	10	22	119	243	21.57308	127.82426	5.925174338
Os03g0655500|Os03g	PapD-like domain containing protein.	661	786	711	4742	3981	4023	719.353667	4248.68033	5.906246858

Os05g0165400|AK105	Hypothetical protein.	1050	1200	1420	1414	18636	1568	1223.358	7206.07733	5.890407659
Os12g0629300|Os12g	Thaumatin-like protein precursor.	82	79	68	388	455	487	76.0754	443.5005	5.82974917
Os06g0235300|AK066	Hypothetical protein.	9	7	6	8	106	11	7.12041133	41.498793	5.828145462
Os08g0553800|AK065	Isoflavone reductase family protein.	13	11	13	27	172	18	12.4143067	72.1280833	5.810077459
Os09g0467400|AK066	Protein of unknown function DUF250 domain containing protein.	324	319	332	1995	1718	1945	325.111	1885.887	5.800748052
Os02g0630300|AK108	2OG-Fe(II) oxygenase domain containing protein.	34	33	29	215	169	166	31.6557033	183.500267	5.79675216
Os06g0570100|AY660	Ent-kaurene oxidase (EC 1.14.13.78) (AtKO1) (Cytochrome P450 701	220	244	238	1680	939	1426	233.831033	1348.36287	5.766398272
Os09g0548300|BI3063	Multi antimicrobial extrusion protein MatE family protein.	25	31	7	28	60	278	21.152922	121.947377	5.765036938
Os09g0467400|AK061	Protein of unknown function DUF250 domain containing protein.	850	827	804	4956	4278	5066	827.0361	4766.91933	5.763858837
Os10g0100700|AK060	Vitamin B6 biosynthesis protein family protein.	13	6	13	53	68	64	10.7180767	61.61537	5.748733837
Os09g0381400|AK070	Peptidase C1A, papain family protein.	83	134	121	665	508	762	112.512997	645.057533	5.73318241
Os07g0191600|AK119	Conserved hypothetical protein.	8	6	19	63	79	44	10.813342	61.83998	5.718859165
Os02g0123800|CI4243	(No Hit)	7	7	5	30	26	51	6.25018067	35.5975533	5.695443897
Os04g0103100|AK070	Glycosyl transferase, family 43 protein.	54	43	31	45	663	24	42.9135233	244.37268	5.694537782
Os08g0207800|AK106	Peptidase aspartic family protein.	59	89	95	581	255	539	80.9736967	458.528733	5.662687418
Os04g0676600|AK072	Transcriptional factor B3 family protein.	8	6	5	7	28	77	6.617168	37.2863647	5.634791903
Os09g0321200|Os09g	Retinal pigment epithelial membrane protein family protein.	130	191	220	1232	631	1157	180.151433	1006.81893	5.588736735
osa-miR426|Os05|NA|	miRNA	19	24	12	7	291	10	18.4006533	102.732336	5.583080891
Os12g0207000|AK070	Transcription factor.	8	6	5	6	93	10	6.526736	36.4051433	5.577848305
Os11g0190700|AU172	PV72.	12	11	6	6	34	116	9.39359533	52.2304757	5.560222025
Os01g0686200|AK062	Zinc-containing alcohol dehydrogenase family protein.	23	16	14	7	34	257	17.84465	99.1459433	5.556059846
Os04g0368000|CI4478	(No Hit)	24	30	39	164	115	236	30.9077167	171.487433	5.548369528
Os11g0185100|AK107	Hypothetical protein.	8	6	5	6	92	10	6.51184267	36.010163	5.529949792
Os04g0414800|Os04g	TPR-like domain containing protein.	38	31	31	29	77	452	33.6168467	185.771647	5.526147307
Os02g0118800|Os02g	NBS-LRR protein (Fragment).	12	10	9	8	151	10	10.204561	56.348225	5.521866644
Os02g0634700|AK069	Serine carboxypeptidase II-1 precursor (EC 3.4.16.6) (CP-MII.1) (Frag	10	11	6	38	68	39	8.779725	48.43957	5.517208113
Os07g0674900|CI4273	(No Hit)	25	25	28	171	132	129	26.10954	143.933933	5.512695104
Os12g0638200|AK068	Peptide transporter.	123	119	75	644	618	489	105.863593	583.590367	5.512663497
Os10g0419600|Os10g	Chlorophyllase family protein.	57	43	40	331	210	227	46.4885067	255.814233	5.502741466
Os09g0269500|AK063	Conserved hypothetical protein.	28	27	19	38	339	27	24.5237633	134.543337	5.486243479
Os03g0383100|AK107	Conserved hypothetical protein.	7	6	5	6	12	83	6.08257867	33.3260537	5.478935085
Os04g0469000|AK062	Heavy metal transport/detoxification protein domain containing protein.	363	381	408	2147	1565	2594	384.046533	2102.01667	5.473338474
Os04g0369100|AU093	Protein kinase domain containing protein.	8	7	9	17	13	106	8.338877	45.5933967	5.467570353
Os12g0288000|AK069	Protein of unknown function DUF6 domain containing protein.	12	12	11	8	171	11	11.5879133	62.990374	5.43586858
Os01g0527900|CI4266	(No Hit)	9	13	6	7	54	87	9.03932033	49.0657233	5.428032366
Os02g0271000|AK072	Peptidase S8 and S53, subtilisin, kexin, sedolisin domain containing pr	9	10	8	34	78	33	8.968557	48.4263767	5.399572826
Os04g0417400|Os04g	U box domain containing protein.	168	148	148	925	659	919	154.643733	834.4178	5.395742731
Os01g0696800|AK061	Peptidase A1, pepsin family protein.	39	22	11	75	187	123	23.8772733	128.560743	5.3842305
Os10g0394100|AK066	Metallophosphoesterase domain containing protein.	16	8	8	10	37	126	10.8026033	57.491545	5.322008337
Os03g0812400|AK119	Calcium-binding EF-hand domain containing protein.	58	58	47	281	310	274	54.2526467	288.409833	5.31605094
Os01g0883800|BD187	Gibberellin 20-oxidase.	507	537	479	3168	2302	2623	507.6598	2697.82967	5.314247192

Os10g0531900|AK108(	Basic-leucine zipper (bZIP) transcription factor domain containing prote	333	391	375	2133	1752	1952	366.346933	1945.508	5.310561719
Os04g0371000|CI1626	Conserved hypothetical protein.	10	6	6	15	49	52	7.31829	38.84278	5.307630608
Os01g0297000|Os01g(	Protein of unknown function DUF6 domain containing protein.	42	67	55	89	707	71	54.7005667	288.816837	5.279960598
Os09g0334800|AK100!	Protein kinase domain containing protein.	120	114	134	733	566	642	122.870233	646.628	5.262690421
Os08g0173300|CI4437	(No Hit)	10	11	5	9	119	9	8.71921633	45.872596	5.261091622
Os09g0502500|AK109(	Zinc-containing alcohol dehydrogenase superfamily protein.	48	65	60	289	275	348	57.8703567	304.081067	5.254522076
Os01g0883800|CI2602	Gibberellin 20-oxidase.	502	523	463	2944	2250	2556	495.8588	2583.67367	5.210502802
Os01g0153200|Os01g(	Ribosomal protein S8 family protein.	35	20	12	18	320	10	22.3192433	115.861912	5.191121847
Os06g0191900|CI3396	Protein kinase family protein.	69	78	89	466	332	420	78.3848733	405.990933	5.179455118
Os12g0291400|AK099!	Ribulose-1,5-bisphosphate carboxylase/oxygenase small subunit (Frag	8	7	6	6	93	10	7.01623733	36.121288	5.14824204
Os01g0891000|AK070(	Glycoside hydrolase, family 20 protein.	29	43	42	241	151	190	37.75116	193.844433	5.134794092
Os05g0217800|AK119(	BURP domain containing protein.	12	7	6	13	104	11	8.27282367	42.3423433	5.1182456
Os12g0216000|AK105:	Conserved hypothetical protein.	113	92	112	128	101	1391	105.603653	540.076267	5.114181656
Os03g0727200|AB071(	Knotted1-type homeobox protein OSH3.	56	45	49	250	298	215	49.77749	254.2381	5.107491358
Os04g0474700|Os04g(	Hypothetical protein.	9	9	6	8	106	11	8.15150367	41.51809	5.093304462
Os04g0178400|AK071(	Cytochrome P450 CYP99A1 (EC 1.14.-.-) (Fragment).	1286	1190	1124	5464	6376	6485	1199.87133	6108.58	5.091029205
Os06g0189600|AK107:	Conserved hypothetical protein.	55	46	41	244	256	227	47.68217	242.412367	5.083920607
Os06g0537300|CI1556	(No Hit)	21	10	6	13	45	131	12.3790503	62.7084633	5.065692573
Os02g0817900|AK068'	Chloroplast carotenoid epsilon-ring hydroxylase.	437	615	709	3562	1832	3485	587.168167	2959.65933	5.04056504
Os02g0153400|CI2439	Protein kinase domain containing protein.	12	20	5	10	24	154	12.4389607	62.679696	5.038981767
Os06g0275000|AB041(	Hd1.	28	43	45	234	169	183	38.8074767	195.376867	5.034516115
Os01g0817400|CI3457	Conserved hypothetical protein.	8	8	5	6	91	10	7.09843733	35.6316737	5.019650381
Os07g0575600|AK110!	Resistance protein candidate (Fragment).	84	96	136	566	518	496	105.34997	526.942367	5.001827401
Os09g0297400|AK071(	Phosphate/phosphoenolpyruvate translocator.	19	17	6	7	31	173	14.0203373	70.103299	5.000115
Os01g0794400|AK122(	Thioredoxin domain 2 containing protein.	200	232	245	1269	990	1119	225.417733	1126.2217	4.996153955
osa-miR395k|Os08|NA	miRNA	25	28	10	26	61	231	21.1835917	105.77272	4.993143829
Os03g0781300|AK120(	Conserved hypothetical protein.	309	209	223	1389	939	1370	247.313967	1232.53783	4.983696837
Os09g0350900|AB059!	Protein kinase domain containing protein.	20	10	5	7	60	112	11.9446077	59.521331	4.983113105
Os07g0120500|Os07g(	Protein of unknown function DUF538 family protein.	1881	2342	2925	13137	8543	13894	2382.70567	11857.6413	4.976544732
Os12g0420200|AK111	38 kDa ribosome-associated protein.	66	52	47	277	330	215	55.14139	274.035767	4.96969276
Os05g0449600|AK063(	Exoglucanase precursor.	8	9	5	34	31	44	7.330418	36.2625767	4.946863421
Os05g0368000|Os05g(	Conserved hypothetical protein.	274	334	259	1194	1626	1464	289.008433	1428.17467	4.941636651
Os03g0663500|U7765(	Thaumatin, pathogenesis-related family protein.	47	57	39	202	277	226	47.59044	235.061533	4.93925951
Os10g0491000|AK100(	Plant Basic Secretory Protein family protein.	164	278	227	934	997	1363	223.023567	1097.83543	4.922508638
Os07g0657100|AK108:	Glyoxalase/bleomycin resistance protein/dioxygenase domain containi	8	7	8	43	37	35	7.80021367	38.35421	4.917071716
Os11g0461000|AK072!	Peptidase S10, serine carboxypeptidase family protein.	14	8	7	12	116	13	9.522631	46.7759633	4.912083996
Os10g0207500|AK064:	Conserved hypothetical protein.	35	34	28	409	50	12	32.0170367	156.784293	4.896902076
Os07g0168800|AY282	Zn-finger, A20-like domain containing protein.	7	26	5	8	175	10	13.0800433	63.963237	4.890139533
Os10g0411600|AK108(	Hypothetical protein.	9	9	6	12	13	89	7.78716467	37.9873367	4.878198715
Os08g0174500|AY062'	HAP3.	300	314	308	1691	1233	1562	307.630733	1495.57067	4.861577549
Os06g0317600|AK120(	Glycine rich family protein.	9	7	17	7	137	11	10.6730027	51.767561	4.850327749

Os03g0727200|AB007(	Knotted1-type homeobox protein OSH3.	55	50	51	252	290	213	52.14538	251.496267	4.82298272
Os11g0154500|AK106:	No apical meristem (NAM) protein domain containing protein.	93	85	110	433	398	556	95.89069	461.905367	4.816999092
Os05g0590100|AK072(	Ubiquitin domain containing protein.	16	11	5	9	136	10	10.710999	51.5651813	4.81422707
Os01g0698800|CI5550	Conserved hypothetical protein.	14	6	7	26	13	90	8.92599733	42.90674	4.806940715
Os02g0193200|CI5491	Conserved hypothetical protein.	26	21	15	23	124	150	20.5818667	98.61324	4.791268042
Os01g0887400|Os01g(	Peptide chain release factor 1 (RF-1).	33	18	16	12	300	9	22.39559	107.165542	4.785118037
Os05g0190500|AK109:	Acid phosphatase (Class B) family protein.	9	8	6	7	90	13	7.65179133	36.5530877	4.777062791
Os12g0413400|AK062(	Hypothetical protein.	50	39	44	212	213	208	44.22979	210.987933	4.770267581
Os01g0871400|AK068	Hypothetical protein.	15	13	5	6	139	9	10.7806357	51.332948	4.761588239
Os01g0785600|AK111:	Conserved hypothetical protein.	34	26	19	132	151	94	26.4245	125.750807	4.758871754
Os10g0205700|AK060	Conserved hypothetical protein.	12	21	7	19	51	120	13.3526073	63.4169133	4.749403
Os06g0521500|AK061	Haem peroxidase, plant/fungal/bacterial family protein.	118	231	176	942	582	964	174.949	829.259433	4.740006707
Os02g0228300|AK11(	Protein kinase domain containing protein.	3163	3503	3268	14616	15643	16827	3311.575	15695.1333	4.739476936
Os05g0410500|AK110	TGF-beta receptor, type I/II extracellular region family protein.	84	84	75	419	359	367	80.91135	381.603867	4.716320599
Os07g0614000|AK069	Strictosidine synthase family protein.	36	32	25	32	393	11	30.81048	145.199483	4.712665409
Os02g0570500|AK107	Cytochrome P450 family protein.	9	7	6	7	15	87	7.67818033	36.006674	4.689480116
Os11g0229500|AK111(	Disease resistance protein family protein.	10	14	15	96	36	48	12.8581467	60.18454	4.68065434
Os06g0649000|AK106:	WRKY transcription factor 28.	95	81	88	391	352	494	88.15589	412.214633	4.675973816
POsControl0041|art|NA	NONE	7	6	5	50	24	10	5.984761	27.9821987	4.675574959
Os12g0546800|AY100(	Alpha-expansin OsEXPA26.	8	6	5	6	76	10	6.56497767	30.6891737	4.674680589
Os08g0545800|AK109	Conserved hypothetical protein.	9	7	6	6	13	79	7.07138767	32.9584947	4.660824186
Os03g0337000|CI2110	(No Hit)	20	7	6	48	52	55	11.090324	51.52562	4.645997718
Os07g0222000|AK107	Alpha-amylase/trypsin inhibitor (RBI) (RATI).	14	6	5	8	44	67	8.56493033	39.6744413	4.632196619
Os01g0660200|AK100	Acidic class III chitinase OsChib3a precursor (Chitinase) (EC 3.2.1.14).	655	650	578	2551	2442	3720	627.786533	2904.55133	4.626654411
Os05g0434800|CI2397	Protein of unknown function DUF1218 family protein.	417	521	568	2468	2164	2326	502.161667	2319.58367	4.619197005
osa-miR439g|Os08|NA	miRNA	7	6	5	6	11	68	6.12375833	28.2701067	4.616463474
Os09g0417600|AF467	DNA-binding WRKY domain containing protein.	57	57	35	191	253	243	49.59165	228.875767	4.615207735
Os10g0552100|CI2609	Physical impedance induced protein.	16	18	17	8	215	11	16.9067033	78.0256227	4.615070196
Os12g0609200|AK067:	Hypothetical protein.	120	141	156	713	450	756	139.0534	639.682033	4.600261722
Os07g0693100|AK100	Pyruvate decarboxylase isozyme 3 (EC 4.1.1.1) (PDC).	38	27	11	24	315	10	25.3656233	116.641387	4.598404113
Os11g0625900|AK065	Protein kinase domain containing protein.	9	7	7	15	44	41	7.28440333	33.4775967	4.59579119
Os03g0692500|Os03g(	Glycosyl transferase, family 31 protein.	91	81	82	404	393	371	84.7005733	389.134933	4.594242022
Os10g0491000|AK059(	Plant Basic Secretory Protein family protein.	218	259	240	934	1024	1335	239.0195	1097.50597	4.591700538
Os09g0546200|AK071!	Auxin induced protein.	8	13	5	6	99	14	8.71890367	39.901038	4.576382482
Os01g0319000|AK107(	Pectinacetylesterase family protein.	129	141	116	594	653	522	129.0224	589.689	4.570438932
Os01g0883800|AB077(	Gibberellin 20-oxidase.	91	97	67	429	392	346	85.2441767	389.239167	4.566167237
Os03g0775600|AK106:	Conserved hypothetical protein.	11	7	6	7	37	61	7.70327733	35.130776	4.560497368
osa-miR395j|Os08|NA|(	miRNA	20	25	10	22	75	155	18.482252	83.92389	4.540782692
Os12g0117100|AK059(	Alpha/beta hydrolase fold domain containing protein.	34	51	48	236	159	209	44.42063	201.348067	4.532760266
Os11g0470200|Os11g(	Protein kinase domain containing protein.	53	70	63	295	193	357	62.18457	281.8062	4.531770502
Os01g0660200|D5571:	Acidic class III chitinase OsChib3a precursor (Chitinase) (EC 3.2.1.14).	668	660	548	2350	2440	3685	625.678933	2825.302	4.515577958

Os12g0420200|AK111!	38 kDa ribosome-associated protein.	79	56	49	269	329	229	61.04971	275.6608	4.515349868
Os11g0701200|D5570{	Glycoside hydrolase, family 18 protein.	58	48	40	180	201	277	48.6254	219.118333	4.506252562
Os02g0834700|AK067!	Conserved hypothetical protein YfcH family protein.	19	13	6	6	140	24	12.6030367	56.6730927	4.496780749
Os09g0417600|AK059!	DNA-binding WRKY domain containing protein.	125	106	79	341	598	454	103.41387	464.440267	4.491082934
Os01g0660200|AB006	Acidic class III chitinase OsChib3a precursor (Chitinase) (EC 3.2.1.14).	573	599	498	2113	1911	3468	556.604833	2497.48467	4.486997807
Os02g0539200|AK108	U box domain containing protein.	18	14	6	6	30	136	12.8256977	57.495799	4.482859373
Os07g0151900|CI4035	(No Hit)	13	11	6	78	50	10	10.2684453	45.7984083	4.460111229
POsControl0038|art|NA	NONE	8	6	6	6	73	10	6.644047	29.6001103	4.455132592
Os04g0513400|AK068	Beta-glucosidase.	75	126	108	471	388	517	102.889247	458.345867	4.454749952
Os04g0401900|AK105	Hypothetical protein.	199	207	237	1118	732	1004	213.9694	951.1021	4.445037935
Os03g0727200|AB028	Knotted1-type homeobox protein OSH3.	66	52	52	244	306	200	56.4172933	250.0336	4.431860964
Os12g0141000|CI4097	Hypothetical protein.	22	22	21	85	85	116	21.57913	95.38928	4.420441417
Os09g0439200|AK108	ZIM domain containing protein.	819	894	945	3786	3172	4783	885.931667	3913.946	4.417887008
Os02g0720100|AK063	Plastocyanin-like domain containing protein.	76	86	77	367	313	374	79.64161	351.157033	4.409215652
Os08g0442200|AK073	Strictosidine synthase family protein.	9	7	6	7	14	80	7.615756	33.5752237	4.408652754
POsControl0039|art|NA	NONE	8	6	5	6	53	28	6.61476167	29.137679	4.404947671
Os01g0660200|AB026!	Acidic class III chitinase OsChib3a precursor (Chitinase) (EC 3.2.1.14).	712	702	572	2410	2491	3847	662.1457	2916.10967	4.404030211
Os12g0626400|AY024	Squalene/phytoene synthase family protein.	537	412	530	2309	1636	2564	492.7278	2169.58533	4.403212754
Os01g0956800|Os01g	Disease resistance protein family protein.	18	7	9	22	27	100	11.2417507	49.43881	4.397785671
Os03g0580200|AK107	Hypothetical protein.	215	277	315	1341	948	1261	269.111033	1183.3336	4.397194665
Os07g0217600|AK065!	Cytochrome P450 family protein.	43	35	19	115	174	138	32.4147867	142.287233	4.389577966
Os01g0253000|AK071	Resistance protein candidate (Fragment).	8	6	5	11	12	62	6.46829133	28.3548067	4.383662579
Os06g0604500|AK070	Calcium-binding EF-hand domain containing protein.	24	20	22	20	25	246	22.1756133	97.2094667	4.383620205
Os04g0340300|AK071	Terpene synthase-like domain containing protein.	60	49	55	49	649	22	54.7862767	239.9345	4.379463519
Os12g0555000|AB127!	Bet v I allergen family protein.	772	738	717	2724	2930	4098	742.314567	3250.643	4.379064006
osa-miR395b|Os04|NA	miRNA	26	16	5	25	80	105	15.9593977	69.8772733	4.378440515
Os12g0578700|AK109	Zn-finger, C2H2 type domain containing protein.	8	7	19	25	103	21	11.4140267	49.89478	4.37135653
Os01g0674400|CI4391	(No Hit)	24	23	23	115	91	99	23.2841233	101.758153	4.370280636
Os03g0271500|AK109	Conserved hypothetical protein.	8	6	5	23	51	10	6.41403267	28.0292447	4.369987826
Os02g0534700|Os02g	Pectinesterase inhibitor domain containing protein.	10	8	10	67	15	36	8.99228967	39.2833467	4.368558857
Os09g0381400|AK121	Peptidase C1A, papain family protein.	239	280	310	1255	979	1383	276.2604	1205.6375	4.364134346
Os07g0686800|AK106	Serine/threonine protein kinase-like.	27	29	24	123	100	126	26.7116767	116.31795	4.35457315
Os01g0154000|AK108	Hypothetical protein.	12	14	8	122	12	10	11.0050953	47.918201	4.35418318
Os07g0192800|CI2591	AAA ATPase, central region domain containing protein.	8	7	6	6	74	11	6.929866	30.159389	4.352088338
Os11g0702400|AY305	Zn-finger, C2H2 type domain containing protein.	47	59	40	212	174	251	48.92224	212.691367	4.347539415
Os01g0628900|AK060	Cytochrome P450 family protein.	11	11	10	47	63	32	10.9619333	47.53565	4.336429401
Os09g0483300|CI4135	Calcium-binding EF-hand domain containing protein.	7	18	13	21	134	12	12.839902	55.6027133	4.330462439
Os07g0526400|CI4267	Chalcone synthase (EC 2.3.1.74) (Naringenin-chalcone synthase).	55	155	40	471	404	206	83.3489767	360.4782	4.324926525
Os03g0679700|AK064!	Thiamine biosynthesis protein thiC.	13	10	6	37	13	73	9.50655067	40.84031	4.296017707
Os03g0137700|AK069	Mg2+ transporter protein, CorA-like family protein.	68	67	62	318	235	291	65.5138867	281.3494	4.294500209
Os06g0281800|AK064	BURP domain containing protein.	15	21	24	126	89	39	19.7431067	84.72145	4.291191423

Os11g0191300|AK070	MEI1 protein.	8	6	5	6	12	65	6.46811767	27.7170117	4.285174311
Os11g0133800|Os11g	Zn-finger, DHHC type domain containing protein.	144	188	189	928	559	731	173.2539	739.3738	4.267573775
Os09g0552200|CB620	Conserved hypothetical protein.	25	25	14	21	234	17	21.2288167	90.5785667	4.266774173
Os04g0220300|AK058	Conserved hypothetical protein.	9	6	5	62	17	10	6.89554367	29.419171	4.266403408
Os07g0569800|AK108	Protein kinase family protein.	137	143	147	605	574	643	142.4071	607.249733	4.264181585
Os04g0593800|AK102	Zn-finger, cysteine-rich C6HC domain containing protein.	24	20	18	16	56	191	20.62993	87.85263	4.258503543
Os02g0204200|CI1323	(No Hit)	15	8	7	45	53	32	10.083318	42.93756	4.258276889
Os06g0649000|AK119	WRKY transcription factor 28.	108	126	115	482	401	601	116.1458	494.414167	4.256840684
Os06g0686800|AK111	Hypothetical protein.	12	13	6	8	21	103	10.366222	43.975062	4.242149358
Os03g0306800|AK103	CP12 (Fragment).	186	200	235	982	744	904	206.9426	876.7593	4.236726996
Os07g0417200|AK105	Delta-12 oleate desaturase.	338	699	431	1254	2293	2671	489.6175	2072.777	4.233461835
Os04g0179100|CI0279	Glucose/ribitol dehydrogenase family protein.	729	505	575	2539	2252	2866	603.226633	2552.27333	4.231035555
Os08g0501900|AK063	Conserved hypothetical protein.	16	20	21	20	27	193	18.9076567	79.9761433	4.22982841
Os02g0621100|AK100	Pre-SET domain containing protein.	24	29	23	126	52	141	25.15913	106.31518	4.225709713
Os02g0320800|AK111	Iron/ascorbate-dependent oxidoreductase.	8	7	6	6	70	11	6.85986433	28.9800223	4.224576599
Os12g0626400|AK063	Squalene/phytoene synthase family protein.	621	500	629	2672	1923	2792	583.089333	2462.35067	4.222938966
Os06g0570100|AK066	Ent-kaurene oxidase (EC 1.14.13.78) (AtKO1) (Cytochrome P450 701	609	684	691	2926	1969	3430	661.292167	2774.79133	4.196014218
Os12g0135800|AK061	Esterase/lipase/thioesterase domain containing protein.	1266	1537	1261	4836	6032	6166	1354.49933	5678.016	4.191966626
Os03g0693900|CI2435	Oxalate oxidase 1 (EC 1.2.3.4) (Germin).	10	7	6	7	78	13	7.75786067	32.5037597	4.189783893
Os08g0138400|Os08g	Plastocyanin-like domain containing protein.	9	15	9	51	45	40	10.889781	45.4764933	4.176070514
Os01g0921500|AK105	CBS domain containing protein.	9	7	6	8	69	11	7.05183533	29.4333197	4.173852377
Os12g0160700|CI3642	(No Hit)	9	15	9	109	23	9	11.252765	46.9589697	4.173104981
Os07g0468500|AK073	Leucine-rich repeat, plant specific containing protein.	13	7	5	6	89	10	8.350214	34.7721133	4.164218226
Os12g0626400|AK073	Squalene/phytoene synthase family protein.	555	434	531	2290	1669	2369	506.5481	2109.175	4.163819783
Os05g0156900|AK107	Pyrophosphate-energized vacuolar membrane proton pump (EC 3.6.1.	8	6	9	12	12	70	7.522399	31.3028833	4.161289947
Os03g0381100|Os03g	tRNA pseudouridine synthase family protein.	29	29	20	40	266	18	25.9844667	107.90755	4.152771399
Os12g0165400|Os12g	Hypothetical protein.	76	64	45	249	249	270	61.7096233	255.838833	4.145849861
Os10g0157400|CI4889	(No Hit)	13	7	7	43	26	41	8.80534867	36.4716567	4.141988926
Os12g0626400|AY452	Squalene/phytoene synthase family protein.	693	526	699	2877	2010	3008	639.148333	2631.284	4.116859675
Os05g0546300|AK108	Conserved hypothetical protein.	105	70	97	368	406	348	90.8148533	373.862033	4.116749844
Os01g0940700|AK060	Beta-1,3-glucanase (Fragment).	604	864	812	2191	3652	3539	759.860067	3127.196	4.115489334
PAtControl0002|X6427	NONE	7	6	5	6	11	56	5.93633367	24.40794	4.111618614
Os01g0233800|AK069	Protein kinase PVPK-1 (EC 2.7.1.-).	16	8	7	23	94	13	10.5229443	43.2656333	4.111552049
Os03g0131200|AK062	Catalase isozyme 2 (EC 1.11.1.6).	38	46	65	184	190	238	49.69178	203.914667	4.1035895
Os05g0366600|AK071	Glycoside hydrolase, family 1 protein.	952	1311	1362	5355	4456	5050	1208.5666	4954.009	4.099078197
Os06g0625000|Os06g	Conserved hypothetical protein.	22	31	16	18	237	27	22.9890833	94.0999933	4.093246867
Os03g0269700|AK060	Protein of unknown function DUF604 family protein.	8	6	6	6	66	10	6.709094	27.450142	4.091482695
POsControl0029|rando	NONE	12	6	8	7	90	10	8.756562	35.8008787	4.088462877
Os03g0131200|AK066	Catalase isozyme 2 (EC 1.11.1.6).	45	43	63	165	160	288	50.2439567	204.5599	4.071333421
Os08g0411700|Os08g	Conserved hypothetical protein.	32	24	20	36	240	32	25.3293567	102.765793	4.057181344
Os01g0823600|CI4566	Conserved hypothetical protein.	8	6	5	6	15	57	6.43874	26.0965147	4.053046818

Os04g0667200|CI4398	2OG-Fe(II) oxygenase domain containing protein.	9	7	6	7	67	11	6.96812467	28.223517	4.050374864
Os03g0131200|AB020	Catalase isozyme 2 (EC 1.11.1.6).	46	44	64	185	188	255	51.5988233	208.9922	4.050328796
Os11g0209000|CI4628	Glycine rich family protein.	15	11	11	6	22	125	12.53305	50.749655	4.04926614
Os01g0695800|AK108	Multidrug resistance protein 1 homolog.	24	22	15	67	99	82	20.3418367	82.36553	4.049070463
Os03g0339300|AK061	Peroxidase (EC 1.11.1.7).	10	6	12	6	12	98	9.59560733	38.7899473	4.042469224
Os11g0454100|CI1270	(No Hit)	21	9	7	16	32	103	12.4324907	50.2077933	4.038434026
Os01g0597800|AK063	UDP-glucuronosyl/UDP-glucosyltransferase family protein.	8	14	6	68	13	32	9.34717733	37.7430933	4.037913478
Os01g0862800|AK071	No apical meristem (NAM) protein domain containing protein.	10	8	7	7	81	13	8.31035567	33.533508	4.035147152
Os01g0713200|AF030	Beta-1,3-glucanase precursor.	7972	13531	9118	44038	36755	42530	10206.9447	41107.8767	4.027441904
PAtControl0003|AY056	NONE	14	15	8	6	134	9	12.409195	49.905071	4.021620339
Os01g0508500|AK120	Hypothetical protein.	29	38	27	119	173	82	30.9620433	124.4389	4.019079059
Os02g0535000|AK067	Conserved hypothetical protein.	468	556	627	2570	1479	2577	550.4207	2208.58233	4.012535018
Os08g0503600|AK119	Conserved hypothetical protein.	16	27	28	45	44	194	23.4629233	94.0985367	4.01052057
Os10g0572800|Os10g	Enoyl-CoA hydratase/isomerase domain containing protein.	8	6	5	6	61	10	6.420817	25.731086	4.007447339
Os02g0569400|AK060	Cytochrome P450 family protein.	9	7	6	18	31	35	7.02406433	28.0568133	3.994384448
Os11g0136300|CI3523	Protein of unknown function DUF6 domain containing protein.	110	105	65	372	359	383	93.1086533	371.5279	3.990261772
Os07g0218700|AK108	Cytochrome P450 family protein.	11	6	5	67	12	10	7.43532433	29.6411067	3.986525044
Os08g0452200|AK100	Conserved hypothetical protein.	43	37	17	32	343	9	32.0977367	127.935866	3.985822022
Os12g0615100|AK067	Protein kinase domain containing protein.	38	55	74	104	465	94	55.4603933	220.787623	3.980996348
Os07g0141400|D4971	23 kDa polypeptide of photosystem II.	88	57	48	295	232	244	64.5212	256.827933	3.980520098
Os11g0417200|AU092	Hypothetical protein.	22	11	6	8	136	10	12.876004	51.2184773	3.977823969
Os01g0713200|AB027	Beta-1,3-glucanase precursor.	8087	13042	9053	40888	37371	41799	10060.8747	40019.4733	3.977733016
Os05g0177500|CI2597	UDP-glucuronosyl/UDP-glucosyltransferase family protein.	37	31	25	86	186	99	31.0719067	123.403083	3.971532377
Os01g0713200|AK104	Beta-1,3-glucanase precursor.	5202	8194	5675	24973	23979	26721	6357.026	25224.4567	3.967964999
Os04g0319800|CI4417	UDP-glucuronosyl/UDP-glucosyltransferase family protein.	34	27	30	24	303	33	30.29516	119.922647	3.958475435
Os06g0641200|Os06g	Elicitor-inducible cytochrome P450.	217	134	155	697	525	779	168.7051	666.870233	3.952875363
Os04g0629600|AK070	Zn-finger, CCHC type domain containing protein.	89	115	97	131	970	86	100.291103	395.67683	3.945283448
Os03g0127500|C98212	Basic-leucine zipper (bZIP) transcription factor domain containing prote	213	213	248	1008	645	1007	224.7329	886.486533	3.944622854
Os01g0713200|AB027	Beta-1,3-glucanase precursor.	8096	13628	9326	41651	37851	42931	10349.967	40810.9833	3.943102749
Os02g0148000|AK120	Zinc finger protein.	24	17	7	74	61	53	15.93329	62.7200167	3.936413425
Os08g0543000|Os08g	NBS-LRR type resistance protein (Fragment).	19	17	23	96	78	58	19.6581533	77.3692	3.935730823
Os01g0703000|AK109	Conserved hypothetical protein.	44	31	26	38	73	289	33.8150333	133.07745	3.935452279
Os03g0131200|AY339	Catalase isozyme 2 (EC 1.11.1.6).	51	60	68	221	198	284	59.4955567	234.061933	3.934107796
Os05g0384300|AK107	Peptidase aspartic family protein.	23	31	14	97	97	73	22.65461	89.0419867	3.930413574
osa-miR394|Os02|NA|	miRNA	16	11	14	11	137	12	13.5430933	53.2164	3.929412483
Os01g0209800|AK099	Cationic amino acid transporter (Fragment).	1484	1702	1888	7163	5485	7286	1691.00767	6644.65433	3.929405209
Os09g0417600|AY323	DNA-binding WRKY domain containing protein.	132	116	88	335	549	433	111.798157	439.2196	3.928683738
Os02g0221900|AK122	Cytochrome P450 family protein.	27	28	28	26	61	237	27.51814	108.04719	3.926398732
Os06g0275000|AB041	Hd1.	145	126	156	661	479	534	142.062333	557.6249	3.925212876
Os04g0513400|AK066	Beta-glucosidase.	38	38	43	190	132	142	39.4036967	154.6178	3.923941485
Os06g0148200|AK073	Lipolytic enzyme, G-D-S-L family protein.	27	7	5	37	109	10	13.264483	52.0112067	3.921088117

Os01g0892500|CI4062	Pectinacetylesterase family protein.	17	26	21	51	40	159	21.2536067	83.3276767	3.920636999
Os06g0556200|AK071	Amino acid/polyamine transporter II family protein.	8	7	10	31	41	24	8.21874633	32.2126433	3.919410823
Os10g0116600|CI2724	Protein kinase domain containing protein.	8	7	7	6	69	10	7.17569233	28.0757327	3.912616562
Os11g0192400|AK100	Pectinesterase family protein.	10	6	5	6	20	56	7.01277433	27.4324293	3.911779851
Os10g0459400|CI2711	Conserved hypothetical protein.	30	21	19	18	243	11	23.2353333	90.6574867	3.901708031
Os04g0369000|AK119	Major sperm protein domain containing protein.	73	105	102	335	280	477	93.3495533	364.017333	3.899508035
Os03g0399900|AK099	GOS9 protein.	8	11	10	42	12	57	9.52779867	37.02301	3.885788449
Os01g0233300|Os01g	Cyclin.	22	20	11	7	188	12	17.7461967	68.860599	3.880301807
Os06g0313200|CB669	SAM dependent carboxyl methyltransferase family protein.	16	7	5	29	39	41	9.34905233	36.26821	3.879346131
Os10g0503300|CI2151	Benzoyl coenzyme A: benzyl alcohol benzoyl transferase.	224	196	219	757	835	881	212.6467	824.385867	3.876786551
Os10g0558400|Os10g	2OG-Fe(II) oxygenase domain containing protein.	76	94	59	308	309	271	76.3384167	295.768367	3.874436746
Os06g0476200|AK065	Phosphoglucomutase precursor (EC 5.4.2.2).	8	6	6	55	13	10	6.67608467	25.8537267	3.872588195
Os04g0178300|AK121	Copalyl diphosphate synthetase (Fragment).	276	279	278	1023	1094	1111	277.946733	1075.933	3.8710043
Os02g0571900|AK101	Cytochrome P450 family protein.	54	51	29	99	254	166	44.78244	172.957547	3.862173358
Os06g0176800|Os06g	2OG-Fe(II) oxygenase domain containing protein.	144	197	186	795	458	780	175.554533	677.7704	3.860739949
Os02g0741200|AK071	Plant peroxidase family protein.	7	6	5	50	12	10	6.13727	23.6828193	3.858852443
Os08g0189700|CI4113	Oxalate oxidase-like protein or germin-like protein (Germin-like 8) (Ger	52	64	75	233	192	309	63.6538133	244.919667	3.847682548
Os01g0825500|AK060	Nodulin-like domain containing protein.	8	6	6	6	13	60	6.83648267	26.275333	3.843399345
Os07g0489900|Os07g	Conserved hypothetical protein.	21	16	12	66	72	46	15.9799433	61.39895	3.842250796
Os03g0279500|CI1323	(No Hit)	16	6	11	52	45	31	11.084602	42.5558767	3.839188513
Os06g0162800|AF141	MADS box protein.	21	12	7	18	124	11	13.3325627	51.1348167	3.835332932
Os05g0530400|AB050	Heat shock factor protein 1 (HSF 1) (Heat shock transcription factor 1)	45	50	54	287	128	153	49.4241467	189.2418	3.828934089
Os12g0146500|AK107	Protein of unknown function DUF668 family protein.	138	141	139	636	470	496	139.517267	533.9145	3.826870414
Os01g0713200|AK060	Beta-1,3-glucanase precursor.	5306	8824	6010	26172	23839	26962	6713.27333	25657.5033	3.821906551
Os02g0731700|AK120	CONSTANS-like 1 protein.	41	36	19	27	106	235	32.15175	122.668533	3.815298804
Os07g0525900|Os07g	Chalcone synthase.	39	42	16	37	90	243	32.3567333	123.380163	3.813121741
Os03g0132900|AF001	Chitinase (EC 3.2.1.14) (Fragment).	60	66	63	183	246	291	63.0664133	240.333933	3.810807062
Os06g0142500|Os06g	Calycin family protein.	43	43	26	44	356	26	37.2203967	141.770983	3.808959496
Os06g0248600|AK111	Hypothetical protein.	10	6	6	22	36	24	7.20760967	27.4439433	3.807634514
Os11g0701200|AK073	Glycoside hydrolase, family 18 protein.	58	48	50	156	204	230	51.7598733	196.707033	3.800377023
Os10g0569800|CB617	Hypothetical protein.	65	51	35	121	283	171	50.50492	191.842267	3.798486695
Os03g0132900|AK059	Chitinase (EC 3.2.1.14) (Fragment).	87	102	88	268	317	464	92.5887767	349.556867	3.775369751
Os01g0218500|AK100	SP3D.	14	6	5	9	12	74	8.43591733	31.8435577	3.774759331
Os12g0555000|AK061	Bet v I allergen family protein.	309	285	258	863	1052	1294	284.104033	1069.39677	3.764102727
Os07g0575700|AK105	Receptor like protein kinase.	127	144	192	624	499	619	154.381267	580.456767	3.759891204
Os01g0713200|AK060	Beta-1,3-glucanase precursor.	8831	13763	9683	42571	36210	42462	10758.815	40414.1233	3.756373107
Os04g0204200|CI5359	(No Hit)	10	12	8	20	79	11	9.74070267	36.5192367	3.74913781
Os10g0158000|AK065	Non-protein coding transcript, putative npRNA.	7	6	5	15	43	10	6.05633233	22.680903	3.744989831
Os07g0617500|Os07g	Plant disease resistance response protein family protein.	11	7	9	10	14	74	8.74259233	32.6995497	3.740257857
Os09g0417800|AK067	DNA-binding WRKY domain containing protein.	40	47	38	126	185	158	41.7553767	156.020533	3.736537562
Os01g0971600|AK070	Sn-glycerol-3-phosphate dehydrogenase (Fragment).	65	39	58	220	140	244	53.8377833	201.131333	3.735876941

Os01g0913400|AK064	TPR-like domain containing protein.	37	31	21	20	299	15	29.9103867	111.419523	3.725111433
Os06g0691700|AK069	Conserved hypothetical protein.	13	12	7	6	104	9	10.6228457	39.5420553	3.722359956
Os01g0860500|D5571	Chitinase (EC 3.2.1.14).	1084	1164	954	4195	3096	4626	1067.27077	3972.31233	3.721934918
Os12g0527500|AK109	Hypothetical protein.	19	15	10	29	121	18	14.9606567	55.6741933	3.721373638
POsControl0040|art|NA	NONE	9	7	6	6	20	52	7.089411	26.374678	3.720291855
Os02g0293300|CI5275	X1 (Fragment).	9	7	11	6	81	11	8.726881	32.4597363	3.71951174
Os03g0860700|Os03g	Myosin XI (Fragment).	26	15	12	27	56	115	17.75385	65.9649467	3.71552912
Os01g0940700|AK070	Beta-1,3-glucanase (Fragment).	189	248	222	604	994	852	219.901467	816.790133	3.714346001
Os03g0189300|AK069	Conserved hypothetical protein.	17	7	15	9	124	11	12.9082117	47.918271	3.712231581
Os11g0226700|Os11g	Disease resistance protein family protein.	16	8	5	6	67	37	9.87885967	36.642495	3.709182662
Os04g0403600|AU101	Protein phosphatase 2C-like domain containing protein.	200	204	260	903	637	922	221.563767	820.8891	3.704978988
Os09g0381400|AK071	Peptidase C1A, papain family protein.	270	334	293	1086	993	1229	298.574033	1102.35867	3.692078157
POsControl0042|art|NA	NONE	7	6	5	6	12	50	6.06702367	22.3956637	3.691375689
osa-miR395o|Os04|NA	miRNA	14	7	6	9	80	10	8.94704833	33.0153363	3.690081366
Os05g0322900|AK105	WRKY transcription factor 45.	3652	4476	4064	15679	13175	16093	4063.978	14982.2733	3.686602962
Os05g0322900|AK066	WRKY transcription factor 45.	1515	1912	1687	6147	5899	6799	1704.57833	6281.798	3.685250409
osa-miR172b|Os01|NA	miRNA	8	6	6	6	58	11	6.810127	25.0759243	3.682152232
Os11g0259700|CI5595	(No Hit)	34	23	16	27	226	14	24.1585833	88.84719	3.677665564
Os11g0608300|CI3927	Barley stem rust resistance protein.	30	28	16	85	112	76	24.7356733	90.88983	3.674443334
Os02g0277700|AK120	Leucine-rich repeat, plant specific containing protein.	13	15	15	47	39	69	14.0650833	51.6515033	3.672321174
Os01g0860500|AK104	Chitinase (EC 3.2.1.14).	1428	1599	1259	5896	4022	5816	1428.58033	5244.53833	3.67115395
Os02g0629900|AK108	Conserved hypothetical protein.	12	7	6	29	39	21	8.13222133	29.8012367	3.664587503
Os01g0940700|AK104	Beta-1,3-glucanase (Fragment).	195	248	252	611	1053	878	231.500033	847.1762	3.659507896
POsControl0040|art|NA	NONE	13	6	5	23	55	9	7.94646433	29.0720227	3.658485264
Os04g0429300|AK107	Basic helix-loop-helix dimerisation region bHLH domain containing prot	17	16	40	108	61	95	24.0431467	87.87852	3.655034061
Os03g0111000|AK062	Hypothetical protein.	83	131	107	489	299	384	106.838413	390.3931	3.654051832
Os05g0322900|AK103	WRKY transcription factor 45.	3201	3890	3519	12707	12254	13775	3536.48067	12911.9233	3.651065721
Os02g0157400|CI3276	(No Hit)	26	15	15	19	131	57	18.8722633	68.8910167	3.650384453
Os02g0626600|CI1982	Phenylalanine ammonia-lyase.	26	26	18	68	72	115	23.2290633	84.7803667	3.649753994
POsControl0012|genon	NONE	9	12	6	8	46	45	9.09306	33.1866223	3.649664946
Os12g0420400|AK099	Photosystem I reaction center subunit XI, chloroplast precursor (PSI- L	9	7	6	7	64	11	7.47460833	27.2770123	3.649289851
Os05g0505900|AK106	Hypothetical protein.	13	6	9	8	84	10	9.26028367	33.7862687	3.648513359
Os06g0178600|Os06g	2OG-Fe(II) oxygenase domain containing protein.	158	203	245	828	578	801	201.714	735.705667	3.647271219
Os06g0587300|AK121	Conserved hypothetical protein.	304	350	416	1447	1027	1423	356.564267	1299.25767	3.643824657
Os05g0457300|Os05g	Hypothetical protein.	7	14	6	7	82	10	9.058618	32.9643867	3.639008364
Os01g0860500|AB003	Chitinase (EC 3.2.1.14).	1502	1584	1283	5825	4168	5874	1456.26033	5288.98767	3.63189709
Os07g0543500|Os07g	Conserved hypothetical protein.	81	81	88	362	231	315	83.43178	302.789467	3.629186225
Os01g0143400|Os01g	Protein of unknown function DUF594 family protein.	18	34	15	212	23	10	22.5330267	81.7641963	3.628637979
Os09g0134500|AK065	Trithorax-like protein 1.	9	7	7	7	15	65	7.95017633	28.823607	3.625530528
Os02g0618700|AK070	Lung seven transmembrane receptor family protein.	65	56	40	38	519	25	53.51632	193.956617	3.624251755
Os01g0940700|AB027	Beta-1,3-glucanase (Fragment).	193	284	231	631	1036	902	236.254567	856.1866	3.624000213

Os05g0366800|Os05g(	Glycoside hydrolase, family 1 protein.	623	910	823	3103	2598	2837	785.429633	2846.33767	3.623924469
Os04g0464100|AK120!	Heavy metal transport/detoxification protein domain containing protein.	29	25	17	74	106	77	23.6288533	85.6118267	3.623190066
Os06g0673800|AK066(	Hypothetical protein.	77	99	91	313	329	325	88.9788233	322.2957	3.622161857
Os02g0577300|Os02g(	Glycosyl transferase, family 31 protein.	50	32	34	135	173	109	38.4228767	139.018067	3.618106678
Os06g0553200|AK060ŧ	Hypothetical protein.	7	6	5	6	50	10	6.048413	21.8465547	3.611948236
Os10g0189600|AK103	Aminotransferase, class I and II domain containing protein.	713	530	700	2369	1711	2938	647.8501	2339.49367	3.611165093
Os01g0700900|AK067(	Cytochrome P450 family protein.	2196	2290	3148	10337	6519	10695	2544.97833	9183.88067	3.608628233
Os04g0400600|AU162	Heavy metal transport/detoxification protein domain containing protein.	10	40	15	59	132	42	21.5697483	77.83707	3.608622076
Os09g0444800|AK110ŧ	Protein of unknown function DUF716 family protein.	35	29	25	246	48	25	29.48713	106.236377	3.602804907
osa-miR169n|Os11|NA	miRNA	8	6	5	6	54	10	6.50793933	23.427002	3.599757281
Os10g0150600|AB071	Protein of unknown function DUF1210 family protein.	32	28	11	35	67	155	23.9138	85.91095	3.592526073
Os10g0375000|CI4256	(No Hit)	36	50	54	186	137	180	46.6668733	167.473867	3.588709821
Os12g0222000|CI4027	Hypothetical protein.	95	91	76	334	292	314	87.3722733	313.3608	3.586501622
Os04g0234600|AK119;	Sedoheptulose-1,7-bisphosphatase, chloroplast precursor (EC 3.1.3.37	7	6	5	6	51	10	6.13782833	22.0102067	3.585992548
Os10g0375000|CI4256	(No Hit)	38	58	57	211	136	199	50.87173	181.9163	3.575980215
Os02g0124600|C9938(	Plant-specific FAD-dependent oxidoreductase family protein.	2483	1644	969	7218	5015	5985	1698.40587	6072.79633	3.575586055
Os07g0538300|CI4270	Serine/threonine kinase receptor-like protein.	33	31	40	91	190	91	34.67756	123.986517	3.575410631
Os02g0204500|AK120(	Agenet domain containing protein.	43	51	29	42	91	305	40.9191067	146.13871	3.571405192
osa-miR166f|Os10|NA|	miRNA	17	13	6	7	98	23	12.0048417	42.7529107	3.561305668
Os08g0413500|CI2708	Protein of unknown function DUF1262 family protein.	70	106	89	339	271	334	88.4308233	314.719967	3.558939686
osa-miR171g|Os07|NA	miRNA	30	21	19	16	57	175	23.2215833	82.6134467	3.557614719
Os05g0148500|Os05g(	Conserved hypothetical protein.	8	6	5	6	52	10	6.35705367	22.5885777	3.553309261
Os08g0355600|AU055	Hypothetical protein.	7	6	5	6	12	47	6.08837667	21.6162897	3.550419241
Os07g0677200|AK122(	Peroxidase.	1147	1295	1208	5191	3457	4310	1216.804	4319.45133	3.549833279
Os01g0727800|AK108	Protease-associated PA domain containing protein.	2447	2323	2651	8935	8108	9291	2473.64467	8777.976	3.548600217
Os07g0160100|AK100;	YABBY2.	11	6	16	6	79	31	10.9626803	38.8484383	3.543698909
Os01g0773200|AK069!	Hypothetical protein.	14	7	10	23	78	10	10.4829887	37.14476	3.543336846
Os01g0592500|AK111;	Protein of unknown function DUF1070 family protein.	51	6	6	78	57	89	21.0788167	74.64995	3.541467777
Os06g0641100|Os06g(	Cytochrome P450 family protein.	101	62	69	278	238	303	77.2426033	272.920333	3.53328761
Os02g0212400|Os02g(	Conserved hypothetical protein.	165	192	218	730	511	788	191.408767	676.2191	3.532853337
osa-miR172d|Os02|NA	miRNA	7	6	5	43	11	10	6.05083533	21.3620357	3.530427534
Os09g0467500|Os09g(	Conserved hypothetical protein.	468	489	460	1729	1662	1608	472.324833	1666.398	3.528076193
Os09g0271100|AK100(	Hypothetical protein.	14	19	13	39	78	44	15.2496567	53.79908	3.52788795
Os04g0121800|AK058ŧ	Non-protein coding transcript, uncharacterized transcript.	124	146	226	542	471	731	165.138	581.4231	3.520831668
Os09g0478300|CI5193	(No Hit)	284	271	259	1012	780	1070	271.435733	954.0537	3.514841942
Os01g0693300|AK101;	Lipid phosphate phosphatase 2 (EC 3.1.3.-) (AtLPP2) (Phosphatidic ac	119	99	74	378	333	315	97.42813	342.2159	3.512495826
Os04g0179200|AK103	Stem secoisolariciresinol dehydrogenase (Fragment).	29	18	9	21	169	10	18.9948737	66.7191763	3.512483289
Os08g0298600|Os08g(	Conserved hypothetical protein.	69	42	26	69	135	275	45.5107967	159.83681	3.512063548
Os05g0527900|AK110ŧ	UDP-glucuronosyl/UDP-glucosyltransferase family protein.	33	29	21	42	203	43	27.2640933	95.7015867	3.510169419
Os04g0368000|CI4338	(No Hit)	32	35	25	94	105	125	30.71166	107.755793	3.508628102
Os04g0472100|AK072!	Hypothetical protein.	8	6	7	7	57	10	7.01809467	24.606953	3.5062156

Os02g0821100|AK110	Conserved hypothetical protein.	9	10	12	13	84	11	10.23133	35.8345933	3.502437448
Os06g0299300|AK060	Short-chain dehydrogenase/reductase SDR family protein.	41	20	12	30	89	136	24.3569133	85.23654	3.499480367
Os03g0702500|AK064	UDP-glucuronosyl/UDP-glucosyltransferase family protein.	309	261	297	1030	1004	997	288.845933	1010.26417	3.497588334
Os01g0674400|CI4391	(No Hit)	17	24	30	89	65	91	23.5104333	81.7480733	3.477097686
Os04g0471700|AY341	WRKY10 (WRKY transcription factor 35).	14	9	19	77	14	56	14.148049	49.0264333	3.465243394
Os07g0129200|AF251	PR1a protein.	1324	2406	1548	6168	5487	6628	1759.156	6094.40533	3.464391636
Os02g0483500|AK103	Transferase family protein.	54	31	31	135	175	92	38.7664267	134.086853	3.458839642
osa-miR395p|Os04|NA	miRNA	7	11	29	6	149	9	15.8139113	54.6373953	3.455020974
Os08g0412800|AK108	Protein of unknown function DUF1262 family protein.	9	7	6	30	26	20	7.34902967	25.3866133	3.454417043
Os07g0130800|AK067	Lectin-like receptor kinase 7;2.	26	28	32	98	116	82	28.6305733	98.82962	3.451891055
Os01g0138300|AK070	Protein kinase domain containing protein.	46	72	76	262	165	241	64.6370933	222.4863	3.442083926
Os01g0859200|AK099	Esterase/lipase/thioesterase domain containing protein.	15	11	11	49	40	40	12.4101067	42.7085	3.441428921
Os01g0319000|AK067	Pectinacetylesterase family protein.	97	125	125	412	455	325	115.850157	397.167567	3.428286833
Os01g0349600|AK066	Conserved hypothetical protein.	13	21	15	30	126	10	16.2417833	55.6523567	3.426492986
Os07g0677200|AK104	Peroxidase.	3328	3841	3615	14753	9703	12492	3594.498	12315.9687	3.426338996
Os07g0677200|X66125	Peroxidase.	2289	2490	2349	9575	6710	8104	2375.829	8129.65767	3.421819359
Os09g0454600|CI4441	Phosphate carrier protein, mitochondrial precursor (PTP). Splice isofor	32	27	37	108	126	96	32.1237033	109.87046	3.420230191
Os04g0476600|AK105	C2 domain containing protein.	83	54	40	51	526	30	59.1867133	202.28981	3.417824687
Os07g0287100|AK066	TPR-like domain containing protein.	49	45	45	75	353	47	46.26145	158.073937	3.416968916
Os08g0404500|AK103	C1-like domain containing protein.	64	86	75	287	240	239	74.7382467	254.935833	3.411049158
Os06g0107100|AK098	Protein of unknown function DUF819 family protein.	77	73	69	292	244	211	73.0897233	249.188467	3.409350252
Os04g0656500|AK068	LIGULELESS1 protein.	7	8	8	6	62	10	7.58563167	25.8558027	3.408523351
POsControl0007|genon	NONE	10	12	22	113	26	10	14.525596	49.4866843	3.40686085
POsControl0040|art|NA	NONE	9	8	6	7	25	45	7.49445267	25.5137837	3.404355835
Os08g0152400|CI1034	Cytochrome P450 family protein.	86	72	72	242	271	272	76.8918467	261.6582	3.40293817
Os02g0719600|AK070	SAM dependent carboxyl methyltransferase family protein.	8	10	7	29	32	20	7.916198	26.9301367	3.401902866
Os03g0714100|CI4435	(No Hit)	35	26	17	85	116	64	26.0460833	88.56246	3.400221786
Os03g0711100|AK120	CONSTANS-like protein.	9	7	6	18	45	12	7.34699667	24.9747567	3.399315094
Os07g0677200|AK099	Peroxidase.	3177	3671	3507	14146	9122	11889	3451.35467	11719.2527	3.395551544
Os02g0205500|AK067	Naringenin-chalcone synthase family protein.	707	747	653	2329	1839	2984	702.468667	2384.281	3.394145694
Os06g0107100|AK066	Protein of unknown function DUF819 family protein.	90	81	62	290	270	230	77.5569733	263.148133	3.392965481
Os04g0497000|AK069	(+)-pulegone reductase.	240	289	309	942	845	1055	279.403533	947.417333	3.390856665
Os11g0204800|AK100	Hypothetical protein.	15	26	18	81	60	61	19.8590033	67.3210267	3.389949915
Os02g0255900|Os02g	Conserved hypothetical protein.	46	69	41	54	395	78	51.8845167	175.761883	3.387559423
Os07g0190000|AK100	1-deoxy-D-xylulose 5-phosphate synthase 2 precursor.	1522	1460	1895	5717	4608	6167	1625.43067	5497.44767	3.382148362
Os10g0150800|AK061	Protein of unknown function DUF1210 family protein.	8	7	5	8	12	46	6.48474033	21.9320053	3.382094611
Os10g0575600|AK071	Homeobox domain containing protein.	9	24	6	7	14	111	12.9744863	43.742854	3.3714517
Os01g0625300|AK069	Heat shock transcription factor 31 (Fragment).	9	14	11	43	38	34	11.3071103	38.1023167	3.369766063
Os07g0461500|CI2581	(No Hit)	15	7	16	14	106	11	12.9375647	43.59113	3.369345864
Os03g0158600|CI2430	Conserved hypothetical protein.	14	11	8	13	21	77	10.991238	37.02617	3.368698776
Os07g0677200|AK073	Peroxidase.	1171	1325	1200	5065	3062	4311	1231.79	4145.98133	3.365818308

Os01g0287600|AK063(	Chitinase (Class II) (EC 3.2.1.14).	2751	2630	2774	9693	8342	9406	2718.69733	9147.03033	3.364490126
Os03g0784000|AK067:	Flavin-containing amine oxidase family protein.	8	23	5	99	12	10	12.057191	40.5613187	3.364076978
Os05g0217800|AK104!	BURP domain containing protein.	8	6	6	17	20	31	6.76631533	22.7573833	3.363334727
Os12g0501700|AY250(	Transcription factor, MADS-box domain containing protein.	7	7	5	42	14	10	6.50204333	21.8660983	3.362957952
Os04g0348800|Os04g(	Response regulator receiver domain containing protein.	11	8	8	43	37	10	8.89094233	29.8929067	3.362175295
Os07g0129200|AJ2784	PR1a protein.	1448	2554	1616	6373	5727	6748	1872.93567	6282.52867	3.354375048
Os01g0875800|AK105!	Arabidopsis protein of unknown function DUF266 family protein.	134	104	72	283	491	263	103.06671	345.6421	3.353576533
Os01g0337600|AK119.	Protein of unknown function DUF625 domain containing protein.	8	7	6	9	50	11	6.975687	23.370402	3.350265286
Os12g0628600|X68197	Thaumatin-like protein precursor.	69	49	34	102	259	147	50.5448433	169.249367	3.348499184
Os01g0186600|AK108	Conserved hypothetical protein.	20	18	7	32	73	45	14.8957047	49.84151	3.346032371
Os09g0551000|CI4088	Receptor-like protein kinase.	18	12	8	42	53	32	12.659098	42.33883	3.34453766
osa-miR167c|Os03|NA	miRNA	10	8	7	7	32	44	8.32173733	27.8252993	3.34368873
Os10g0349900|AK062	Plant lipid transfer/seed storage/trypsin-alpha amylase inhibitor domair	9	7	6	13	47	11	7.062041	23.57791	3.338682118
Os07g0119100|AU172.	Conserved hypothetical protein.	8	6	5	6	12	45	6.28786767	20.9868833	3.337678915
Os10g0130700|AK072!	Zn-finger, CCHC type domain containing protein.	23	15	18	6	172	10	18.7704767	62.636281	3.336957399
Os08g0374600|AY224.	Receptor kinase-like protein.	66	68	69	172	160	349	67.92072	226.595267	3.336172918
osa-miR395n|Os04|NA	miRNA	9	7	6	7	45	23	7.483775	24.958601	3.335028244
Os10g0514600|AK070:	T-complex protein 1 delta subunit.	8	10	6	8	62	10	8.077107	26.914439	3.332187998
Os11g0707000|AK119!	Ribulose-bisphosphate carboxylase activase (EC 6.3.4.-) (Fragments).	9	7	6	7	25	42	7.40631267	24.660489	3.329658105
Os07g0417600|CI5335	(No Hit)	104	33	23	213	153	167	53.37935	177.6701	3.328442553
Os04g0444800|AB126(	Cytochrome b-245, heavy chain family protein.	38	27	17	22	240	12	27.35544	91.0335633	3.327804756
Os08g0384500|AK100	PDR-like ABC transporter (PDR3 ABC transporter).	12	26	22	61	54	86	20.1062233	66.89618	3.327138015
Os03g0776400|AK106:	Conserved hypothetical protein.	146	156	168	549	492	519	156.492067	520.255933	3.324487589
osa-miR395v|Os09|NA	miRNA	15	6	6	11	44	33	8.83735133	29.3194267	3.317671275
POsControl0041|art|NA	NONE	8	6	5	6	46	10	6.20735633	20.5581833	3.311906427
Os03g0611100|AK102!	Hypothetical protein.	18	12	7	12	98	12	12.307373	40.72936	3.309346357
Os02g0529500|AK069!	SNAP25-like protein C (Fragment).	10	11	11	34	61	10	10.5941317	35.0324337	3.306777258
Os06g0694500|AK103!	Nitrogen fixation like protein.	59	63	46	218	151	183	55.65854	183.9224	3.304477624
Os05g0399800|CI4668	(No Hit)	28	12	11	36	84	50	17.1834533	56.7683133	3.303661507
Os03g0714100|CI4435	(No Hit)	22	29	17	63	103	57	22.5097533	74.3228633	3.301807098
Os02g0693700|AK103	MDR-like p-glycoprotein.	43	25	23	30	106	163	30.18385	99.5735267	3.298900792
Os04g0522500|AK107	Isopenicillin N synthase family protein.	16	20	6	31	45	62	13.9240193	45.9255367	3.298295957
Os11g0618200|CI4331	(No Hit)	52	45	30	144	182	96	42.5895633	140.408743	3.296787578
Os05g0318600|AK099	Protein kinase domain containing protein.	32	45	44	132	147	118	40.2182567	132.5185	3.294983696
Os05g0205000|AK062:	Multifunctional transport intrinsic membrane protein 2.	490	576	613	1963	1517	2052	559.892367	1844.15033	3.293758663
Os03g0132900|AK099:	Chitinase (EC 3.2.1.14) (Fragment).	117	108	103	290	364	424	109.247933	359.5056	3.290731358
Os03g0800000|AK064	Conserved hypothetical protein.	140	102	112	374	385	402	117.7326	387.011167	3.28720479
Os11g0672200|AK107	Protein kinase-like domain containing protein.	162	239	265	844	539	806	221.918133	729.412433	3.286853681
POsControl0034|rando	NONE	7	6	5	6	44	10	6.04059933	19.8209447	3.281287762
Os04g0178300|AK100(	Copalyl diphosphate synthetase (Fragment).	195	227	218	721	654	724	213.315567	699.779633	3.280490235
Os07g0129800|Os07g(	Protein kinase family protein.	46	42	51	162	150	144	46.4277367	152.264033	3.279591991

Os06g0216000|Os06g(	Oxo-phytodienoic acid reductase.	466	447	540	1409	1739	1615	484.1754	1587.75133	3.279289558
Os01g0810800|AK063	Receptor protein kinase-like protein.	46	34	19	24	282	18	32.9549867	107.726273	3.268891425
Os07g0620800|AK063(	Cyclin, N-terminal domain containing protein.	348	360	428	1465	883	1363	378.525633	1237.0644	3.268112622
Os12g0457500|AK073	Hypothetical protein.	31	27	28	22	37	222	28.6567033	93.57523	3.265387121
Os12g0555200|AK099	Probenazole-inducible protein PBZ1.	9	7	6	16	37	16	7.16354233	23.3855	3.2645162
Os06g0521500|AK072	Haem peroxidase, plant/fungal/bacterial family protein.	49	53	40	149	147	170	47.7172067	155.6677	3.262296997
Os10g0329700|CI2232	Serine/threonine protein kinase family protein.	10	8	7	23	16	45	8.55049633	27.8869533	3.26144264
Os04g0570500|AK103	Cytochrome P450 family protein.	9	7	6	6	51	11	7.00062833	22.7026077	3.242938574
Os04g0107700|AK110	Peptidase C1A, papain family protein.	9	7	6	6	21	42	7.111662	23.055705	3.241957365
Os01g0945100|CI3902	Amino acid/polyamine transporter I family protein.	92	88	122	302	335	342	100.640223	326.267167	3.241916163
Os04g0578600|AB126	Ferric reductase-like transmembrane component family protein.	10	7	6	6	55	11	7.44475633	24.1208147	3.239973692
Os10g0542900|AB016	Chitinase (EC 3.2.1.14) (Fragment).	1154	1276	1522	3863	3908	5031	1317.22167	4267.19133	3.239539283
Os07g0629000|AY323	P-type R2R3 Myb protein (Fragment).	91	77	70	218	272	278	79.1903567	256.113833	3.23415431
Os05g0267000|Os05g(	Galactose oxidase, central domain containing protein.	8	7	6	7	50	10	6.95313867	22.4683647	3.231398904
Os12g0609100|AK107	Hypothetical protein.	11	6	6	12	23	38	7.502532	24.2354333	3.230300562
Os01g0319200|Os01g(	Plant protein of unknown function family protein.	136	112	114	398	330	443	120.850833	390.2844	3.229472145
Os04g0243500|Os04g(	Retrotransposon gag protein family protein.	8	8	17	15	31	61	11.0147923	35.5608833	3.228466072
Os03g0180900|AK073	ZIM domain containing protein.	1296	1377	1720	4537	3731	5911	1464.30867	4726.25167	3.227633473
Os12g0172400|Os12g(	Hypothetical protein.	8	22	19	71	48	39	16.3203297	52.6173167	3.22403516
Os02g0103800|AK065	Ferredoxin--NADP reductase, leaf isozyme, chloroplast precursor (EC	9	7	6	28	20	21	7.181587	23.1522467	3.223834323
Os06g0184900|AK109	Transferase family protein.	10	8	7	7	57	13	7.95037967	25.6010297	3.220101522
Os02g0738200|CI5481	U box domain containing protein.	144	125	122	343	484	434	130.636867	420.6078	3.219671527
Os07g0264000|AK072(	Hypothetical protein.	8	6	7	17	22	31	7.198337	23.17127	3.218975438
Os11g0687200|AK070	Hypothetical protein.	113	120	121	198	363	580	118.125167	380.1706	3.218370909
Os11g0707000|AK060	Ribulose-bisphosphate carboxylase activase (EC 6.3.4.-) (Fragments).	11	6	6	6	57	11	7.67510633	24.6549263	3.212323747
Os11g0242200|CI2458	(No Hit)	10	7	7	31	22	23	7.82952767	25.1509067	3.212314681
POsControl0048|art|NA	NONE	7	6	5	6	43	10	6.01486167	19.3188953	3.211860289
Os10g0485600|Os10g(	Type III restriction enzyme, res subunit family protein.	86	85	75	74	180	536	82.0692167	263.455397	3.210160976
Os02g0198700|AK120	Subtilisin-like protease.	285	208	275	805	817	842	255.944333	821.4638	3.209540877
Os04g0665600|AK101	Phosphate starvation response regulator-like protein.	123	134	131	449	368	430	129.498333	415.4752	3.208343994
Os01g0701400|Os01g(	Cytochrome P450 family protein.	452	553	748	2092	1370	2161	584.196333	1874.27133	3.208290135
Os06g0549900|AK109(	FAD linked oxidase, N-terminal domain containing protein.	9	7	6	17	23	27	7.02237733	22.5240267	3.207464595
Os01g0839400|Os01g(	TPR-like domain containing protein.	17	20	14	16	38	110	17.07011	54.60161	3.19866773
POsControl0034|rando	NONE	9	7	6	7	55	12	7.61197533	24.320635	3.195049108
Os01g0691200|AK111	Ankyrin repeat containing protein.	17	14	6	8	100	10	12.2768797	39.200429	3.19302869
Os08g0203400|AK068	Protein kinase domain containing protein.	13	8	9	45	28	23	9.92665267	31.6830567	3.191716053
Os05g0217800|AK104	BURP domain containing protein.	80	67	55	217	226	201	67.21877	214.533167	3.191566383
Os01g0957800|CI3979	Cytochrome P450 family protein.	36	65	47	209	127	137	49.4316767	157.6898	3.190055661
POsControl0039|art|NA	NONE	8	6	6	12	43	10	6.86348733	21.8831567	3.18834371
Os07g0677200|AF014	Peroxidase.	1205	1287	1242	3808	3570	4520	1244.68833	3966.19367	3.186495415
Os06g0593200|AK060	UDP-glucuronosyl/UDP-glucosyltransferase family protein.	202	188	171	604	577	605	186.8361	595.226933	3.18582401

Os03g0575200|AJ4279	K+ potassium transporter family protein.	66	64	41	116	248	180	56.8955367	181.234767	3.185395152
Os02g0161300|AK0709	Hypothetical protein.	22	18	27	31	28	154	22.3266367	71.0988067	3.184483526
Os07g0188700|AK1076	Exostosin-like family protein.	13	15	5	15	80	10	10.9479167	34.8634117	3.184479087
Os07g0141100|AK1063	Protein kinase domain containing protein.	235	373	360	1162	899	1021	322.649133	1027.28607	3.18391082
Os05g0507600|Os05g(	Hypothetical protein.	9	7	6	11	49	11	7.46757133	23.77282	3.183474109
Os02g0768000|AK1066	Conserved hypothetical protein.	115	380	151	729	568	758	215.257833	685.169033	3.183015562
Os10g0149800|CI0974	Protein of unknown function DUF1210 family protein.	35	33	9	44	185	14	25.55914	81.2579933	3.179214689
Os06g0248300|AK0701	Conserved hypothetical protein.	19	10	5	17	81	10	11.2991903	35.896033	3.176867717
Os10g0391400|AK1078	ZIM domain containing protein.	572	558	599	1859	1497	2132	576.200133	1829.24367	3.174667205
Os03g0853200|AK1091	CD9/CD37/CD63 antigen family protein.	20	70	68	160	139	202	52.6921133	167.110867	3.171458803
Os04g0178300|AY5301	Copalyl diphosphate synthetase (Fragment).	371	430	452	1303	1287	1382	418.0026	1323.949	3.167322404
Os01g0209800|AK0615	Cationic amino acid transporter (Fragment).	424	454	461	942	1516	1780	446.130667	1412.46317	3.166030206
Os02g0198700|AK1063	Subtilisin-like protease.	249	192	230	735	630	761	223.890467	708.608867	3.164980078
osa-miR395h|Os08|NA	miRNA	8	6	5	6	44	10	6.36990533	20.151542	3.163554393
Os10g0130200|Os10g(	Cyclin-like F-box domain containing protein.	104	105	102	330	324	327	103.53	327.122067	3.159683828
Os04g0565400|CI4834	Cis-zeatin O-glucosyltransferase.	9	10	10	36	31	24	9.59274267	30.2775433	3.156296837
Os08g0224300|Os08g(	CemA family protein.	20	22	10	27	32	103	17.0587783	53.8142467	3.154636611
Os01g0816600|CI5557	Protein kinase domain containing protein.	132	124	116	445	320	409	123.983667	391.025333	3.153845533
Os02g0462800|AK1105	WRKY transcription factor 42 (Transcription factor WRKY02).	68	51	39	181	131	184	52.3994233	165.217533	3.153041061
Os08g0404500|AK1089	C1-like domain containing protein.	65	91	68	275	192	238	74.5767233	235.137967	3.152967255
Os02g0646200|AK0715	Zn-finger, B-box domain containing protein.	647	564	552	1651	1571	2330	587.701567	1850.73367	3.149104531
Os08g0499700|AK1097	Conserved hypothetical protein.	114	80	52	75	651	52	82.3430167	259.248093	3.148391981
Os03g0412400|AK1105	Conserved hypothetical protein.	162	185	162	506	527	568	169.500767	533.6206	3.14818989
Os02g0185500|CI2281	Cytochrome P450 family protein.	8	6	5	7	12	42	6.41902767	20.1955327	3.146198103
Os02g0179200|AK1113	Glutamine amidotransferase class-I domain containing protein.	88	112	120	363	303	339	106.572273	334.966233	3.143089876
Os08g0202300|AK0672	Leucine-rich repeat, plant specific containing protein.	7	6	5	36	12	10	6.12335733	19.246023	3.143050773
Os01g0347300|CI5505	(No Hit)	98	148	123	431	330	396	122.9018	385.541267	3.136986331
Os10g0167200|Os10g(	Geraniol 10-hydroxylase (EC 1.14.14.1).	42	22	18	25	218	11	27.0747567	84.9179833	3.136426465
Os01g0876200|AK1194	Hypothetical protein.	10	16	10	31	52	31	12.1134867	37.97731	3.135126248
Os10g0150400|AK0615	Protein of unknown function DUF1210 family protein.	10	8	7	31	32	13	8.143763	25.44523	3.12450522
Os12g0495700|CI1422	(No Hit)	35	25	18	22	83	137	25.84046	80.64942	3.121052025
Os08g0113900|AK0623	Conserved hypothetical protein.	24	37	14	57	104	74	25.0349033	78.09402	3.119405694
Os09g0268500|AK0997	Hypothetical protein.	9	7	6	7	51	11	7.29828933	22.7649457	3.119216658
Os05g0125400|CB6609	Receptor protein kinase-like protein.	10	7	5	12	46	10	7.220096	22.5057817	3.117102829
Os02g0535000|AK0739	Conserved hypothetical protein.	288	340	352	1190	724	1132	326.531333	1015.5457	3.110101838
Os05g0469900|AK1097	Conserved hypothetical protein.	16	14	11	17	101	11	13.76885	42.8140367	3.1094853
Os03g0731800|AK1055	Hypothetical protein.	32	34	19	34	77	153	28.2528167	87.8365067	3.108946896
POsControl0011|genon	NONE	34	26	24	74	175	10	27.77519	86.3061427	3.107310613
Os12g0165900|Os12g(	Peptidase S59, nucleoporin family protein.	13	7	20	15	99	11	13.40227	41.6292467	3.106134011
Os11g0213600|AK1034	Peptidase S10, serine carboxypeptidase family protein.	236	247	266	873	606	850	250.032767	776.522033	3.105681082
Os01g0777000|Os01g(	Conserved hypothetical protein.	321	350	373	1249	739	1253	347.922267	1080.47203	3.105498374

Os01g0883800|AY114:	Gibberellin 20-oxidase.	79	77	194	411	330	346	116.803663	362.410367	3.102731167
Os01g0829900|AK070(	Oligopeptide transporter OPT superfamily protein.	7	6	5	6	41	10	6.056032	18.790115	3.102710653
Os01g0860500|AK103!	Chitinase (EC 3.2.1.14).	42	46	28	117	129	115	38.77439	120.155733	3.098842647
Os05g0537100|CI4308	WRKY transcription factor 10.	732	703	785	2172	2157	2547	739.742333	2291.96933	3.09833469
Os05g0217800|AK105!	BURP domain containing protein.	75	68	54	222	184	200	65.3138033	202.173567	3.095418676
Os05g0199500|AK063:	Glycosyl transferase, family 31 protein.	17	6	21	23	86	27	14.5895107	45.1029533	3.091464434
Os07g0482900|Os07g(	Cytochrome P450 family protein.	71	98	111	276	246	345	93.48583	288.986133	3.091229263
Os03g0678100|Os03g(	Protein kinase domain containing protein.	49	47	29	36	312	38	41.7733533	129.078903	3.089981843
Os12g0576200|CI4940	(No Hit)	9	7	6	7	52	11	7.503099	23.1844057	3.08997731
Os04g0301500|AB040	Basic helix-loop-helix dimerisation region bHLH domain containing prot	303	210	226	711	691	875	246.067733	759.174267	3.085224773
Os09g0510500|AK066	Basic helix-loop-helix dimerisation region bHLH domain containing prot	55	49	50	150	178	148	51.39314	158.554667	3.085132893
Os02g0202400|AK107:	Brittle-1 protein, chloroplast precursor.	8	6	5	6	13	42	6.59083367	20.293011	3.078974835
Os09g0116900|AK071!	Hypothetical protein.	39	30	25	31	88	172	31.5577633	96.9186733	3.071151536
Os01g0157600|AK106	Ankyrin repeat containing protein.	8	6	5	7	43	10	6.499463	19.93217	3.066741052
osa-miR419|Os12|NA|c	miRNA	10	7	5	34	25	10	7.38536367	22.6334247	3.064632385
Os04g0528200|AK064(	Conserved hypothetical protein.	10	8	7	8	56	12	8.28205333	25.3458563	3.060334836
Os03g0345300|AK109:	Conserved hypothetical protein.	8	7	6	7	46	11	6.90784367	21.130363	3.058894211
Os08g0539700|AK099!	PibH8 protein.	45	46	43	31	87	290	44.4344667	135.799583	3.056176737
Os07g0108500|AK071(	C2 domain containing protein.	8	6	5	36	12	10	6.40445367	19.5597133	3.054079919
osa-miR442|Os04|NA|c	miRNA	30	24	20	44	166	12	24.34668	74.25877	3.050057338
Os11g0556100|AK111:	Hypothetical protein.	10	23	12	113	12	10	14.8488133	45.2595733	3.048026284
Os08g0222800|CI1415	(No Hit)	137	105	67	88	781	72	102.926683	313.68935	3.047697058
Os05g0530400|AB050(	Heat shock factor protein 1 (HSF 1) (Heat shock transcription factor 1)	50	56	59	204	139	159	55.03971	167.556867	3.044290507
osa-miR319b|Os01|NA	miRNA	15	14	7	15	39	54	11.8581367	36.0872633	3.043249066
Os03g0332500|C9758!	60S ribosomal protein L10 (QM protein homolog).	32	7	11	10	132	12	16.8313823	51.2095003	3.042501164
Os01g0944500|Os01g(	Glycoside hydrolase, family 17 protein.	468	613	553	1676	1789	1501	544.3297	1655.40833	3.041186864
Os10g0464000|AK103	Hypersensitive-induced response protein.	1138	1580	1614	4965	4082	4119	1443.99633	4388.709	3.039279878
Os03g0318400|AK106	Peptidase A1, pepsin family protein.	451	444	417	1138	1138	1711	437.253467	1328.70867	3.038760737
Os01g0826400|AK107	WRKY transcription factor 24.	362	347	413	1135	964	1306	373.858767	1134.88873	3.035608188
Os10g0425900|AK107:	BTB/POZ domain containing protein.	8	6	6	6	12	42	6.68496533	20.2924927	3.035541944
osa-miR169j|Os09|NA|(	miRNA	9	7	6	7	14	48	7.61844367	23.1234103	3.035188202
Os01g0136300|AK062	Conserved hypothetical protein.	633	788	811	2692	1666	2415	743.7901	2257.50733	3.035140335
Os10g0390900|Os10g(	Synaptojanin, N-terminal domain containing protein.	42	41	36	31	185	146	39.8323833	120.871997	3.034515802
Os03g0216000|AK109!	Zn-finger, C2H2 type domain containing protein.	24	35	15	19	43	162	24.6789667	74.87465	3.033945911
Os02g0215700|AK099	Protein kinase domain containing protein.	19	23	12	53	66	43	17.8470267	54.0751533	3.029925059
Os01g0214500|AK062	Conserved hypothetical protein.	220	331	329	1057	615	991	293.2326	887.4361	3.026389631
Os02g0807900|AK119:	Serine threonine kinase.	9	15	6	23	13	56	10.2188107	30.8946267	3.023309431
Os05g0217800|AK104(	BURP domain containing protein.	77	66	48	202	186	192	63.9204867	193.1592	3.021866855
Os01g0934600|CB656	Esterase/lipase/thioesterase domain containing protein.	8	6	5	9	39	10	6.33981167	19.1564247	3.021607845
Os12g0283300|CI4069	(No Hit)	7	6	8	41	14	10	7.17217067	21.620373	3.014481111
Os01g0113900|AK068	Conserved hypothetical protein.	10	8	7	7	55	12	8.268202	24.923023	3.014321977

Os08g0472800|AK120	E-class P450, group I family protein.	198	256	250	810	596	710	234.739933	705.604967	3.005900857
Os03g0428200|AK103	Terpene synthase, metal-binding domain containing protein.	10	6	5	29	24	10	6.99294933	21.0020947	3.003324301
Os11g0640400|AK101	Hypothetical protein.	91	67	52	62	208	358	69.80518	209.4348	3.000275911
Os01g0138300|AK099	Protein kinase domain containing protein.	46	75	91	246	165	227	70.8423367	212.526167	2.999988096
Os02g0103800|AK061	Ferredoxin--NADP reductase, leaf isozyme, chloroplast precursor (EC	10	10	8	33	40	13	9.50067033	28.4963067	2.999399586
Os09g0518600|Os09g	Conserved hypothetical protein.	45	43	37	121	157	98	41.71349	125.05183	2.997875028
Os07g0215500|AF042	Allergenic protein.	8	6	6	6	45	11	6.82504233	20.4480587	2.996033968
Os07g0440700|AK108	Non-protein coding transcript, uncharacterized transcript.	14	14	17	12	14	111	15.2116467	45.5519633	2.994545188
Os06g0353700|CI4392	(No Hit)	92	77	70	279	229	210	79.8402133	239.068767	2.994340279
Os10g0469700|AK066	Leucine-rich repeat, cysteine-containing type containing protein.	125	142	135	386	371	443	133.785967	400.192333	2.991287826
Os01g0860500|AK058	Chitinase (EC 3.2.1.14).	52	67	60	177	115	246	59.95034	179.3246	2.991219066
Os01g0597600|AU056	Amino acid/polyamine transporter II family protein.	269	276	242	838	730	785	262.391533	784.546867	2.989985449
Os11g0572700|CI0479	FAD-dependent pyridine nucleotide-disulphide oxidoreductase family p	278	308	346	1019	749	1019	310.7036	928.839333	2.98947078
Os04g0121800|AK121	Non-protein coding transcript, uncharacterized transcript.	37	42	62	155	103	160	46.6647867	139.392933	2.987111767
Os01g0117300|Os01g	Protein kinase domain containing protein.	23	14	6	15	57	55	14.1616447	42.2765567	2.985285796
Os03g0289800|AK110	Isopenicillin N synthase family protein.	90	98	73	250	262	267	86.98171	259.644767	2.985050152
Os01g0135600|CI3238	(No Hit)	37	46	48	129	110	152	43.7152567	130.4828	2.984834356
Os01g0705700|AK064	Transcription factor ICE1 (Inducer of CBF expression 1) (Basic helix- lo	347	414	392	1303	964	1176	384.480367	1147.54487	2.98466441
Os01g0826400|AY341	WRKY transcription factor 24.	368	334	380	1045	960	1222	360.827	1075.49117	2.980628297
Os06g0694500|AK067	Nitrogen fixation like protein.	104	127	109	406	250	356	113.2073	337.386733	2.980255985
Os01g0685400|CI5441	Blind.	393	427	422	1236	1208	1261	414.4265	1235.04433	2.980128764
Os04g0494100|AK104	Endochitinase A precursor (EC 3.2.1.14) (Seed chitinase A).	8	6	8	6	27	30	7.10003133	21.1329097	2.976453015
Os11g0630300|AK066	Hypothetical protein.	11	7	6	6	52	11	7.74512667	23.0353777	2.974177009
Os07g0488200|CI1394	(No Hit)	628	653	655	2019	1911	1825	645.531267	1918.13133	2.971399578
Os08g0287200|CI4709	Conserved hypothetical protein.	9	7	6	7	30	27	7.22208567	21.4441953	2.969252419
Os10g0148700|AK071	Protein of unknown function DUF1210 family protein.	8	6	5	6	41	10	6.50372967	19.2993657	2.967430483
Os02g0684200|Os02g	Hypothetical protein.	103	68	56	74	269	328	75.4568	223.73078	2.96501813
Os06g0316000|AK058	Hypothetical protein.	8	6	7	12	32	16	6.77013667	20.0723167	2.964831828
Os01g0109500|AK058	Conserved hypothetical protein.	121	107	78	88	740	79	102.221597	302.49022	2.959161565
Os12g0285100|AK100	ARM repeat fold domain containing protein.	34	33	23	40	72	155	29.9651567	88.64905	2.958404356
POsControl0039|art|NA	NONE	9	7	18	7	82	11	11.232937	33.2159457	2.957013439
Os09g0267400|AK070	Harpin-induced 1 domain containing protein.	19	13	5	18	81	10	12.3253663	36.4341393	2.95602892
POsControl0023|genon	NONE	9	7	6	6	46	11	7.14589767	21.1208133	2.955655723
Os01g0859200|AK072	Esterase/lipase/thioesterase domain containing protein.	160	180	217	646	447	552	185.612833	548.5955	2.955590355
Os01g0613800|AK110	Peptidase C1A, papain family protein.	149	115	99	310	357	405	120.923433	357.2727	2.954536521
Os06g0726100|Z29962	Endochitinase precursor (EC 3.2.1.14).	131	181	219	374	567	628	176.9352	522.720367	2.954303986
Os03g0661600|AK058	Alpha-amylase/trypsin inhibitor (Antifungal protein).	49	71	52	118	207	182	57.3068267	169.287233	2.954050035
Os03g0623100|AK063	Conserved hypothetical protein.	9	7	6	7	48	11	7.501417	22.1304147	2.950164571
Os03g0600600|Os03g	Beta-1,3-glucanase precursor.	33	43	35	61	151	117	37.17462	109.65773	2.949800966
Os02g0761700|AK068	Methionine aminopeptidase-like protein.	20	13	5	14	89	10	12.7455967	37.5513457	2.946221087
Os11g0695000|CI5097	(No Hit)	18	17	10	31	61	41	15.1014167	44.4830867	2.945623424

Os10g0470700|AK101(	Peptide transporter.	468	454	493	1355	1395	1419	471.929067	1389.84033	2.945019562
POsControl0040|art|NA	NONE	7	8	5	34	17	9	6.739299	19.845695	2.944771407
Os04g0650700|AK061!	L-asparaginase (L-asparagine amidohydrolase).	12	18	10	51	33	34	13.3643047	39.35349	2.944671719
Os05g0324700|AK063!	Hypothetical protein.	266	313	299	952	690	943	292.6783	861.676167	2.944106778
Os04g0508000|AK071	Protein of unknown function DUF231 domain containing protein.	9	19	9	18	57	34	12.314378	36.25273	2.94393513
Os09g0446100|Os09g(	Hypothetical protein.	8	6	6	6	42	10	6.594334	19.3868037	2.93991837
Os09g0257800|CI5335	(No Hit)	19	8	10	16	78	16	12.5041363	36.7543367	2.939374275
Os12g0154700|AK063(	Germin family protein.	9	7	6	29	14	22	7.33023267	21.5449967	2.939196837
Os05g0583000|AK109!	WRKY transcription factor 34.	19	6	12	36	48	28	12.6035793	37.0399267	2.938841871
Os07g0218200|CI1632	(No Hit)	44	16	14	17	159	43	24.82157	72.92759	2.938073216
Os06g0147000|AK120!	Conserved hypothetical protein.	840	958	1138	3391	2202	3029	978.8287	2873.90133	2.936061574
POsControl0046|art|NA	NONE	13	6	6	16	44	12	8.13308067	23.8363567	2.930790637
Os09g0555100|CI4395	Sulfotransferase family protein.	57	97	84	238	214	244	79.22023	232.0071	2.928634517
Os01g0113500|AU166:	Protein kinase domain containing protein.	27	26	32	80	88	81	28.4094233	83.10666	2.925320202
osa-miR439d|Os03|NA	miRNA	11	9	5	10	56	10	8.632235	25.2503047	2.925117848
Os05g0343200|CI0409	(No Hit)	14	20	16	12	15	117	16.4151533	47.99245	2.923667481
Os08g0135800|AK063(	Zn-finger, C-x8-C-x5-C-x3-H type domain containing protein.	37	26	23	17	74	157	28.4373733	82.9486333	2.916888011
Os06g0340200|AK121:	Conserved hypothetical protein.	14	18	23	26	125	11	18.4038	53.66488	2.915967355
Os06g0137600|AK099	Ribosome-binding factor A family protein.	7	11	5	40	18	9	7.780339	22.679666	2.914997149
Os03g0206800|AK111(	Hypothetical protein.	7	8	5	6	43	9	6.756995	19.6914153	2.914226714
Os11g0131200|Os11g(	Conserved hypothetical protein.	9	6	5	6	45	10	6.95926033	20.2764723	2.913595894
POsControl0031|rando	NONE	13	7	6	16	44	13	8.445925	24.5620367	2.908152354
Os10g0552400|AK121	U box domain containing protein.	345	463	483	1399	1037	1311	430.166367	1249.056	2.903657972
Os05g0526700|AK108	Harpin-induced 1 domain containing protein.	20	13	16	44	57	42	16.4386467	47.7304233	2.903549441
POsControl0012|genon	NONE	13	16	12	7	30	82	13.6098733	39.493295	2.901812091
Os02g0828100|AK070	Conserved hypothetical protein.	55	50	63	69	338	80	55.92622	162.22218	2.900646244
Os03g0130100|AK110	AMP-binding protein (Adenosine monophosphate binding protein 5 AM	96	102	88	224	381	223	95.23658	276.1682	2.899812236
Os08g0493100|AK121:	Cyclin-like F-box domain containing protein.	7	6	5	7	38	10	6.198743	17.9725323	2.899383364
Os05g0400100|AK107!	Dienelactone hydrolase domain containing protein.	11	7	6	7	53	12	8.22926467	23.8237553	2.895004147
Os02g0775300|AK111(	Conserved hypothetical protein.	192	229	283	773	591	675	234.821367	679.654833	2.894348342
Os03g0317100|CI4838	(No Hit)	58	60	39	40	150	264	52.3581833	151.53759	2.894248432
Os10g0524400|AK110:	Phospholipase D (EC 3.1.4.4).	8	8	12	13	49	20	9.49637833	27.47232	2.892926022
Os11g0642400|CI4345	(No Hit)	8	8	6	9	33	22	7.503563	21.6747533	2.888594836
Os01g0860500|AK099	Chitinase (EC 3.2.1.14).	50	44	36	114	136	127	43.5587	125.666867	2.885000394
Os04g0521600|AK102	Zinc-containing alcohol dehydrogenase family protein.	7	6	5	32	12	10	6.18939233	17.854743	2.884732788
Os03g0749800|AK111	Protein kinase.	12	17	13	14	98	10	13.99111	40.341325	2.883354144
Os09g0572700|AK063	Plastocyanin-like domain containing protein.	96	115	113	266	378	291	107.999687	311.378967	2.883146945
osa-miR435|Os03|NA|c	miRNA	19	8	7	18	65	12	10.9885367	31.6756467	2.88260827
Os03g0371400|AK107	Cytochrome P450 family protein.	10	8	7	27	25	16	7.996095	23.03818	2.881178875
Os06g0530400|AK102	No apical meristem (NAM) protein domain containing protein.	9	7	6	6	13	42	7.08430033	20.4092657	2.880914798
Os08g0137400|AK120!	Plastocyanin-like domain containing protein.	34	46	50	46	290	39	43.3378567	124.768413	2.878970557

Os06g0718400|CI5625	(No Hit)	18	16	13	17	91	29	15.8167067	45.53157	2.878701044
Os05g0202800|CI4464	Plant metallothionein, family 15 protein.	4641	4681	5033	17240	12451	11624	4784.983	13771.73	2.878114718
Os03g0657400|AU057	WRKY transcription factor 60.	8	8	6	8	46	10	7.375348	21.2264177	2.878022524
Os01g0859200|AK0618	Esterase/lipase/thioesterase domain containing protein.	245	281	271	824	677	793	265.8105	764.654467	2.876690224
Os01g0651300|AK111	GDSL-motif lipase/hydrolase-like protein.	8	6	5	6	37	12	6.39147767	18.378212	2.875424582
osa-miR397b|Os02|NA	miRNA	50	45	30	37	129	192	41.5642067	119.42666	2.873305413
Os12g0441600|Os12g	O-methyltransferase ZRP4 (EC 2.1.1.-) (OMT).	12	8	6	28	36	11	8.75722833	25.1394833	2.870712328
Os01g0110700|AK119	Phosphoenolpyruvate carboxylase (EC 4.1.1.31).	17	21	8	24	89	19	15.3597023	44.0529167	2.86808401
Os05g0181700|AU093	Hypothetical protein.	178	253	272	709	495	810	234.110767	671.263433	2.867289885
Os10g0483400|AK1208	Protein kinase domain containing protein.	45	59	43	127	142	152	48.8845233	140.1585	2.867134431
Os04g0128700|AK107	Conserved hypothetical protein.	356	359	293	868	887	1134	335.856233	962.905733	2.867017604
Os01g0532700|AK100	Hypothetical protein.	7	6	7	32	12	15	6.80272833	19.4991367	2.866370037
Os01g0111600|AK110	Phosphatidylethanolamine-binding protein family protein.	10	7	7	7	14	46	7.80730933	22.3742093	2.865802849
Os07g0147700|CI5152	ATPase, BadF/BadG/BcrA/BcrD type domain containing protein.	872	937	1076	3179	2117	2962	961.814467	2752.63467	2.861918553
POsControl0045|art|NA	NONE	13	11	9	33	50	12	11.0271697	31.5051333	2.857046213
Os04g0548500|AK063	Non-protein coding transcript, uncharacterized transcript.	298	272	208	704	884	634	259.400633	740.736233	2.85556833
Os02g0616300|Os02g	Stem cell self-renewal protein Piwi domain containing protein.	8	6	5	6	38	10	6.31824067	18.036026	2.854596232
Os08g0190100|AF1418	Oxalate oxidase-like protein or germin-like protein (Germin-like 8) (Ger	313	309	281	788	920	867	300.828367	858.485467	2.853738416
Os04g0645100|AK100	TPR-like domain containing protein.	530	827	839	2530	1439	2297	731.971367	2088.66733	2.853482292
Os02g0764500|AK061	Lhca5 protein.	38	34	19	24	218	20	30.53379	87.1172733	2.853143135
Os05g0217800|AK104	BURP domain containing protein.	8	6	5	16	29	10	6.53509067	18.63013	2.850783708
Os02g0123800|CI4243	(No Hit)	10	8	7	19	15	36	8.25374333	23.4754633	2.844220178
Os07g0561800|AK108	Hypothetical protein.	2775	2558	2836	7064	7964	8185	2722.99167	7737.66	2.841602527
Os05g0382000|AK121	Arf GTPase activating protein family protein.	11	6	6	9	46	10	7.74943933	22.013714	2.840684733
Os09g0255400|AK071	Indole-3-glycerol phosphate synthase, chloroplast precursor (EC 4.1.1.	60	49	72	146	182	185	60.3413467	171.049233	2.834693668
Os12g0419700|AK071	Non-protein coding transcript, uncharacterized transcript.	19	24	28	44	61	95	23.4391167	66.4326867	2.834265796
Os04g0321100|CI2509	UDP-glucuronosyl/UDP-glucosyltransferase family protein.	2373	3002	2824	7975	7337	7895	2733.12667	7735.72067	2.83035571
Os09g0494300|AK107	CW-type Zn-finger domain containing protein.	8	6	5	10	35	10	6.420167	18.1709733	2.830296055
Os08g0422000|AK067	Conserved hypothetical protein.	13	8	13	57	26	17	11.745268	33.2341633	2.82957897
POsControl0028|rando	NONE	24	11	6	6	99	10	13.6541533	38.629693	2.829153303
Os05g0442400|AK107	Transcription factor MYBS3.	58	67	45	153	121	206	56.6407467	160.2188	2.828684462
Os06g0147300|AK111	Conserved hypothetical protein.	60	107	115	306	210	281	93.9273033	265.671733	2.828482496
Os08g0404000|CI2509	UDP-glucuronosyl/UDP-glucosyltransferase family protein.	2221	2872	2681	7681	6782	7518	2591.268	7327.11533	2.827617727
Os08g0381000|AK107	Conserved hypothetical protein.	16	12	7	14	77	10	11.931601	33.7306867	2.827004244
Os08g0509100|AK066	Lipoxygenase, chloroplast precursor (EC 1.13.11.12).	8	6	6	6	41	10	6.664119	18.838412	2.826842078
Os07g0487100|CI1394	UDP-glucuronosyl/UDP-glucosyltransferase family protein.	659	674	688	1899	2056	1759	673.819367	1904.766	2.826819908
Os05g0215000|AK059	BURP domain containing protein.	14	7	6	19	47	12	9.18093633	25.90619	2.821737245
Os10g0482900|AK071	Glutaredoxin domain containing protein.	60	62	55	141	114	242	58.69159	165.603067	2.821580855
Os12g0516900|AK101	Cyclin-like F-box domain containing protein.	32	35	28	27	230	10	31.5676333	89.041618	2.820661817
Os12g0198200|CI2795	Protein phosphatase 2C family protein.	106	95	91	271	269	285	97.5615067	275.105933	2.819820467
Os01g0875500|AK101	Beta-galactosidase (EC 3.2.1.23).	84	71	66	245	185	194	73.6756067	207.644667	2.818363853

Os05g0120800|AK066	Conserved hypothetical protein.	37	26	38	36	192	59	33.95068	95.68535	2.818363285
Os11g0117900|CI4332	(No Hit)	153	243	239	636	415	737	211.5845	595.8423	2.81609617
Os06g0136600|AK069	Enolase 1 (EC 4.2.1.11) (2-phosphoglycerate dehydratase 1) (2-phosp	1078	1070	1059	3074	2843	3111	1068.91467	3009.33233	2.815315784
Os07g0564500|AK121	FAD-dependent pyridine nucleotide-disulphide oxidoreductase domain	8	6	5	6	12	35	6.25747567	17.5982403	2.812354577
Os01g0731100|AK109	Conserved hypothetical protein.	21	53	38	102	135	78	37.2892667	104.816823	2.810911361
Os05g0428300|CI2823	(No Hit)	16	10	5	13	66	10	10.4426323	29.3478093	2.81038424
Os08g0118100|Os08g	Amino acid-binding ACT domain containing protein.	1184	1195	1354	3993	2839	3659	1244.593	3496.941	2.809706466
Os09g0551100|AK107	Conserved hypothetical protein.	8	6	6	6	41	10	6.85991567	19.2624527	2.807972226
Os07g0470900|CI4328	Hypothetical protein.	21	10	6	44	25	32	12.09803	33.9548933	2.806646482
osa-miR396c|Os02|NA	miRNA	20	6	5	6	71	10	10.280937	28.8511963	2.806280822
Os05g0217800|AK106	BURP domain containing protein.	75	72	56	208	175	186	67.6653133	189.6532	2.802812707
Os11g0527100|AK101	Hypothetical protein.	9	10	12	28	48	11	10.3644283	29.0384033	2.801737095
Os08g0481200|AK072	Arm repeat containing protein.	10	8	7	7	49	12	8.13100767	22.7788667	2.801481391
Os04g0580700|AK070	MADS box transcription factor MADS17.	8	6	6	6	39	10	6.61945533	18.540318	2.800882711
Os05g0293500|AK072	Probable pectate lyase 18 precursor (EC 4.2.2.2) (Pectate lyase A10).	74	61	39	47	174	265	58.00095	162.33907	2.798903639
Os09g0460700|AK108	Esterase/lipase/thioesterase domain containing protein.	43	48	36	97	144	113	42.12916	117.898593	2.798503301
Os01g0557200|AU174	Alpha/beta hydrolase fold domain containing protein.	19	7	7	7	73	12	10.9928797	30.76103	2.7982686
Os01g0735900|AK107	UDP-glucuronosyl/UDP-glucosyltransferase family protein.	15	15	14	31	57	36	14.7458733	41.2595667	2.798041576
Os03g0396200|AK106	Protein prenyltransferase domain containing protein.	36	44	43	50	247	47	41.0206433	114.760773	2.797634654
Os10g0570200|AK063	RIR1b protein precursor.	57	74	81	175	219	198	70.6210433	197.4399	2.795765832
Os03g0251100|CB632	Hypothetical protein.	7	6	5	6	35	10	6.083263	16.9994773	2.794466939
Os03g0422600|Os03g	Conserved hypothetical protein.	22	13	10	19	47	59	14.95914	41.7761767	2.79268572
Os03g0140300|AK107	Hypothetical protein.	8	6	6	27	12	17	6.655159	18.5649033	2.789550683
osa-miR395d|Os04|NA	miRNA	25	23	10	21	127	14	19.4450833	54.2201233	2.788371868
Os04g0420600|Os04g	Serine/threonine protein kinase family protein.	38	32	38	112	96	93	36.0314767	100.257587	2.782500079
Os10g0530500|AF402	Glutathione S-transferase, C-terminal domain containing protein.	178	236	54	708	376	215	155.683557	433.028	2.781462662
Os10g0469900|AK105	TGF-beta receptor, type I/II extracellular region family protein.	8	13	5	15	48	10	8.71323433	24.2286333	2.780670461
Os04g0595700|CI2497	(No Hit)	65	48	45	136	199	104	52.7661733	146.464367	2.775724625
Os12g0106200|AK109	Protein of unknown function DUF260 domain containing protein.	132	180	119	426	400	369	143.6629	398.583467	2.774435617
Os09g0297900|AK107	Conserved hypothetical protein.	32	17	14	51	67	58	21.0389133	58.3523333	2.77354312
Os08g0390700|AK062	2-oxoglutarate-dependent oxygenase.	40	26	16	34	161	36	27.7690033	76.9748467	2.77197009
Os07g0130700|Os07g	Resistance protein candidate (Fragment).	9	37	28	61	97	46	24.5730313	68.10699	2.771615316
Os06g0289900|CI0007	UDP-glucuronosyl/UDP-glucosyltransferase family protein.	4335	7317	2702	16641	13205	9923	4784.47567	13256.3223	2.770694901
Os12g0420400|AK119	Photosystem I reaction center subunit XI, chloroplast precursor (PSI- L	54	35	32	41	259	36	40.4248567	111.974733	2.769947566
Os04g0414500|AK121	Hypothetical protein.	99	138	104	391	306	248	113.76331	315.063333	2.769463488
Os01g0731100|AK061	Conserved hypothetical protein.	74	155	98	291	371	244	109.03579	301.9449	2.769227425
Os10g0550900|AK121	Proline dehydrogenase domain containing protein.	653	615	677	1578	1877	1931	648.481	1795.16933	2.768268204
Os09g0240600|CI0697	(No Hit)	339	363	316	980	884	955	339.5011	939.685767	2.767843069
Os09g0559200|AK109	Hypothetical protein.	8	6	6	6	40	11	6.852683	18.9485703	2.765131604
Os01g0626400|AK108	WRKY transcription factor 34.	21	27	20	88	50	52	22.8473367	63.1618133	2.764515368
Os03g0117100|AK104	Peroxisomal biogenesis factor 11 family protein.	136	176	177	477	314	561	163.028533	450.562333	2.763702305

Os02g0526400|AY166	ERD1 protein, chloroplast precursor.	12	10	16	37	36	31	12.505627	34.53525	2.761576849
Os01g0350500|CI4448	(No Hit)	8	6	5	7	12	35	6.441754	17.788147	2.761382536
Os03g0339300|AK104	Peroxidase (EC 1.11.1.7).	12	15	8	7	22	68	11.6627353	32.1832437	2.759493613
Os06g0330400|Os06g	Sterol desaturase family protein.	124	51	33	188	239	144	69.04702	190.264467	2.755578252
Os01g0351200|Os01g	Poly [ADP-ribose] polymerase (EC 2.4.2.30) (PARP) (ADPRT) (NAD(+	25	24	21	20	164	10	23.4531767	64.6200433	2.755278922
Os06g0212500|AK071	Galactoside 2-alpha-L-fucosyltransferase (EC 2.4.1.69) (Xyloglucan al	8	11	5	6	12	50	8.17662667	22.5273583	2.755091953
Os03g0353300|AK111	Hypothetical protein.	7	8	5	13	34	9	6.88077433	18.956814	2.755040797
Os04g0252400|AK119	SNARE-interacting protein KEULE.	99	115	123	176	610	142	112.355433	309.484867	2.754516248
Os10g0461100|AK065	Transposase.	41	18	20	33	127	59	26.4744733	72.91616	2.754206253
osa-miR395i|Os08|NA|	miRNA	14	13	5	8	71	10	10.7586213	29.6275407	2.753841756
Os05g0586100|CI4450	(No Hit)	30	48	39	52	61	208	38.89764	107.106047	2.753535861
Os06g0212100|AK103	Xyloglucan fucosyltransferase family protein.	9	9	9	21	16	38	9.145534	25.1806167	2.753323826
Os03g0268600|AK069	Protein phosphatase type 2C.	224	273	252	772	589	697	249.4928	686.015067	2.749638734
Os01g0749000|AK107	Protein of unknown function DUF1264 family protein.	71	42	28	112	187	87	46.7742833	128.53074	2.747893305
Os01g0193400|AK069	Conserved hypothetical protein.	18	16	5	15	85	10	13.3024577	36.511303	2.744703566
Os10g0505500|AK062	Plant lipid transfer/seed storage/trypsin-alpha amylase inhibitor domai	8	6	5	6	13	35	6.543816	17.9576193	2.744212144
osa-miR169c|Os04|NA	miRNA	30	30	17	59	118	32	25.4842133	69.9327833	2.74416096
Os08g0190100|AK058	Oxalate oxidase-like protein or germin-like protein (Germin-like 8) (Ger	309	289	266	742	843	784	287.799067	789.694367	2.743908713
Os08g0189900|AF141	Oxalate oxidase-like protein or germin-like protein (Germin-like 8) (Ger	175	195	150	414	544	469	173.427	475.857867	2.743851111
Os06g0542600|CI4730	(No Hit)	10	9	9	8	59	11	9.39358967	25.7710047	2.74346715
Os11g0483000|CI5532	Cytochrome P450 family protein.	2304	2208	2086	5493	5231	7366	2199.206	6029.89567	2.741851226
Os06g0136600|AK104	Enolase 1 (EC 4.2.1.11) (2-phosphoglycerate dehydratase 1) (2-phosp	1077	1104	1077	2896	2863	3167	1086.13633	2975.51433	2.739540371
Os03g0128500|AB037	DNA polymerase delta small subunit (EC 2.7.7.7).	57	42	63	56	47	338	53.72859	147.17037	2.739144467
Os07g0664000|CI0114	Short-chain dehydrogenase/reductase SDR family protein.	29	42	38	79	144	75	36.3334467	99.3678367	2.734886056
Os06g0303600|AK062	Non-protein coding transcript, putative npRNA.	7	6	6	6	34	12	6.30182267	17.215677	2.731856783
Os04g0320700|AK119	Glucosyltransferase (Fragment).	813	947	901	2578	2274	2415	887.117533	2422.212	2.730429632
Os04g0320700|AK105	Glucosyltransferase (Fragment).	532	670	595	1707	1519	1678	599.0073	1634.75133	2.729100853
Os01g0686600|AK119	TPR-like domain containing protein.	12	13	6	6	20	57	10.078902	27.5059467	2.729061823
Os01g0235500|AK121	Conserved hypothetical protein.	9	6	6	8	38	10	6.88979233	18.8016813	2.728918438
Os04g0428500|AK109	Conserved hypothetical protein.	45	35	27	49	82	165	36.0753567	98.4224567	2.728246253
osa-miR167d|Os07|NA	miRNA	20	10	5	15	40	42	11.846395	32.3048667	2.726978686
Os07g0472500|AK066	Myosin heavy chain-like protein (Fragment).	8	9	6	6	46	10	7.65446167	20.8697667	2.726483922
Os02g0650900|AK103	Glutamate dehydrogenase 2 (EC 1.4.1.3) (GDH 2).	8	6	6	11	34	10	6.83058767	18.6214067	2.726179295
Os07g0522500|AK105	PDR6 ABC transporter.	312	398	459	1065	917	1205	389.6808	1061.88943	2.725023746
Os04g0320700|AK105	Glucosyltransferase (Fragment).	1150	1443	1358	3523	3518	3724	1317.04067	3588.331	2.724540776
Os09g0426100|AK105	Protein of unknown function DUF6 domain containing protein.	167	165	139	422	459	402	157.224867	428.0146	2.722308558
Os06g0313500|CI4288	Hypothetical protein.	24	26	26	36	90	79	25.0617033	68.2049133	2.721479559
Os02g0107800|CI2609	(No Hit)	44	45	33	106	129	97	40.7068967	110.715523	2.719822251
Os02g0249900|X15833	Glutelin type-B 1 precursor.	9	7	6	7	44	11	7.622532	20.7305687	2.719643377
Os08g0205700|AK062	Hypothetical protein.	23	20	15	62	79	17	19.3858333	52.6931067	2.718124404
Os08g0112300|AK070	Transferase family protein.	33	41	40	109	116	85	38.0179	103.32738	2.717861323

Os04g0227000|AK111	Protein kinase domain containing protein.	9	6	8	19	12	30	7.54094	20.4631267	2.713604228
Os06g0110000|AK069	Ent-kaurenoic acid oxidase 2 (EC 1.14.13.79) (AtKAO2) (Cytochrome I	744	855	883	2594	1926	2214	827.301	2244.72	2.713305073
Os04g0611800|AY347	Terpene synthase, metal-binding domain containing protein.	165	226	219	721	371	561	203.546367	550.924533	2.706629169
Os01g0549300|AK107	Homeodomain-like containing protein.	10	10	11	34	21	31	10.594757	28.6744667	2.70647705
Os03g0857400|AK062	Conserved hypothetical protein.	26	28	26	37	61	118	26.5045867	71.7106233	2.705592969
Os02g0517900|AK062	Conserved hypothetical protein.	14	8	9	16	55	13	10.325251	27.9196267	2.70401433
POsControl0044|art|NA	NONE	10	8	13	12	60	13	10.4439313	28.2300133	2.703006409
Os10g0177400|AK064	Pathogenicity protein PATH531-like protein.	95	75	61	76	488	61	77.2369	208.62512	2.701106854
Os01g0370900|CI4378	Glutathione-S-transferase 19E50.	16	7	5	48	17	10	9.26625567	25.0099567	2.699035896
Os11g0701300|CI0791	Chitinase (EC 3.2.1.14).	14	9	6	10	56	12	9.70065933	26.1710933	2.697867478
osa-miR395p|Os04|NA	miRNA	7	7	5	6	38	9	6.55176533	17.6752927	2.697790865
Os01g0525000|AK064	Conserved hypothetical protein.	35	35	16	31	88	114	28.83488	77.7782433	2.697366638
Os03g0437100|AK108	Zn-finger, C2H2 type domain containing protein.	71	54	51	176	128	173	58.93509	158.8733	2.69573356
Os02g0590200|CI4286	(No Hit)	37	37	18	38	169	41	30.7073267	82.7161067	2.693692862
osa-miR171h|Os04|NA	miRNA	51	35	24	26	241	30	36.6715233	98.7371933	2.692475915
Os08g0443800|AK110	CD9/CD37/CD63 antigen family protein.	9	35	35	101	56	57	26.3653197	70.9802933	2.69218406
Os10g0530300|Os10g	Glutathione S-transferase GST 22 (EC 2.5.1.18) (Fragment).	724	768	699	2108	1849	1939	730.315867	1965.418	2.691188963
Os03g0296700|AK067	Hypothetical protein.	51	37	37	62	222	53	41.77187	112.337697	2.689314524
Os01g0375400|Os01g	Dehydroquinate dehydratase/shikimate:NADP oxidoreductase (EC 4.2.	15	18	10	13	90	11	14.10772	37.9146467	2.687510573
Os04g0600500|AK105	Conserved hypothetical protein.	78	78	84	247	138	262	80.3047567	215.810333	2.68739166
Os01g0731100|AK106	Conserved hypothetical protein.	83	150	104	288	381	236	112.331693	301.8086	2.686762667
Os01g0389200|AK061	Protein of unknown function DUF679 family protein.	100	99	112	276	262	297	103.762693	278.584	2.684818513
Os03g0658800|AK104	Cytochrome P450 family protein.	48	52	34	221	52	87	44.8052667	120.25071	2.683852122
Os06g0662900|CI1161	Pollen allergen Lol p2 family protein.	8	7	5	6	40	10	6.90911467	18.5420867	2.683713842
Os04g0674700|AK106	Amp-binding protein.	665	760	579	2130	1498	1747	667.913067	1791.921	2.682865614
Os08g0189500|CI5453	Oxalate oxidase-like protein or germin-like protein (Germin-like 8) (Ger	273	342	292	682	925	828	302.6785	811.9919	2.682687736
Os02g0733900|AK111	Conserved hypothetical protein.	27	55	32	106	105	95	38.1524733	102.291293	2.681118271
Os12g0154800|CI4144	Germin-like protein precursor.	9	7	6	32	14	15	7.577972	20.3160767	2.680938471
Os03g0383900|AK069	Heavy metal transport/detoxification protein domain containing protein.	8	6	5	6	20	25	6.38473267	17.1161107	2.680787366
Os02g0749800|AK064	Mitochodrial transcription termination factor-related family protein.	22	40	21	17	48	155	27.35273	73.26137	2.678393345
Os11g0214700|Os11g	Conserved hypothetical protein.	9	7	6	7	31	20	7.19247267	19.2557477	2.67720832
PGmControl0001|AF03	NONE	13	8	5	13	46	10	8.60014733	23.0121867	2.675789818
Os10g0512100|CI1249	(No Hit)	17	15	8	32	53	23	13.384902	35.8151533	2.675787491
Os04g0611800|AY347	Terpene synthase, metal-binding domain containing protein.	197	226	245	747	410	629	222.717067	595.0996	2.671998194
Os11g0144900|AK120	Hypothetical protein.	281	376	402	957	735	1135	352.9938	942.139167	2.668996358
Os08g0457000|AK106	Conserved hypothetical protein.	9	10	6	32	22	12	8.32125033	22.1845467	2.66601121
osa-miR171a|Os06|NA	miRNA	41	26	29	28	76	154	32.1299367	85.6254833	2.664975167
Os04g0654400|AK107	Hypothetical protein.	21	29	14	51	94	26	21.2644233	56.6671867	2.664882361
Os09g0400400|CI1936	Cinnamyl alcohol dehydrogenase.	238	282	336	739	897	645	285.374533	760.342267	2.66436622
POsControl0044|art|NA	NONE	7	8	8	6	46	10	7.67268333	20.441628	2.66420848
Os08g0344700|Os08g	Retrotransposon gag protein family protein.	20	25	15	17	131	14	20.3469767	54.1775567	2.662683383

Os05g0513500|Os05g(	Ribosomal protein L33 family protein.	17	10	6	25	46	17	10.9350993	29.1146267	2.662493113
Os12g0204700|AK102	Hypothetical protein.	36	22	14	14	168	9	23.95376	63.7402693	2.66097136
PAtControl0001|U4334	NONE	23	15	8	16	60	46	15.327224	40.78057	2.660662492
Os10g0562900|AK107	Pathogenesis-related transcriptional factor and ERF domain containing	176	177	186	429	479	524	179.530833	477.472367	2.659556344
Os01g0737100|AK108	Conserved hypothetical protein.	9	11	8	23	33	16	9.10352767	24.1837633	2.656526593
Os01g0232000|CI4716	Aquaglyceroporin (Tonoplast intrinsic protein (Tipa)).	12	9	16	12	13	72	12.2539537	32.55185	2.656436517
Os09g0530000|AK062	Rhodanese-like domain containing protein.	8	6	6	11	31	12	6.71468867	17.8342967	2.656012446
Os05g0217800|AK109	BURP domain containing protein.	8	6	5	11	30	10	6.36911267	16.9160547	2.655951551
Os02g0818000|AK071	CBS domain containing protein.	8	15	6	34	21	22	9.61328733	25.5253733	2.655217976
Os04g0101700|AK069	Engulfment and cell motility, ELM domain containing protein.	31	14	16	27	112	24	20.4602933	54.2433867	2.65115391
Os07g0133500|AK106	Protein of unknown function DUF1005 family protein.	9	8	6	7	15	41	7.81351767	20.699669	2.649212542
Os07g0409200|AK119	Xylulose kinase (EC 2.7.1.17).	8	6	5	6	34	10	6.315743	16.7263487	2.648358026
Os10g0364800|AK106	Non-protein coding transcript, unclassifiable transcript.	79	71	65	70	187	310	71.49339	189.202637	2.646435379
Os05g0131100|AK068	Conserved hypothetical protein.	67	59	74	218	139	175	67.0156667	177.343233	2.646295145
Os11g0514400|Os11g(	BRASSINOSTEROID INSENSITIVE 1-associated receptor kinase 1 pr	19	11	6	33	50	15	12.3436527	32.6639567	2.646214824
Os10g0142600|CI1135	Protein kinase domain containing protein.	18	25	24	49	79	50	22.4220967	59.3110133	2.645203712
osa-miR166d|Os02|NA	miRNA	31	32	19	20	92	103	27.03372	71.4771433	2.643999543
POsControl0023|genon	NONE	8	6	6	7	35	10	6.54002067	17.2896067	2.64366239
Os03g0800000|AK068	Conserved hypothetical protein.	382	263	347	776	856	992	330.876267	874.6931	2.64356555
Os07g0661400|AK107	Conserved hypothetical protein.	84	66	39	52	405	42	63.0262833	166.60379	2.643401787
Os10g0490900|Os10g(	NtPRp27.	408	521	562	1397	990	1556	497.355433	1314.5948	2.643169677
Os03g0187800|AK105	Protein of unknown function DUF250 domain containing protein.	502	387	365	1050	825	1439	417.9907	1104.45743	2.642301452
Os07g0577600|AK070	Lhca2 protein.	17	14	6	15	40	43	12.2399137	32.3272433	2.64113328
osa-miR171i|Os03|NA|	miRNA	7	6	5	19	12	18	6.10277533	16.11216	2.640136515
Os04g0468600|AK067	Conserved hypothetical protein.	27	35	27	46	157	35	29.9857833	79.14786	2.639512836
Os01g0714600|AK059	Conserved hypothetical protein.	1026	1369	1283	3627	2667	3410	1226.132	3234.66067	2.638101499
Os06g0275800|AK120	Amino acid/polyamine transporter II family protein.	9	7	6	8	39	11	7.31043133	19.2810303	2.637468222
Os08g0327200|AK069	Glycoside hydrolase, family 28 protein.	52	36	18	24	240	16	35.3821567	93.2969	2.636834744
Os01g0764900|AK105	Formamidase-like protein.	14	8	6	21	42	11	9.35217433	24.65738	2.636539816
Os08g0231100|AK065	Hypothetical protein.	12	6	5	17	33	9	7.50205367	19.777554	2.636285326
POsControl0007|genon	NONE	9	7	6	6	39	11	7.137371	18.8114133	2.635622183
Os04g0465600|AK059	Bet v I allergen family protein.	10	8	10	42	17	13	9.20783767	24.24045	2.632588766
Os01g0955100|AK062	Regulator of gene silencing.	593	781	697	2019	1619	1814	690.423567	1817.24967	2.632079428
Os10g0536400|AK061	Conserved hypothetical protein.	2765	2287	2441	5834	6302	7562	2497.46033	6565.83	2.629002716
Os01g0360100|AK062	Conserved hypothetical protein.	41	35	22	48	93	117	32.77283	86.1317533	2.628145123
PGmControl0001|AF03	NONE	14	10	7	18	53	12	10.5250143	27.6459433	2.626689376
Os04g0647900|AK068	Leucine-rich repeat, typical subtype containing protein.	56	54	48	136	118	161	52.7297767	138.465633	2.625947654
Os06g0288300|Os06g(	UDP-glucuronosyl/UDP-glucosyltransferase family protein.	19	18	16	26	42	71	17.67016	46.3968067	2.625715142
Os06g0592400|AK062	Cytosolic aldehyde dehydrogenase RF2C.	667	801	609	2256	1447	1752	692.546133	1818.06533	2.625190216
osa-miR395e|Os04|NA	miRNA	9	8	8	7	14	45	8.372893	21.9668953	2.62357292
Os04g0288500|AK062	Protein kinase domain containing protein.	24	17	12	24	79	37	17.7875	46.66234	2.623321996

Os05g0217800|AK109	BURP domain containing protein.	8	6	5	23	18	10	6.41673233	16.8281497	2.622541941
Os08g0509100|AF095	Lipoxygenase, chloroplast precursor (EC 1.13.11.12).	9	8	6	21	28	13	7.79216633	20.4312733	2.622027362
Os03g0117100|AK104	Peroxisomal biogenesis factor 11 family protein.	123	130	144	372	242	425	132.1666	346.5324	2.621936253
Os06g0568600|AY660	Ent-kaurene oxidase (EC 1.14.13.78) (AtKO1) (Cytochrome P450 701	205	178	218	480	560	536	200.486	525.455533	2.620908858
osa-miR172a|Os09|NA	miRNA	7	6	5	6	32	9	5.96959967	15.642615	2.620379234
Os07g0539100|AK062	Glycoside hydrolase, family 17 protein.	1914	2872	1810	7073	4245	5960	2198.69267	5759.39833	2.619464931
Os11g0144500|AK067	Zn-finger, RING domain containing protein.	29	24	11	22	82	63	21.2192867	55.5702733	2.618856807
Os10g0527800|AK063	Tau class GST protein 3.	177	251	212	547	490	637	213.264433	558.160233	2.617221374
Os02g0570400|AK068	Terpene synthase, metal-binding domain containing protein.	73	57	36	90	217	130	55.6790567	145.71789	2.617104145
Os09g0242300|Os09g	WD-repeat protein.	8	6	5	6	35	10	6.59058633	17.2451943	2.616640381
POsControl0048|art|NA	NONE	8	6	5	6	12	31	6.25360067	16.3626037	2.616509198
Os02g0309000|Os02g	Integrase, catalytic region domain containing protein.	7	13	5	6	51	10	8.40618133	21.9878487	2.615676226
Os07g0539100|AK067	Glycoside hydrolase, family 17 protein.	1988	2859	1885	7120	4540	5943	2243.99	5867.80767	2.614899205
osa-miR171h|Os04|NA	miRNA	33	43	45	141	88	91	40.7012033	106.425757	2.614806147
Os01g0609300|AK058	PDR-like ABC transporter (PDR3 ABC transporter).	13	25	26	53	58	54	21.0368533	55.0014233	2.614527109
Os08g0188900|Os08g	Germin-like protein precursor.	59	65	67	145	163	189	63.4687567	165.8058	2.612400316
Os01g0106900|AK059	1-deoxy-D-xylulose 5-phosphate reductoisomerase, chloroplast precur	1498	1702	1910	5102	3295	4946	1702.99933	4447.57567	2.611613275
PZmControl0002|X548	NONE	12	7	6	17	39	11	8.51270733	22.2305633	2.611456316
Os10g0392400|AK061	ZIM domain containing protein.	228	246	272	576	426	944	248.5183	648.957567	2.611306961
Os04g0171600|AK070	Cytochrome P450 79A1 (EC 1.14.13.41) (Tyrosine N-monooxygenase)	28	8	7	10	90	13	14.341427	37.4296133	2.609894631
Os06g0115600|CI5392	Conserved hypothetical protein.	106	133	133	382	240	348	123.973233	323.395833	2.608594006
Os12g0236800|CI4134	(No Hit)	8	6	6	8	34	10	6.72355967	17.5255917	2.606594205
Os10g0485900|Os10g	Conserved hypothetical protein.	170	167	162	444	428	430	166.468167	433.8594	2.606260456
Os05g0280700|AK100	Resistance protein candidate (Fragment).	10	8	7	10	42	13	8.30095	21.6332287	2.606114802
Os01g0149500|AK100	Resistance gene analog PIC23 (Fragment).	52	59	27	41	291	30	46.3841067	120.777707	2.603859713
Os06g0551500|AK065	Conserved hypothetical protein.	27	21	17	23	137	10	21.72669	56.5597767	2.603239456
Os04g0353600|CI3318	Male sterility protein family protein.	8	6	5	6	35	10	6.55434833	17.0607707	2.602969784
Os12g0415400|Os12g	Histone H3.	8	6	6	6	13	33	6.62187133	17.234155	2.602610974
POsControl0046|art|NA	NONE	10	10	6	7	50	11	8.68099	22.5877333	2.601976656
Os09g0309200|AK109	Hypothetical protein.	46	30	24	98	104	59	33.40663	86.9229667	2.601967534
Os02g0569400|AK069	Cytochrome P450 family protein.	61	53	45	50	297	66	52.98432	137.85343	2.601777847
Os07g0532600|AK058	Non-protein coding transcript, uncharacterized transcript.	8	6	5	26	12	13	6.50556933	16.9241	2.601478692
Os06g0570000|Os06g	RNA-directed DNA polymerase (Reverse transcriptase) domain contai	8	6	6	11	29	10	6.43228633	16.730626	2.601038749
Os12g0499200|Os12g	Hypothetical protein.	66	56	48	144	156	142	56.7044033	147.4628	2.600552891
Os01g0868200|AK119	Zn-finger, DHHC type domain containing protein.	9	7	6	8	37	11	7.28118933	18.9317913	2.60009601
Os05g0561200|Os05g	Bipartite response regulator, C-terminal effector domain containing pro	7	6	5	6	32	9	5.991221	15.5755937	2.599736125
Os05g0401100|AK067	Protein of unknown function DUF477 family protein.	39	23	18	15	182	10	26.6313533	69.1947767	2.598244851
Os11g0683800|AK070	Pectinesterase family protein.	8	6	7	33	13	11	7.335276	19.0580667	2.598139002
Os01g0241400|AK107	Glutaredoxin domain containing protein.	57	43	30	61	169	107	43.35473	112.563043	2.596326706
Os01g0678100|AK099	Conserved hypothetical protein.	23	21	17	42	71	44	20.13249	52.2664333	2.596123646
POsControl0028|rando	NONE	15	9	5	13	52	10	9.62545633	24.9592723	2.593048212

Os06g0685200|CI5084	(No Hit)	9	6	5	31	12	10	6.64744	17.236982	2.593025586
POsControl0025|rando	NONE	9	7	6	7	40	11	7.48992467	19.4110757	2.591624954
Os01g0106900|AK099	1-deoxy-D-xylulose 5-phosphate reductoisomerase, chloroplast precur	1354	1485	1771	4303	3012	4631	1536.49233	3981.912	2.591559954
Os04g0677400|AK069	Conserved hypothetical protein.	23	31	30	41	83	94	27.9858133	72.51495	2.591132483
Os07g0643700|AK109	Esterase/lipase/thioesterase domain containing protein.	53	31	18	48	60	157	34.0797433	88.2891333	2.590663095
Os10g0144800|CI2531	(No Hit)	8	6	5	6	33	10	6.262604	16.214662	2.589124588
Os12g0199000|CI3384	Beta-glucosidase aggregating factor.	8	6	5	6	12	31	6.225967	16.1134543	2.58810468
Os03g0319000|AK070	Hypothetical protein.	193	231	259	520	591	653	227.358133	588.107067	2.586699046
Os06g0119300|AK067	Protein of unknown function DUF594 family protein.	14	18	18	108	12	10	16.8402267	43.54612	2.585839304
Os04g0528400|AK107	Hus1-like protein family protein.	21	14	13	26	46	51	15.8692	41.0341467	2.58577286
Os01g0219500|AK106	Plant lipid transfer protein/Par allergen family protein.	8	6	6	13	29	10	6.70546167	17.3257333	2.583824082
Os03g0722800|AK121	Cyclin-like F-box domain containing protein.	11	12	12	68	13	10	11.71419	30.22823	2.580479743
Os10g0542100|AY572	EC protein I/II (Zinc-metallothionein class II).	236	199	174	547	554	469	202.927467	523.529	2.579882401
Os05g0322900|AK063	WRKY transcription factor 45.	340	342	350	985	784	890	343.7191	886.6831	2.579673635
Os11g0474800|AK108	Terpene synthase-like domain containing protein.	8	8	5	6	39	10	7.04416033	18.1501393	2.57662212
Os04g0468600|AK102	Conserved hypothetical protein.	435	449	559	1258	1148	1310	481.049633	1238.56733	2.574718381
Os01g0106900|AF3672	1-deoxy-D-xylulose 5-phosphate reductoisomerase, chloroplast precur	1552	1681	1944	4946	3301	5075	1725.55067	4440.73	2.573514696
Os04g0578600|AK068	Ferric reductase-like transmembrane component family protein.	10	7	6	7	40	11	7.50828133	19.3189683	2.573021371
Os04g0611800|AY347	Terpene synthase, metal-binding domain containing protein.	198	210	221	642	447	529	209.605667	539.319467	2.573019495
Os11g0192500|CI0794	(No Hit)	8	6	6	30	13	10	6.78780867	17.4510067	2.570933791
Os11g0454800|CI5179	(No Hit)	7	11	8	6	23	40	8.930975	22.9509653	2.569816323
Os06g0601500|CI4308	cAMP response element binding (CREB) protein family protein.	9	7	6	6	38	11	7.090482	18.2198163	2.569616048
Os02g0624300|AK112	MYB1 protein.	965	1110	1116	2720	2177	3302	1063.69667	2732.82133	2.569173543
Os01g0578700|Os01g	Hypothetical protein.	10	12	7	25	35	16	9.79315167	25.1272167	2.565794702
Os07g0166800|AK070	Rad6 (Ubiquitin carrier protein).	82	54	37	54	354	35	57.4890533	147.46974	2.565179481
Os02g0212300|Os02g	Hypothetical protein.	69	50	50	86	134	213	56.1684	144.011877	2.563930549
Os10g0529700|AK064	Glutathione-S-transferase 2.	9	7	6	6	13	35	7.10573467	18.2165523	2.563640945
Os02g0610700|AK100	Conserved hypothetical protein.	27	38	28	180	36	21	30.9514767	79.34552	2.563545541
Os09g0571200|AK062	C2 domain containing protein.	2131	2743	2499	6337	5431	7128	2457.28933	6298.55533	2.563212743
osa-miR395c|Os04|NA	miRNA	17	15	6	7	66	23	12.5731567	32.2006673	2.56106467
PGmControl0003|AF03	NONE	16	11	5	22	50	10	10.6786173	27.3477177	2.560979274
Os03g0740900|AK062	Non-protein coding transcript, unclassifiable transcript.	106	109	106	282	272	269	107.1469	274.2604	2.559667149
Os04g0252000|Os04g	Pol protein (Fragment).	9	7	6	8	35	10	7.024755	17.96919	2.557981026
Os09g0485600|AK063	Zinc finger-like protein (WIP2 protein).	15	7	6	11	47	11	9.051631	23.12396	2.554673296
Os11g0694100|C2261	Protein kinase domain containing protein.	546	503	546	1211	1371	1490	531.4944	1357.58633	2.554281538
Os09g0457800|AK101	Alpha-amylase isozyme 3C precursor (EC 3.2.1.1) (1,4-alpha-D-glucan	40	30	7	18	170	10	25.786227	65.8590467	2.554039669
Os11g0661600|AK108	Peroxidase precursor (EC 1.11.1.7).	8	6	6	8	33	10	6.67756067	17.0457967	2.552698136
Os04g0516900|AK108	Conserved hypothetical protein.	9	7	7	18	30	11	7.80910067	19.9335167	2.552600807
Os08g0496800|AK120	RAFTIN1a protein (RAFTIN1a anther protein).	42	35	32	77	129	71	36.24048	92.38706	2.549278045
Os11g0683800|AK100	Pectinesterase family protein.	8	6	7	27	16	10	6.986107	17.8087367	2.549164601
Os09g0569800|Os09g	Protein kinase domain containing protein.	896	1106	1190	2849	2191	3093	1064.02983	2710.94967	2.547813587

POsControl0024|genon	NONE	9	6	5	6	35	10	6.62284467	16.8642043	2.546368695
Os02g0789700|AK070(	Conserved hypothetical protein.	7	6	5	6	30	10	5.962951	15.1831637	2.546249947
Os09g0572000|CI4303	(No Hit)	90	95	89	263	260	174	91.3713367	232.626667	2.545947943
Os04g0468600|AK109:	Conserved hypothetical protein.	1509	1535	1842	3248	4235	4952	1628.478	4145.10233	2.5453843
Os03g0335200|AK105:	WRKY DNA binding protein.	146	143	147	342	388	381	145.444767	370.151233	2.544960825
osa-miR159f|Os01|NA|	miRNA	9	7	6	15	28	10	7.08159867	18.02084	2.544741781
Os07g0664100|CI5605	Glucose/ribitol dehydrogenase family protein.	36	28	22	67	90	60	28.3833933	72.20654	2.543971369
Os05g0428000|Os05g(	Hypothetical protein.	24	22	10	25	82	35	18.5643333	47.2126367	2.543190527
Os03g0310600|AK109	Plant protein of unknown function family protein.	163	155	169	400	396	442	162.4139	412.650067	2.540731222
Os01g0206800|AY332	Protein kinase domain containing protein.	55	60	57	65	285	87	57.4308367	145.81384	2.538946818
Os08g0352100|CI0005	Conserved hypothetical protein.	38	40	23	92	112	54	33.84131	85.9193133	2.538888516
Os08g0459700|CI0284	(No Hit)	109	94	88	241	274	225	97.2701667	246.9542	2.538848328
Os02g0513700|CI4481	Protein of unknown function DUF659 domain containing protein.	10	9	7	8	45	12	8.533546	21.657902	2.53797214
Os07g0565800|AK062	Conserved hypothetical protein.	51	27	38	131	75	88	38.6277833	97.84943	2.533136037
Os01g0944900|AK058	Beta-1,3-glucanase precursor.	11	6	10	16	32	21	9.01430567	22.8219	2.531742415
Os11g0475000|AK120	Hypothetical protein.	10	8	5	9	39	10	7.627588	19.310451	2.531658894
osa-miR395f|Os04|NA|	miRNA	12	21	10	61	37	12	14.4919913	36.6871233	2.531544664
POsControl0041|art|NA	NONE	9	12	6	7	22	40	9.152949	23.1689007	2.531304464
Os11g0302800|AK064	Protein of unknown function DUF724 family protein.	103	73	93	235	196	251	89.8385667	227.189767	2.528866778
Os10g0191000|Os10g(	RNA-directed DNA polymerase (Reverse transcriptase) domain contair	10	7	12	48	15	13	9.993433	25.2665467	2.528315011
Os08g0565200|AY339:	Pathogenesis-related transcriptional factor and ERF domain containing	29	26	17	24	66	94	24.1872367	61.1489133	2.528147972
Os12g0109700|AK058(	Hypothetical protein.	99	154	136	378	301	304	129.526497	327.454367	2.528087882
Os04g0632500|AK072	S-locus receptor-like kinase RLK11.	64	43	29	34	278	31	45.2724267	114.42266	2.527424934
osa-miR399g|Os02|NA	miRNA	42	37	29	163	102	9	36.2187467	91.512088	2.526649772
Os04g0313300|AK121	Conserved hypothetical protein.	125	136	124	423	275	275	128.349167	324.199367	2.525917192
Os04g0632100|AK065:	S-locus receptor-like kinase RLK13.	9	8	6	13	32	10	7.35021567	18.5578833	2.524808002
Os05g0421600|AK107	Conserved hypothetical protein.	262	307	225	749	670	584	264.414133	667.550733	2.524640892
Os04g0497000|AK109:	(+)-pulegone reductase.	157	159	348	587	488	602	221.4264	558.991733	2.524503552
Os04g0454900|AK073	Hypothetical protein.	32	32	31	82	79	79	31.78064	80.2120833	2.523929138
Os02g0456000|AK119	Cysteine-rich extensin-like protein-1.	10	13	10	14	47	22	10.9861607	27.7278367	2.523887781
Os06g0635300|CI2602	AB-hydrolase associated lipase region domain containing protein.	10	12	6	7	54	12	9.531373	24.0546803	2.523737171
Os11g0551300|CB628	Disease resistance protein family protein.	7	6	5	6	12	30	6.231785	15.7221573	2.522897907
Os01g0364800|AU162	EGF-like calcium-binding domain containing protein.	29	42	61	123	78	131	44.01451	110.88478	2.519277847
Os02g0127300|AK107	Non-protein coding transcript, uncharacterized transcript.	32	19	13	29	122	10	21.2643167	53.5354367	2.517618483
Os06g0139400|AK110	Conserved hypothetical protein.	20	14	7	11	80	13	13.844571	34.85053	2.517270488
Os11g0490600|AK104	Hypothetical protein.	15	34	20	66	44	64	22.9963733	57.8852	2.517144732
Os01g0202900|Os01g(	Hypothetical protein.	22	19	9	9	109	10	16.8923157	42.5162187	2.516897002
Os01g0554100|AK060	RNA-directed DNA polymerase (Reverse transcriptase) domain contair	11	9	7	13	42	12	8.932011	22.4624267	2.514822996
Os05g0166600|Os05g(	Protein kinase domain containing protein.	9	6	5	6	36	10	6.87024733	17.276828	2.51473159
Os01g0248300|Os01g(	Conserved hypothetical protein.	518	447	519	1453	1033	1244	494.417733	1243.162	2.514396059
Os09g0441700|CI4215	(No Hit)	21	11	8	11	45	45	13.2996857	33.42119	2.512930819

Os06g0157700|AB052!	SP3D.	12	14	6	9	59	11	10.4225047	26.187337	2.512576184
Os08g0406600|AK064	MATH domain containing protein.	8	6	5	6	32	10	6.42230767	16.129198	2.511433403
Os07g0429300|CI1219	(No Hit)	8	11	5	6	12	42	7.86687067	19.7565963	2.511366612
Os07g0178800|AF017	Early light-inducible protein.	27	8	7	17	74	12	13.733779	34.4627067	2.509338957
Os07g0558400|AK104	Chlorophyll a/b-binding protein CP29 precursor.	50	36	16	32	198	26	34.0390167	85.4108633	2.50920478
Os11g0182900|Os11g	Ankyrin repeat containing protein.	8	6	5	6	32	10	6.466699	16.2225163	2.508624003
Os09g0572000|CI4303	(No Hit)	93	92	91	262	258	173	92.2052067	231.2219	2.507688105
Os11g0257800|AK069	Conserved hypothetical protein.	27	11	5	8	92	10	14.626443	36.6615743	2.506527003
Os01g0975900|AB114	Tonoplast membrane integral protein ZmTIP1-2.	1321	1382	1258	3837	2572	3520	1320.44	3309.52933	2.506383731
Os09g0364400|CI1881	Conserved hypothetical protein.	372	563	478	1241	1064	1238	471.184733	1180.96867	2.506381432
Os02g0528000|Os02g	Hypothetical protein.	8	9	7	23	27	10	7.94151267	19.89996	2.505814803
Os01g0975900|AK111	Tonoplast membrane integral protein ZmTIP1-2.	1658	1653	1558	4351	3475	4373	1622.73233	4066.26467	2.505813549
Os12g0443000|Os12g	Cytochrome P450-like protein.	121	101	76	182	353	209	99.0339167	248.013933	2.50433328
Os11g0227100|Os11g	Disease resistance protein family protein.	7	6	5	9	11	24	5.92409833	14.8330627	2.503851528
osa-miR439e|Os08|NA	miRNA	10	9	6	7	43	11	8.05635467	20.1707383	2.503705357
Os04g0105400|AK101	Conserved hypothetical protein.	4059	4826	4667	11604	10848	11435	4517.354	11295.5833	2.500486642
osa-miR319b|Os01|NA	miRNA	16	14	15	22	75	16	15.08952	37.7244367	2.500042193
Os12g0141100|AK102	Phosphatidylinositol-4-phosphate 5-kinase family protein.	123	92	78	83	583	65	97.42527	243.505167	2.499404586
Os01g0373700|AK107	Conserved hypothetical protein.	34	25	15	133	36	16	24.7864167	61.92379	2.49829537
Os01g0130400|AK102	Alpha-xylosidase precursor (F24J5.20 protein) (At1g68560/F24J5_10).	13	8	5	17	35	13	8.65303467	21.60541	2.496859291
Os04g0590600|CB628	Hypothetical protein.	20	19	16	89	34	14	18.2230267	45.4998567	2.496833128
POsControl0041|art|NA	NONE	10	8	7	8	29	23	8.01911667	20.022058	2.49679096
osa-miR162b|Os04|NA	miRNA	10	15	7	8	60	13	10.826922	27.0270493	2.496281892
Os07g0214100|X66257	Seed allergenic protein RA17 precursor.	9	26	6	7	86	11	13.8995533	34.6856197	2.495448511
POsControl0031|rando	NONE	15	13	5	8	64	9	10.8544473	27.071284	2.494026934
POsControl0024|genon	NONE	12	7	7	8	45	11	8.460589	21.08959	2.492685793
Os02g0245200|AK103	Hypothetical protein.	34	46	38	49	54	192	39.4343533	98.24433	2.491338686
POsControl0039|art|NA	NONE	8	6	5	6	24	18	6.42865033	16.0109227	2.490557401
Os08g0263600|Os08g	Hypothetical protein.	44	46	23	25	246	10	37.64425	93.738021	2.490101968
Os12g0595600|CI2614	(No Hit)	13	8	5	13	42	10	8.70354167	21.66878	2.489650861
Os07g0580700|AK119	Hypothetical protein.	91	65	71	67	125	371	75.4141333	187.706163	2.489005111
Os10g0488300|AK100	Vps52/Sac2 family protein.	10	12	6	13	44	11	9.15726067	22.7761167	2.487219431
Os01g0247500|AK065	Protein kinase domain containing protein.	55	70	31	176	118	91	51.8018967	128.755007	2.485526881
Os01g0826400|AY676	WRKY transcription factor 24.	258	231	210	552	632	553	232.9401	578.8782	2.485094666
Os07g0108900|AF058	MADS15 protein.	7	8	5	6	37	10	6.987993	17.3637533	2.48479833
Os01g0771000|AK069	Protein of unknown function DUF567 family protein.	49	40	29	87	122	84	39.35743	97.7939067	2.484763529
Os01g0618000|AK064	Exonuclease domain containing protein.	43	53	46	137	91	122	46.9814267	116.736843	2.484744539
Os01g0935200|CI4806	(No Hit)	8	8	6	6	36	10	7.09087133	17.6181677	2.484626619
Os03g0658800|AK065	Cytochrome P450 family protein.	69	70	50	244	65	158	62.83511	155.98508	2.482450974
Os04g0302300|AK072	Conserved hypothetical protein.	92	108	109	174	127	464	102.84558	255.139233	2.480799207
osa-miR395k|Os08|NA	miRNA	8	6	5	26	12	10	6.44015867	15.97437	2.480431124

Os08g0210500|AK106	Conserved hypothetical protein.	15	7	6	6	51	11	9.23656167	22.90666	2.4799986
Os04g0244900|Os04g	Conserved hypothetical protein.	79	70	35	46	179	232	61.37313	152.19966	2.479907086
Os06g0542600|CI4730	(No Hit)	10	15	11	9	66	13	11.8596243	29.4036223	2.479304699
Os05g0217800|AK106	BURP domain containing protein.	133	118	102	320	294	262	117.7226	291.7893	2.47861753
Os02g0181300|AK058	WRKY transcription factor 71 (Transcription factor WRKY09).	2057	2016	1888	4864	4043	5862	1986.84533	4922.67867	2.477635568
Os08g0386200|AK111	WRKY transcription factor 69.	3311	3525	3500	8731	7481	9388	3445.25267	8533.17033	2.476790865
Os08g0148500|CI5355	(No Hit)	10	7	6	7	15	36	7.816428	19.3579787	2.476576087
Os01g0108400|AU096	Basic helix-loop-helix dimerisation region bHLH domain containing prot	7	7	5	6	32	10	6.40297633	15.850511	2.475491111
Os04g0480200|AK109	Conserved hypothetical protein.	98	100	101	236	300	204	99.69388	246.624933	2.473822198
Os02g0538700|AK109	Harpin-induced 1 domain containing protein.	36	34	26	38	133	67	32.15028	79.4737967	2.471947263
Os04g0317800|AK111	Pollen allergen Lol p2 family protein.	8	6	5	6	12	29	6.359744	15.717139	2.471347746
POsControl0030|rando	NONE	26	12	6	18	81	10	14.8038603	36.57236	2.470461027
Os11g0175700|AB071	Transcription factor PCF3 (Fragment).	9	7	6	6	36	11	7.21044033	17.80501	2.469337402
Os03g0199200|AK109	Hypothetical protein.	10	7	7	7	40	12	7.92444267	19.549161	2.466944594
Os04g0340100|Os04g	Protein kinase-like domain containing protein.	693	788	811	2027	1496	2131	764.0646	1884.79767	2.466804072
Os04g0644400|AK061	Proline-rich-like protein.	9	7	6	6	35	11	7.09031033	17.4862573	2.466218898
Os01g0874800|AK103	5'-3' exonuclease domain containing protein.	9	11	6	13	38	11	8.36949667	20.6406967	2.466181359
Os03g0323300|Os03g	Beta-glucosidase.	19	13	9	20	72	10	13.7937793	34.0160367	2.466041818
Os10g0458600|CI4425	(No Hit)	17	7	14	6	77	11	12.8032373	31.5596783	2.464976436
Os03g0115500|AK119	Pyridoxamine 5'-phosphate oxidase-related domain containing protein.	76	103	105	142	88	469	94.6383667	233.275093	2.46491039
POsControl0034|rando	NONE	8	6	5	6	32	10	6.43018033	15.8496217	2.464879808
Os06g0287700|AK067	NBS-LRR disease resistance protein homologue.	59	63	43	130	179	97	54.9274767	135.3496	2.464151063
Os11g0229500|AB019	Disease resistance protein family protein.	50	67	52	141	96	179	56.33103	138.80024	2.464010333
Os04g0607600|AK120	Cation transporter family protein.	17	9	10	35	43	10	12.0255443	29.59869	2.461318106
Os11g0469900|Os11g	Cytochrome P450 family protein.	8	6	5	7	24	18	6.51449867	16.0298617	2.46064394
osa-miR159b|Os01|NA	miRNA	8	6	6	6	33	11	6.75102733	16.6027493	2.45929227
Os04g0390700|AK107	Short-chain dehydrogenase/reductase SDR family protein.	98	71	45	59	423	46	71.56797	175.96583	2.458723225
Os03g0190100|AK061	UbiA prenyltransferase family protein.	147	206	214	563	352	477	188.6638	463.830867	2.458504847
Os09g0547500|AK062	Lysine decarboxylase-like protein.	174	177	186	383	542	396	179.1117	440.340533	2.45846884
Os10g0490800|Os10g	Plant Basic Secretory Protein family protein.	1463	2257	2118	4436	3915	5999	1945.78533	4783.21567	2.45824428
osa-miR166l|Os09|NA|	miRNA	13	11	7	15	53	10	10.4717337	25.7413367	2.458173354
Os09g0410500|Os09g	TCP transcription factor family protein.	7	16	10	7	63	9	10.8357447	26.627967	2.457419201
Os12g0582000|Os12g	Cytochrome P450 family protein.	692	366	532	1008	889	2011	530.098467	1302.55447	2.457193425
Os11g0423300|AK064	Hypothetical protein.	66	43	46	57	283	40	51.5537167	126.643637	2.456537469
Os05g0530400|AY344	Heat shock factor protein 1 (HSF 1) (Heat shock transcription factor 1)	121	157	171	373	332	396	149.529033	367.1784	2.455565931
Os11g0128900|Os11g	Hypothetical protein.	35	28	31	42	52	136	31.2887233	76.7640533	2.453409572
Os12g0509300|AK108	Hypothetical protein.	8	7	5	10	28	10	6.57193933	16.11434	2.451991594
Os03g0734500|AK108	Conserved hypothetical protein.	29	18	8	20	88	28	18.4827033	45.3153867	2.451772657
Os02g0453600|AK064	RmlC-like cupin family protein.	42	45	42	29	96	191	43.0369	105.43061	2.449772405
Os07g0410300|AK108	Conserved hypothetical protein.	1085	1234	1319	3460	2912	2532	1212.725	2968.09367	2.447458135
Os02g0103300|AK069	Plant invertase/pectin methylesterase inhibitor domain containing prote	29	15	24	19	94	52	22.47923	54.9797633	2.445802785

Os01g0975900|AK111	Tonoplast membrane integral protein ZmTIP1-2.	69	61	70	171	146	174	66.92078	163.670767	2.445739076
Os01g0956500|AK072	Conserved hypothetical protein.	8	6	8	24	12	19	7.51901667	18.3813667	2.444650342
Os02g0127200|CI1718	Conserved hypothetical protein.	11	12	10	25	32	24	11.0651827	27.0479467	2.444419354
Os07g0550500|Os07g	S-locus glycoprotein family protein.	9	7	12	44	13	11	9.341681	22.8206967	2.44288974
Os11g0581700|CI5200	(No Hit)	21	28	23	24	133	19	23.98014	58.5663133	2.442284046
Os03g0234500|CI4257	Haem peroxidase, plant/fungal/bacterial family protein.	54	68	63	186	103	163	61.63473	150.515633	2.442058776
Os03g0679700|AK120	Thiamine biosynthesis protein thiC.	8	7	6	7	13	31	6.97298133	17.024845	2.441544611
Os02g0554100|AK060	UVB-resistance protein UVR8.	8	10	5	9	36	12	7.81308967	19.0687823	2.440619927
Os03g0856700|AK099	Ga20 oxidase (Fragment).	1566	2122	1994	5381	3912	4563	1894.22733	4618.70067	2.438303252
Os10g0395000|AK121	Protein kinase domain containing protein.	31	31	34	152	57	25	31.9280767	77.8441033	2.438108131
Os02g0518000|AK068	C2 calcium/lipid-binding region, CaLB domain containing protein.	11	13	14	43	23	27	12.7397467	31.0509833	2.437331302
Os01g0529800|AK101	Very-long-chain fatty acid condensing enzyme CUT1 (Very-long-chain	47	83	34	129	132	137	54.5076767	132.806633	2.436475767
Os08g0553800|AK073	Isoflavone reductase family protein.	39	44	34	61	84	142	39.2857767	95.7042567	2.436104483
Os08g0518400|Os08g	Protein kinase family protein.	9	15	7	34	30	10	10.0433947	24.4572117	2.435153898
Os03g0794200|CI4229	GCN5-related N-acetyltransferase domain containing protein.	37	52	57	143	71	143	48.9282933	119.104617	2.434268775
Os09g0341500|AK073	Cyclin-like F-box domain containing protein.	169	184	189	420	420	478	180.586533	439.527267	2.433887281
Os09g0241100|AK099	WD40-like domain containing protein.	160	131	172	168	790	172	154.719933	376.452167	2.433120016
Os01g0564300|AK064	Peptidylprolyl isomerase, FKBP-type domain containing protein.	2127	2789	3424	7429	4966	7894	2780.07733	6763.138	2.432715781
Os11g0592200|AY050	Barwin domain containing protein.	59	80	70	53	286	169	69.7142767	169.58008	2.432501463
Os03g0119600|AK108	Hypothetical protein.	62	55	40	105	181	96	52.43084	127.528783	2.432323864
POsControl0028|rando	NONE	15	15	12	16	75	11	14.0736833	34.2285533	2.432096312
Os09g0371100|CI3914	(No Hit)	19	10	13	24	64	12	13.8212657	33.5865333	2.430062061
Os11g0229500|AB019	Disease resistance protein family protein.	65	55	52	130	107	182	57.4604067	139.618767	2.429825592
Os09g0559000|Os09g	Zn-finger, cysteine-rich C6HC domain containing protein.	52	33	22	94	112	55	35.7869467	86.9074633	2.428468239
Os07g0688700|CI0516	Eggshell protein family protein.	17	7	6	11	50	11	9.85665867	23.9094667	2.425717221
POsControl0044|art|NA	NONE	16	8	5	6	57	10	10.0324457	24.32773	2.424905233
Os01g0498300|AK069	Protein of unknown function DUF563 family protein.	248	317	261	760	664	578	275.247	667.427767	2.42483212
Os02g0261800|Os02g	Cytochrome c heme-binding site containing protein.	54	51	28	35	131	154	44.0153433	106.702643	2.424214723
Os03g0180800|AK070	ZIM domain containing protein.	587	581	732	1405	1374	1826	633.402633	1534.93	2.423308523
Os04g0573000|AK066	SPX, N-terminal domain containing protein.	8	6	6	27	12	10	6.767364	16.398167	2.42312472
Os10g0471400|AK067	TPR-like domain containing protein.	11	12	10	29	23	28	11.0812703	26.8490767	2.422924074
Os06g0726200|AK105	Endochitinase precursor (EC 3.2.1.14).	114	120	100	215	383	210	111.3683	269.557967	2.420419156
Os03g0844600|AK059	Lipolytic enzyme, G-D-S-L family protein.	53	85	74	126	91	298	70.8671433	171.47771	2.419706819
Os04g0598500|AK111	Conserved hypothetical protein.	79	100	89	152	372	126	89.62808	216.832467	2.419247034
osa-miR159e|Os01|NA	miRNA	9	7	6	6	34	11	6.952675	16.8070613	2.417351787
Os03g0225900|AY055	Allene oxide synthase (EC 4.2.1.92).	130	176	176	401	353	409	160.732533	388.0869	2.41448879
Os01g0679300|CI3813	(No Hit)	10	11	6	7	45	11	8.78079	21.1966657	2.41398162
Os08g0386200|AY676	WRKY transcription factor 69.	1323	1428	1329	3509	2879	3456	1359.95433	3281.444	2.412907492
Os12g0116700|CI1829	WRKY transcription factor 64.	337	418	399	940	880	964	384.728733	928.1221	2.412406508
osa-miR395b|Os04|NA	miRNA	13	7	6	12	39	11	8.41564	20.2971133	2.411832414
Os11g0417800|CI4684	(No Hit)	66	45	22	36	117	165	43.9651867	105.99309	2.410841351

Os12g0103500|AK120(	Hypothetical protein.	37	46	46	72	56	185	43.1722267	104.04871	2.4100844
Os05g0576600|AK107	Conserved hypothetical protein.	779	748	797	1866	1801	1934	774.8999	1867.03733	2.40939163
osa-miR159e|Os01|NA	miRNA	9	7	6	7	25	25	7.762495	18.6996847	2.408978642
Os08g0198900|AK103(	DNA-binding WRKY domain containing protein.	202	245	246	587	441	643	231.251867	557.013	2.408685422
Os05g0574900|AK107:	GRAS transcription factor domain containing protein.	8	6	5	7	30	10	6.51260867	15.6826507	2.408044375
osa-miR397b|Os02|NA	miRNA	34	20	25	21	156	12	26.16128	62.9971533	2.408030239
Os10g0555400|Os10g(	Conserved hypothetical protein.	14	17	12	80	13	11	14.36293	34.5843967	2.407892865
Os10g0214300|AK064	Non-protein coding transcript, unclassifiable transcript.	16	15	8	11	73	10	13.0553553	31.42934	2.407390622
Os05g0162800|CI4198	(No Hit)	9	7	6	7	14	33	7.404136	17.8176843	2.40645017
Os11g0539600|Os11g(	Cyclin-like F-box domain containing protein.	21	24	16	18	118	11	20.34802	48.9658367	2.406417758
Os11g0147200|Os11g(	Peptidase, trypsin-like serine and cysteine proteases domain containin	23	17	21	42	42	62	20.31286	48.8739033	2.406057214
Os05g0211800|CI4380	(No Hit)	7	6	5	6	12	27	6.08305533	14.6335837	2.405630537
Os07g0625500|AK064(	Fimbriata-associated protein (Fragment).	39	36	23	65	125	45	32.6813333	78.5944967	2.40487424
Os01g0642000|AK110	Esterase/lipase/thioesterase domain containing protein.	74	59	36	61	322	23	56.2417933	135.250257	2.404799859
Os01g0657100|AF259	Phosphate transporter PT4.	32	21	9	26	107	15	20.61513	49.5570967	2.403918708
osa-miR159d|Os01|NA	miRNA	15	7	6	11	46	11	9.470184	22.76468	2.403826578
Os05g0148000|AK066:	Conserved hypothetical protein.	13	19	29	33	79	34	20.2689933	48.7040133	2.402882695
Os04g0451700|AK068	Conserved hypothetical protein.	33	33	24	56	85	75	29.96855	71.9903333	2.40219608
Os10g0150300|CI0956	Protein of unknown function DUF1210 family protein.	11	8	5	7	41	10	8.01795533	19.2554873	2.401545847
Os09g0441400|AK111(	Elicitor-inducible cytochrome P450.	6527	5982	6319	13808	15873	15534	6276.14233	15071.6933	2.401426311
Os04g0289800|CI4608	Zn-finger, RING domain containing protein.	9	7	6	7	34	12	7.268336	17.4347093	2.398720881
Os10g0400500|Os10g(	Tyrosine decarboxylase 1 (EC 4.1.1.25) (ELI5) (Fragment).	11	16	10	25	26	36	12.135801	29.0970467	2.397620616
Os10g0474300|Os10g(	Peptidase, trypsin-like serine and cysteine proteases domain containin	9	9	6	7	39	11	7.885305	18.893407	2.396027421
Os11g0686100|AK110	Conserved hypothetical protein.	149	155	146	171	281	624	149.739233	358.637467	2.395080158
Os04g0452100|AB073	Cryptochrome 1 apoprotein (Blue light photoreceptor).	211	223	229	518	501	566	220.6185	528.244667	2.394380647
Os03g0425000|AK109:	Conserved hypothetical protein.	8	6	5	23	12	10	6.257348	14.9753967	2.393249771
Os01g0139900|AK121(	Conserved hypothetical protein.	618	669	753	1768	1309	1804	679.879567	1627.08133	2.393190519
Os08g0111300|AK110	Transferase family protein.	125	116	131	261	305	324	123.976833	296.668833	2.392937659
POsControl0029|rando	NONE	9	8	6	6	36	11	7.385471	17.665629	2.391943452
Os07g0141400|AF022	23 kDa polypeptide of photosystem II.	94	74	50	161	235	124	72.4610033	173.166733	2.3897921
Os11g0669100|AK068(	Hypothetical protein.	54	74	91	198	121	205	73.0840367	174.638267	2.389554199
Os11g0117500|CI0619	(No Hit)	235	300	291	696	615	665	275.6259	658.610733	2.389509597
Os12g0242700|AK109	3-oxoacyl-[acyl-carrier-protein] reductase, chloroplast precursor (EC 1.	10	7	6	12	32	12	7.88481933	18.8408033	2.389503492
Os11g0152600|AK066	Non-protein coding transcript, uncharacterized transcript.	9	12	19	69	14	12	13.3403377	31.86822	2.388861571
Os09g0121600|Os09g(	Integrase, catalytic region domain containing protein.	34	24	14	22	78	72	23.9881167	57.2926033	2.388374383
Os02g0649900|CI4462	Iron-phytosiderophore transporter protein yellow stripe 1.	9	7	8	7	14	38	8.27473867	19.7589937	2.387869208
Os08g0460000|AF032	Germin-like protein 5 (Germin-like protein 1).	15	12	6	7	59	10	10.6754363	25.4853913	2.38729271
Os03g0405500|AK069	PDI-like protein.	4956	5643	4972	12733	10432	14008	5190.58667	12390.7733	2.387162402
Os01g0721800|AK120	Protein kinase-like domain containing protein.	17	10	5	23	46	10	10.9457327	26.111053	2.385500706
POsControl0018|genon	NONE	23	21	7	7	104	10	16.982336	40.501789	2.384936266
Os01g0621600|AK100	Protein of unknown function DUF1221 domain containing protein.	621	649	611	1613	1387	1484	626.7141	1494.64033	2.384883846

osa-miR439b|Os10|NA	miRNA	18	10	13	8	13	78	13.7595033	32.804407	2.384127261
osa-miR441c|Os01|NA	miRNA	18	13	6	9	69	12	12.4936707	29.7749073	2.383199312
POsControl0042|art|NA	NONE	8	6	5	6	29	10	6.31951467	15.052676	2.381935448
Os06g0130100|AK064(	ERECTA-like kinase 1.	35	25	9	18	132	12	22.6025243	53.8127133	2.380827581
Os03g0828800|AK107	Curculin-like (mannose-binding) lectin domain containing protein.	27	23	25	49	66	61	24.6184267	58.59938	2.380305646
Os06g0185800|AK120(	TPR-like domain containing protein.	38	21	20	30	143	14	26.2240367	62.4105167	2.379897399
Os10g0189600|AK100!	Aminotransferase, class I and II domain containing protein.	303	242	295	736	461	800	279.839033	665.804333	2.379240399
Os06g0168700|AK103(	Prolin rich protein.	37	33	19	33	158	19	29.3536933	69.8332433	2.379027489
Os11g0250100|AK119!	Hypothetical protein.	8	6	6	6	32	10	6.74863067	16.0546717	2.378952481
Os03g0719700|AK065	Conserved hypothetical protein.	66	76	64	149	161	181	68.80467	163.572467	2.377345414
Os01g0369200|AK073	Cullin family protein.	18	47	30	78	63	85	31.82888	75.6496867	2.376762446
Os01g0368900|AK107	GLUTAREDOXIN.	19	14	14	11	61	41	15.8473633	37.6640433	2.376675699
PGmControl0001|AF03	NONE	8	14	5	6	50	10	9.279484	22.0529863	2.376531533
Os12g0124000|AK060(	SMAD/FHA domain containing protein.	54	42	37	54	150	111	44.2320767	105.103747	2.376188382
Os12g0492900|AK106	Cyclin-like F-box domain containing protein.	8	6	6	6	32	10	6.776172	16.094599	2.375175689
Os04g0342500|Os04g(	Terpenoid synthase domain containing protein.	9	8	6	12	29	11	7.305365	17.3486533	2.374782551
Os08g0474000|AK062	AP2 domain containing protein RAP2.6 (Fragment).	116	89	80	200	225	252	95.1742367	225.948167	2.374047585
Os01g0734600|AK065	UDP-glucuronosyl/UDP-glucosyltransferase family protein.	8	21	5	6	64	10	11.1646837	26.50465	2.373972321
Os06g0673700|AK066!	Hypothetical protein.	774	975	971	2259	2023	2176	906.900333	2152.62967	2.373612168
Os03g0106800|AK069	Beta-expansin precursor.	10	6	5	6	16	27	6.84292567	16.2306223	2.371883478
Os01g0310500|AK099	Hypothetical protein.	120	100	119	101	624	79	113.023033	267.809383	2.369511554
osa-miR164c|Os05|NA	miRNA	91	73	53	61	416	37	72.42636	171.487287	2.367746863
Os01g0764900|AK066	Formamidase-like protein.	100	105	124	133	524	121	109.58812	259.443833	2.367444878
Os02g0268300|X1439	Glutelin.	40	31	15	22	121	59	28.4525367	67.3521833	2.367176752
Os04g0644600|AJ417	Epoxide hydrolase family protein.	50	38	28	34	103	137	38.5982467	91.3615633	2.366987395
Os07g0183200|AK064(	Conserved hypothetical protein.	14	8	7	7	48	12	9.40410633	22.2582913	2.366869381
Os11g0539100|CI4216	(No Hit)	9	7	6	7	20	25	7.32529467	17.3340473	2.366327653
Os06g0215900|AK105	Oxo-phytodienoic acid reductase.	269	281	292	702	643	647	280.7016	664.179133	2.366139464
Os04g0525200|AK121(	Hypothetical protein.	60	45	36	46	146	140	46.80003	110.676787	2.364887088
Os12g0291100|AK058	Ribulose bisphosphate carboxylase, small chain family protein.	51	23	11	35	48	119	28.40389	67.15762	2.364381076
Os10g0499800|AK099	Fertility restorer.	8	6	5	24	12	10	6.56892133	15.5176067	2.362276222
Os07g0663900|Os07g(	Short-chain dehydrogenase/reductase SDR family protein.	76	65	55	125	221	120	65.7459933	155.283733	2.361873712
Os07g0501700|AK070!	C2 domain containing protein.	8	6	5	8	15	22	6.26252367	14.789586	2.361601614
Os12g0609000|Os12g(	Resistance protein candidate (Fragment).	89	81	59	185	204	150	76.18829	179.799233	2.359932653
POsControl0037|art|NA	NONE	9	6	6	18	13	18	6.95034133	16.3928267	2.35856426
Os08g0196900|CI4360	(No Hit)	9	7	6	7	35	11	7.485403	17.6540097	2.358458144
Os12g0518200|AK070	Protein of unknown function DUF6 domain containing protein.	73	63	40	130	146	139	58.6257167	138.2548	2.358262003
Os09g0517100|AK119	Disease resistance protein family protein.	285	208	261	544	374	860	251.336133	592.629733	2.357916968
Os03g0856700|AK061(	Ga20 oxidase (Fragment).	1709	2185	2331	5479	4597	4599	2074.85733	4891.593	2.357556311
Os12g0457200|AK121	Non-protein coding transcript, uncharacterized transcript.	14	13	8	13	60	10	11.627437	27.3963813	2.356184027
Os01g0326300|AK058	Haem peroxidase, plant/fungal/bacterial family protein.	8	6	5	6	30	10	6.52137267	15.357676	2.354975982

Os10g0527800|AF4027	Tau class GST protein 3.	2589	3929	3358	8400	6108	8748	3292.13567	7752.02167	2.354709056
Os12g0112000|AK069	Peroxidase (EC 1.11.1.7).	3444	4332	2629	11676	6183	6641	3468.30067	8166.83833	2.354708867
Os07g0269000|CI2518	(No Hit)	331	465	414	1201	740	904	402.989833	948.180467	2.352864485
Os04g0543100|AK062	Non-protein coding transcript, unclassifiable transcript.	16	7	6	12	44	11	9.534653	22.43309	2.352795639
Os12g0498500|Os12g	Heat shock protein DnaJ, N-terminal domain containing protein.	8	6	5	6	28	10	6.23602133	14.667199	2.352012319
Os02g0198400|CI0285	Conserved hypothetical protein.	9	7	6	21	18	11	7.237412	17.0004233	2.348964427
PZmControl0001|X125	NONE	20	12	11	36	54	11	14.3945467	33.8115533	2.348914079
Os01g0793800|AK111	Conserved hypothetical protein.	105	219	168	417	319	419	163.944967	384.932633	2.347938099
Os04g0339800|CI2261	Protein kinase family protein.	49	47	41	121	104	97	45.6586367	107.20167	2.347894677
Os05g0530400|AK100	Heat shock factor protein 1 (HSF 1) (Heat shock transcription factor 1)	151	178	192	432	368	422	173.5458	407.132833	2.345967654
Os07g0236200|Os07g	Conserved hypothetical protein.	8	16	7	6	59	10	10.6501733	24.971938	2.344744749
Os03g0342400|CI4691	(No Hit)	50	34	48	101	110	97	43.7834733	102.580703	2.342909219
Os01g0948900|AK100	Ankyrin repeat containing protein.	121	91	78	249	206	222	96.4013033	225.834467	2.342649517
osa-miR167g|Os03|NA	miRNA	12	6	6	6	41	10	8.10578667	18.9867327	2.342367675
osa-miR395b|Os04|NA	miRNA	8	6	6	7	12	29	6.80342367	15.9337773	2.342023386
Os04g0668400|AK071	Conserved hypothetical protein.	9	7	7	6	35	11	7.435737	17.4140377	2.341938353
osa-miR395r|Os04|NA|	miRNA	8	7	6	6	31	11	6.89863933	16.154233	2.341654958
Os06g0647400|AK068	Lysosomal Pro-X carboxypeptidase.	192	241	114	440	397	443	182.2167	426.6043	2.341192108
Os09g0362500|AK068	Peptidase M1, membrane alanine aminopeptidase family protein.	925	892	971	2217	2000	2310	929.444067	2175.47433	2.340618883
Os08g0148500|CI5355	(No Hit)	8	6	5	7	28	10	6.394029	14.960776	2.339804214
osa-miR395j|Os08|NA|	miRNA	46	30	16	20	163	32	30.73801	71.90706	2.339353133
Os01g0110700|AK100	Phosphoenolpyruvate carboxylase (EC 4.1.1.31).	19	11	6	7	67	10	12.032634	28.1442043	2.338989479
Os10g0150700|AK104	Protein of unknown function DUF1210 family protein.	44	25	14	26	106	64	27.9184633	65.2633433	2.337640957
Os06g0507300|AK111	GAMYB-binding protein.	8	11	6	11	38	11	8.42208633	19.6851033	2.33731911
Os01g0753400|AK110	Conserved hypothetical protein.	26	27	15	35	104	20	22.6173067	52.8374733	2.336152315
Os03g0225900|AY310	Allene oxide synthase (EC 4.2.1.92).	65	78	78	191	134	190	73.56898	171.769233	2.334805149
Os08g0517100|AK112	LOB domain protein 1.	14	9	5	12	38	17	9.5712	22.33499	2.333562145
Os12g0420100|AK071	Beta-glucosidase.	13	6	5	6	42	10	8.315109	19.3865177	2.331480882
Os10g0532900|Os10g	Hypothetical protein.	10	16	19	21	75	11	15.2971967	35.6549	2.330812683
Os01g0151700|AK099	Short-chain dehydrogenase Tic32.	139	108	129	302	231	342	125.220433	291.852367	2.330708806
Os05g0217800|AK104	BURP domain containing protein.	8	7	6	19	18	11	6.935477	16.1631567	2.330503968
osa-miR399e|Os01|NA	miRNA	16	16	17	18	88	10	16.41638	38.2545563	2.330267473
Os08g0470000|CI4659	(No Hit)	46	34	26	24	83	140	35.4158533	82.5256733	2.330190171
Os01g0150400|Os01g	Protein tyrosine phosphatase-like protein, PTPLA family protein.	29	14	9	12	61	47	17.2341053	40.1410067	2.329161038
Os04g0644600|AK105	Epoxide hydrolase family protein.	34	28	16	30	133	18	25.9252833	60.38184	2.329071556
Os01g0521100|CI3343	Hypothetical protein.	9	7	6	7	35	12	7.694265	17.9185217	2.32881525
Os02g0705600|AK100	U box domain containing protein.	10	12	7	9	49	10	9.592041	22.3360623	2.328603718
Os06g0597900|AK065	SOUL heme-binding protein family protein.	42	37	24	24	110	107	34.5168933	80.37432	2.328550233
Os07g0132700|CI4046	(No Hit)	25	18	24	10	131	14	22.2618967	51.8139933	2.327474344
Os12g0547600|AK067	Hypothetical protein.	410	530	537	1275	864	1296	492.220267	1145.2026	2.326605948
Os02g0623300|AK105	Conserved hypothetical protein.	591	1012	495	2264	1035	1580	699.349333	1626.602	2.325879103

osa-miR171i|Os03|NA|(	miRNA	28	22	7	20	105	10	19.317079	44.9267803	2.325754341
Os04g0616700|AK111!	BRASSINOSTEROID INSENSITIVE 1-associated receptor kinase 1 pr	49	46	52	98	144	100	49.0643833	114.048933	2.324475018
Os01g0863800|Os01g(	Uncharacterized plant-specific domain containing protein.	178	139	84	461	258	214	133.871853	311.163867	2.324341218
Os11g0620300|AK107!	Nonspecific lipid-transfer protein 2 (LTP 2).	27	19	12	14	97	24	19.39088	45.0517533	2.323347539
Os06g0306600|AK099	Esterase/lipase/thioesterase domain containing protein.	12	25	13	30	71	16	16.78271	38.9708867	2.322085448
Os06g0711100|AK100	Hypothetical protein.	30	30	60	52	186	42	40.0417367	92.97089	2.321849593
Os10g0105900|CI4532	(No Hit)	7	9	9	7	18	34	8.49234767	19.7170627	2.321744639
Os03g0129100|AF384(	Seven transmembrane protein MLO2.	1646	2162	2322	5478	3766	4989	2043.559	4744.34933	2.321611137
Os09g0131600|Os09g(	Conserved hypothetical protein.	29	13	8	12	72	34	16.95757	39.3634233	2.321289155
Os04g0434900|Os04g(	Conserved hypothetical protein.	40	21	14	20	144	11	25.0863033	58.2234233	2.320924791
osa-miR172c|Os07|NA	miRNA	8	6	5	6	12	27	6.460832	14.98537	2.319417994
Os01g0966200|AK098!	Protein of unknown function YGGT family protein.	101	160	134	358	259	297	131.516067	304.912267	2.318441194
POsControl0040|art|NA	NONE	14	8	6	6	21	38	9.30333767	21.5631043	2.317781543
Os08g0106400|Os08g(	Conserved hypothetical protein.	51	55	45	42	74	236	50.6592933	117.34381	2.316333337
Os07g0561800|AK111	Hypothetical protein.	50	42	57	112	109	126	50.03293	115.8783	2.316040656
Os01g0210400|CI1485	Protein of unknown function DUF616 family protein.	47	35	25	34	197	17	35.7323567	82.7445867	2.315676725
Os12g0547600|AK067	Hypothetical protein.	541	734	794	1829	1224	1736	689.646467	1596.202	2.314522117
Os11g0707000|AK064!	Ribulose-bisphosphate carboxylase activase (EC 6.3.4.-) (Fragments).	9	7	17	20	45	12	11.05132	25.56298	2.313115537
Os01g0609300|AK100	PDR-like ABC transporter (PDR3 ABC transporter).	80	134	116	265	230	268	110.06313	254.4683	2.312021292
Os04g0596900|CI2497	(No Hit)	80	59	29	120	176	93	55.91559	129.199597	2.3106185
Os11g0290600|AK070	Proteinase inhibitor I4, serpin family protein.	71	80	29	190	137	89	60.02165	138.593373	2.309056371
Os02g0452000|AK063	Hypothetical protein.	18	12	10	8	75	10	13.4103767	30.942649	2.307366137
Os07g0151900|CI4035	(No Hit)	7	8	5	10	27	10	6.75554333	15.581422	2.306464666
Os11g0302600|CI5262	(No Hit)	22	17	5	16	78	10	14.9840677	34.5136467	2.3033563
Os03g0742300|AK108	Response regulator receiver domain containing protein.	19	12	6	7	66	12	12.3518683	28.4459853	2.302970252
Os04g0547900|AK065!	MutT domain protein-like.	1288	1540	1494	3649	2529	3774	1440.74133	3317.69833	2.302771675
Os11g0227700|CI2505	RPR1.	564	610	644	1528	1028	1631	606.136467	1395.59633	2.302445753
Os02g0148000|AF448	Zinc finger protein.	11	9	6	12	39	11	8.887089	20.4540433	2.301545909
Os05g0158500|AK106	Peptidase S10, serine carboxypeptidase family protein.	1333	1380	1732	3725	2796	3704	1481.384	3408.19767	2.30068481
Os02g0615800|AK065(	Protein kinase domain containing protein.	28	26	12	31	59	61	21.84213	50.22005	2.299228601
Os03g0195100|AY338	Aminotransferase, class I and II domain containing protein.	91	81	61	144	221	169	77.5370267	178.168967	2.297856577
Os02g0554100|AK069!	UVB-resistance protein UVR8.	13	12	9	16	34	31	11.6743167	26.8228133	2.297591722
Os01g0142000|CB650	Hypothetical protein.	18	11	13	6	18	74	14.07366	32.3351873	2.297567749
Os10g0546300|AY224!	Conserved hypothetical protein.	23	22	19	22	107	19	21.4970333	49.3769433	2.296918955
Os02g0736700|CB632	Hypothetical protein.	24	7	6	12	62	11	12.3683963	28.4068933	2.296732136
Os07g0622300|Os07g(	UDP-glucuronosyl/UDP-glucosyltransferase family protein.	8	10	9	9	45	11	9.41996367	21.6299117	2.296177823
Os06g0602500|AK111	Protein kinase domain containing protein.	58	70	62	146	144	147	63.38947	145.5494	2.296113219
Os01g0382700|AK060(	Conserved hypothetical protein.	7	6	5	6	26	9	5.97587233	13.7202113	2.295934479
Os04g0340400|AK068	Hypothetical protein.	8	6	5	6	29	10	6.54544333	15.024692	2.295442988
Os09g0323900|Os09g(	Haem peroxidase, plant/fungal/bacterial family protein.	56	48	80	223	62	138	61.5189667	141.199037	2.29521145
osa-miR172a|Os09|NA	miRNA	8	6	6	24	13	10	6.81636167	15.6431333	2.294938869

Os12g0440400|AK064(	Hypothetical protein.	35	25	16	16	111	45	25.0488533	57.46802	2.294237554
Os04g0528000|Os04g(	Protein of unknown function DUF789 family protein.	17	9	5	17	45	10	10.418183	23.8803983	2.292184571
Os11g0280500|AK073	Hypothetical protein.	75	53	53	280	103	30	60.0811267	137.696757	2.291847112
Os01g0847700|AK103	Aldose reductase.	1816	1623	1874	4627	3089	4461	1771.134	4058.80933	2.291644412
osa-miR445d|Os05|NA	miRNA	8	16	7	14	38	21	10.5455387	24.16538	2.291526376
Os02g0206700|CI5075	UDP-glucuronosyl/UDP-glucosyltransferase family protein.	23	7	16	31	62	14	15.6815343	35.9219467	2.290716323
Os01g0704000|AK069	Plant invertase/pectin methylesterase inhibitor domain containing prote	9	8	6	12	31	11	7.937726	18.1817033	2.290543077
Os01g0730400|AK102	Hypothetical protein.	20	43	39	49	169	15	33.9529067	77.7617633	2.290282953
Os03g0154000|AK103(	Aromatic-ring hydroxylase family protein.	4016	3931	4738	9197	10830	9025	4228.49733	9684.05	2.290187089
Os06g0215900|AK072	Oxo-phytodienoic acid reductase.	259	281	282	685	617	581	274.153	627.717533	2.289661369
Os04g0234600|AK062(	Sedoheptulose-1,7-bisphosphatase, chloroplast precursor (EC 3.1.3.37	52	11	13	11	152	10	25.16841	57.5738583	2.287544518
Os05g0591600|CI3941	Lysine decarboxylase-like protein.	23	18	25	47	63	42	22.1023433	50.54623	2.286917239
Os04g0275100|AK106(	EGF-like calcium-binding domain containing protein.	52	52	31	109	113	85	44.89268	102.651883	2.286606265
Os10g0433100|CI1505	(No Hit)	272	353	247	628	649	716	290.602967	664.165733	2.285474718
Os07g0556800|AK107	Ribosome-inactivating protein family protein.	8	7	13	6	48	10	9.36221533	21.391438	2.284869258
Os09g0331100|Os09g(	Ankyrin repeat containing protein.	10	9	5	27	18	10	8.00084333	18.2683463	2.283302594
POsControl0028|rando	NONE	18	14	6	14	62	10	12.63656	28.8490933	2.282986298
Os07g0416100|AK063(	Conserved hypothetical protein.	35	37	26	78	113	34	32.94509	75.1779433	2.281916466
Os03g0117100|AK070	Peroxisomal biogenesis factor 11 family protein.	103	57	63	141	161	207	74.40795	169.790533	2.281886994
Os07g0600000|AK105	Hypothetical protein.	9	7	6	6	31	11	7.02435067	16.024778	2.281318055
Os11g0211400|AK059	Ankyrin repeat containing protein.	9	7	6	6	27	16	7.152341	16.316428	2.281270985
Os01g0533900|AK106(	Multidrug resistance protein 1 homolog.	428	613	560	1375	974	1302	533.596333	1216.93743	2.28063305
Os08g0474800|Os08g(	Esterase/lipase/thioesterase domain containing protein.	1237	1208	1251	3111	2675	2643	1232.12267	2809.63067	2.280317328
Os03g0225900|AK066	Allene oxide synthase (EC 4.2.1.92).	108	139	133	304	264	297	126.5036	288.367333	2.279518791
Os02g0618400|AK099	MYB8 protein.	259	230	183	556	547	429	224.0463	510.499733	2.278545699
Os01g0795600|AK111(	Conserved hypothetical protein.	95	99	100	235	194	240	97.9097967	223.041567	2.27803115
Os07g0277500|Os07g(	Hypothetical protein.	8	6	6	6	29	10	6.57573433	14.9781063	2.27778459
Os02g0317700|AK111	Conserved hypothetical protein.	30	33	21	41	130	22	28.32205	64.5036267	2.277505571
Os01g0613500|AK070	Peptidase C1A, papain family protein.	51	52	25	37	119	137	42.8460933	97.5814267	2.277487142
Os02g0492900|AK110	Ankyrin-like protein-like protein.	17	12	7	9	64	10	12.0477707	27.4365913	2.277316866
Os04g0630300|AK059	Conserved hypothetical protein.	7	7	5	6	20	17	6.269532	14.266206	2.275481806
Os03g0108400|Os03g(	Snf7 family protein.	43	36	23	26	186	18	33.6730033	76.5930733	2.274613659
Os03g0328200|Os03g(	Conserved hypothetical protein.	12	6	7	6	41	10	8.35266233	18.998557	2.274551064
Os03g0190100|AK066	UbiA prenyltransferase family protein.	59	102	115	249	170	208	91.9049067	208.9758	2.273826367
Os04g0627000|AK101	Roc1.	15	23	14	14	20	82	17.08028	38.83423	2.273629589
Os04g0491100|AK069(	Conserved hypothetical protein.	31	45	41	124	51	91	39.0972867	88.88093	2.273327322
Os10g0558200|Os10g(	2OG-Fe(II) oxygenase domain containing protein.	61	61	55	35	313	54	59.0841333	134.258307	2.272324211
Os03g0129400|AK099	Hypothetical protein.	35	45	29	35	62	151	36.3176133	82.5038633	2.271731421
PGmControl0003|AF03	NONE	17	13	5	10	61	10	11.8079643	26.8049707	2.270075511
Os10g0111700|AK069	Nitrate transporter (Fragment).	17	13	9	15	62	12	12.946649	29.38664	2.26982596
Os03g0181500|AK060	Beta-ketoacyl-CoA synthase.	100	144	140	211	483	178	128.0448	290.638867	2.269821708

Os05g0571200|AK108:	WRKY transcription factor 19.	786	175	188	644	851	1111	383.052333	868.957967	2.268509786
Os10g0117600|CI2847	(No Hit)	76	77	81	177	181	175	78.2283933	177.434533	2.268160265
Os03g0781400|AK071!	Ribose 5-phosphate isomerase family protein.	14	9	15	44	28	15	12.700238	28.79641	2.267391367
Os07g0131100|Os07g(	Protein kinase family protein.	10	8	7	10	34	13	8.312663	18.8439133	2.266892491
Os08g0351200|CI4485	Plant protein of unknown function family protein.	20	11	12	9	44	44	14.18573	32.1529863	2.266572558
Os01g0517800|CI4905	Protein of unknown function DUF597 family protein.	19	7	6	19	44	11	10.9171677	24.73547	2.265740598
Os06g0587100|Os06g(	Conserved hypothetical protein.	76	85	62	157	195	153	74.4294033	168.593867	2.265151393
Os12g0472800|AK063:	Hypothetical protein.	3808	4694	3454	10352	8218	8510	3985.15167	9026.81267	2.265111449
Os06g0493100|AK063	Hypothetical protein.	1204	2463	1562	3636	4119	4089	1743.059	3948.09933	2.26504056
Os01g0303000|AK066:	CP12 protein-like protein.	8	13	5	15	34	10	8.639021	19.56031	2.264181323
Os10g0361000|AK058!	Lipase/lipooxygenase, PLAT/LH2 domain containing protein.	214	304	205	861	383	392	240.8418	545.2545	2.263952935
Os11g0166300|Os11g(	Hypothetical protein.	18	10	6	10	56	10	11.2458003	25.4569133	2.263681782
Os06g0632800|AK060	Hypothetical protein.	20	16	17	16	93	10	17.4431633	39.4789237	2.263289228
Os02g0618400|AK111!	MYB8 protein.	490	505	403	1190	993	981	466.0261	1054.6771	2.263128825
Os09g0362600|Os09g(	Peptidase M1, membrane alanine aminopeptidase family protein.	42	35	22	70	106	49	33.12863	74.8870633	2.260493819
Os03g0201500|CI5623	(No Hit)	74	118	118	267	178	254	103.10008	232.979567	2.259741861
Os09g0346500|D0064	Chlorophyll a-b binding protein 1, chloroplast precursor (LHCII type I C.	46	25	14	21	135	34	28.1543067	63.6167933	2.259575918
Os08g0540900|AK111(	Conserved hypothetical protein.	86	86	95	203	181	220	89.1987767	201.481933	2.258797047
Os01g0147800|AK106!	Protein of unknown function DUF547 domain containing protein.	49	37	19	32	101	105	35.0602833	79.16351	2.257925563
Os02g0751600|AK104	Peptidylprolyl isomerase, FKBP-type domain containing protein.	8	6	5	21	12	10	6.290394	14.201287	2.257614865
Os11g0241700|AK068:	Protein of unknown function DUF538 family protein.	687	2057	1199	3817	2257	2828	1314.39233	2967.34833	2.257581894
Os09g0432300|CI4984	AAA ATPase, central region domain containing protein.	91	77	61	116	263	138	76.40845	172.4678	2.257182288
Os11g0592200|AK121	Barwin domain containing protein.	64	78	58	56	193	201	66.6207367	150.357897	2.256923357
Os07g0485500|AK121(	Protein of unknown function DUF6 domain containing protein.	10	15	12	23	36	24	12.197565	27.5288	2.256909473
Os02g0787700|AK070	Conserved hypothetical protein.	14	9	6	29	14	22	9.58446167	21.62331	2.256079762
Os07g0214100|D1143	Seed allergenic protein RA17 precursor.	7	20	6	6	60	9	11.0601533	24.947233	2.255595582
Os02g0202700|Os02g(	Serine acetyltransferase.	66	78	64	74	97	299	69.4807567	156.711723	2.25546944
Os11g0649900|CI2345	Exo70 exocyst complex subunit family protein.	106	119	92	199	237	278	105.620607	238.208967	2.255326628
Os01g0731800|AK121	Zn-finger, RING domain containing protein.	88	85	75	211	200	149	82.83246	186.757367	2.254639868
Os01g0382700|AK071(	Conserved hypothetical protein.	8	6	10	20	24	12	8.28881133	18.6833633	2.254046157
Os04g0690500|AK105!	Conserved hypothetical protein.	9	7	6	7	31	12	7.31740067	16.491914	2.253794039
Os05g0232200|AK071(	Conserved hypothetical protein.	16	14	5	8	61	10	11.7116777	26.3944677	2.253688021
osa-miR395f|Os04|NA|	miRNA	10	18	7	17	47	13	11.523471	25.9541967	2.252289841
PGmControl0003|AF03	NONE	26	18	7	12	91	12	16.8921793	38.0401033	2.251935797
Os08g0151200|CI2656	(No Hit)	36	44	48	99	91	99	42.74136	96.2297933	2.251444346
Os01g0877400|AK106	Avr9 elicitor response-like protein.	9	6	6	8	29	10	6.86076167	15.4420667	2.250780222
Os03g0820400|AY305!	ZPT2-13.	208	352	323	774	513	702	294.712633	662.9782	2.249575095
Os11g0264300|AK068	Plant regulator RWP-RK domain containing protein.	9	7	12	15	14	35	9.359249	21.0506767	2.249184381
Os08g0332700|AK099!	Trans-acting transcriptional protein ICP0 (Immediate-early protein IE11	9	13	6	6	45	11	9.25033833	20.7996713	2.248530874
Os04g0149400|AK062	Hypothetical protein.	387	552	480	975	1044	1172	473.1613	1063.7393	2.248153642
PAtControl0003|AY056	NONE	9	7	7	7	33	12	7.695645	17.3003033	2.248064111

Os02g0188000|AK105	UDP-glucuronosyl/UDP-glucosyltransferase family protein.	1776	2062	2238	4434	4629	4591	2025.47767	4551.31833	2.247034568
Os04g0295400|AK067	Jacalin-related lectin domain containing protein.	26	24	9	13	74	44	19.462487	43.7191833	2.246330766
Os09g0549900|CB646	Hypothetical protein.	27	34	37	39	106	76	32.8652333	73.8256433	2.246314292
Os11g0474000|AK067	Hypothetical protein.	12	13	7	6	56	10	10.6934767	24.0197933	2.246209917
Os12g0558400|AK073	Hypothetical protein.	9	7	6	7	31	12	7.21468967	16.203126	2.245852108
Os12g0140700|AK108	Zn-finger, RING domain containing protein.	40	6	13	6	116	10	19.4842407	43.7454067	2.245168668
Os03g0800200|CI1069	Argonaute protein.	107	71	88	251	139	207	88.6575967	199.0304	2.244933401
Os06g0349700|CI0172	(No Hit)	137	508	266	844	520	680	303.5878	681.2328	2.243939974
Os01g0216000|AK104	Lipolytic enzyme, G-D-S-L family protein.	4030	11842	5459	19507	12870	15485	7110.23767	15953.7833	2.24377638
POsControl0037|art|NA	NONE	8	7	6	6	29	11	6.945187	15.582589	2.243652907
Os09g0522000|AK062	CBF-like protein.	541	765	619	1559	1352	1407	641.689867	1439.41767	2.243167208
Os11g0582500|D2973	Anther specific protein.	12	6	5	15	27	10	7.65549967	17.17072	2.242926099
Os09g0436200|AK109	Conserved hypothetical protein.	25	36	25	15	169	9	28.8778833	64.7534853	2.242321038
Os01g0837700|Os01g	Conserved hypothetical protein.	47	34	23	79	115	39	34.55593	77.48272	2.242240912
Os09g0476700|AK063	Non-protein coding transcript, unclassifiable transcript.	22	15	10	12	86	10	15.87924	35.601394	2.242008686
Os06g0569900|AY660	Ent-kaurene oxidase (EC 1.14.13.78) (AtKO1) (Cytochrome P450 701A	31	20	21	46	82	31	23.5684267	52.83871	2.24192776
Os01g0821300|CI1772	(No Hit)	44	21	19	56	98	36	28.16623	63.14553	2.241887892
POsControl0044|art|NA	NONE	29	11	11	6	96	12	16.7858767	37.6207143	2.241212364
Os03g0829200|AK108	Soluble epoxide hydrolase.	190	213	177	448	429	422	193.293033	433.101867	2.240649128
Os01g0821600|AK108	DNA-binding WRKY domain containing protein.	67	67	51	103	173	138	61.55947	137.927333	2.240554269
Os04g0174100|CI1231	E-class P450, group I family protein.	25	12	10	46	50	10	15.8076267	35.4123633	2.240207469
Os02g0601800|AK121	Bromodomain containing protein.	9	7	9	6	38	11	8.17582833	18.3139377	2.240010054
Os07g0296000|AK073	Conserved hypothetical protein.	20	33	27	82	61	36	26.5385067	59.4391733	2.239733158
Os03g0702100|AK063	Conserved hypothetical protein.	27	18	26	59	62	40	23.8676967	53.45363	2.239580582
Os02g0821000|CI4067	(No Hit)	9	7	6	7	33	12	7.74877833	17.351846	2.239300862
Os06g0536800|CB641	Peptidase aspartic family protein.	21	26	17	33	102	10	21.5176433	48.161878	2.238250595
osa-miR169l|Os08|NA|	miRNA	12	17	7	19	53	10	12.219519	27.3195573	2.235730992
Os03g0702000|AK105	UDP-glucuronosyl/UDP-glucosyltransferase family protein.	283	327	337	671	701	746	315.7984	705.888333	2.235249872
Os09g0522800|AK070	Hypothetical protein.	283	306	314	716	577	724	300.850267	672.229833	2.234433231
Os07g0653000|Os07g	BLE2 protein.	295	319	404	732	606	935	339.2419	757.793733	2.233785783
Os03g0197200|AK073	Sorbitol transporter.	9	7	6	8	29	13	7.53863667	16.8393593	2.233740672
Os01g0968800|AY345	Avr9/Cf-9 rapidly elicited protein 111B.	8	7	6	15	20	12	6.99249133	15.61835	2.233588753
Os02g0177400|AK102	Conserved hypothetical protein.	16	19	22	36	39	51	18.8518467	42.0918733	2.232771891
Os08g0406400|AK062	Sulfate transporter (Fragment).	113	95	78	183	277	179	95.4947233	213.1844	2.23242073
Os08g0248100|AK111	Protein kinase domain containing protein.	104	168	173	378	241	373	148.104233	330.5517	2.231885562
Os07g0656900|AY224	Serine carboxypeptidase II chains A and B (EC 3.4.16.6) (Carboxypept	2409	2591	2198	6286	4341	5420	2399.277	5348.81133	2.229342978
Os02g0653300|AK071	Conserved hypothetical protein.	66	68	71	110	85	264	68.6240133	152.95731	2.228918167
Os05g0171900|AK106	Glyoxalase/bleomycin resistance protein/dioxygenase domain containi	45	53	34	48	219	27	44.10534	98.30704	2.228914685
Os01g0213500|CI4261	Conserved hypothetical protein.	123	123	108	272	268	250	118.1074	263.249267	2.228897314
Os05g0137000|AK107	Hypothetical protein.	17	24	22	63	36	42	21.0290133	46.8640467	2.228542344
POsControl0012|genon	NONE	15	7	10	7	53	11	10.670404	23.773875	2.228020139

Os03g0183500|AK063(	Protein of unknown function DUF581 family protein.	252	206	170	462	430	506	209.188367	466.032367	2.227812063
Os07g0645500|AU222	Endonuclease/exonuclease/phosphatase family protein.	19	10	5	6	61	10	11.380956	25.3458547	2.227040915
Os03g0643200|Os03g(	EGF-like calcium-binding domain containing protein.	153	173	145	271	380	397	156.854833	349.192433	2.226214047
Os07g0142100|CI0548	Conserved hypothetical protein.	10	9	8	7	42	12	9.15771	20.383902	2.225873281
Os05g0448300|CI3324	Phospholipid/glycerol acyltransferase family protein.	12	7	6	31	15	12	8.61444933	19.1729567	2.225674088
Os08g0448000|AK120	4-coumarate--CoA ligase 1 (EC 6.2.1.12) (4CL 1) (4-coumaroyl-CoA sy	219	235	243	509	427	616	232.672767	517.740267	2.22518636
Os01g0836200|AK068	Conserved hypothetical protein.	72	48	37	114	192	43	52.40621	116.54735	2.223922508
Os02g0450400|Os02g(	Integrase, catalytic region domain containing protein.	43	31	24	94	67	56	32.46585	72.18297	2.22335069
POsControl0014|genon	NONE	11	10	10	19	41	10	10.5101967	23.3388567	2.220591813
Os01g0180900|CI4893	(No Hit)	11	6	8	17	27	10	8.127683	18.0462637	2.220345413
Os01g0102600|AK064	Shikimate kinase domain containing protein.	249	228	265	597	463	589	247.636967	549.7261	2.219887069
Os04g0182600|Os04g(	Conserved hypothetical protein.	13	6	5	6	26	20	7.706897	17.1027047	2.219142758
Os08g0182900|CI2465	(No Hit)	11	8	7	12	33	12	8.393518	18.6186533	2.218218074
osa-miR167f|Os10|NA|	miRNA	11	6	6	8	32	11	7.684817	17.0430773	2.217759686
Os06g0721800|CI5625	(No Hit)	21	15	17	24	51	41	17.3587067	38.4826567	2.216908057
Os02g0488600|Os02g(	U box domain containing protein.	31	26	7	23	58	60	21.1600107	46.9080467	2.216825285
Os12g0274200|AK063(	Lipolytic enzyme, G-D-S-L family protein.	8	6	6	6	28	10	6.619385	14.6709547	2.216362195
Os03g0856300|AK108	Acyl carrier protein, chloroplast precursor (ACP) (ACP09) (Clone 22C0	6376	3641	5118	10182	9925	13429	5044.82	11178.4107	2.215819527
Os01g0319400|AK070	Plant protein of unknown function family protein.	8	6	5	6	27	10	6.426333	14.2346833	2.215055356
Os01g0108600|AK109	Basic helix-loop-helix dimerisation region bHLH domain containing prot	99	81	89	198	163	236	89.7250767	198.7119	2.214675176
Os02g0177300|CI1473	Squamosa-promoter binding protein 1.	19	7	6	6	53	11	10.5772817	23.4206107	2.214237212
Os01g0116400|AK066(	Protein kinase domain containing protein.	14	14	16	15	51	32	14.7405633	32.6373567	2.214118682
Os11g0117900|CI4253	(No Hit)	217	343	336	791	545	647	298.505233	660.894	2.214011435
Os06g0289200|AK106	UDP-glucuronosyl/UDP-glucosyltransferase family protein.	126	142	115	268	350	229	127.474233	282.2004	2.213783857
Os01g0796000|CI5089	(No Hit)	35	29	41	44	150	40	35.16746	77.8516633	2.213741434
Os06g0239100|CI0425	Peptidase M10A and M12B, matrixin and adamalysin family protein.	3175	3167	3184	6930	5751	8405	3175.242	7028.62333	2.213570913
Os08g0408300|AK100	Conserved hypothetical protein.	25	21	13	16	104	11	19.7231167	43.6522133	2.213251286
Os04g0626400|AK059	Calycin family protein.	104	98	106	168	354	162	102.940793	227.807333	2.21299376
Os02g0133400|Os02g(	Structural maintenance of chromosome protein SMC, C-terminal doma	19	11	19	26	70	11	16.1781467	35.7874	2.212082801
Os07g0664200|AK109(	Glucose/ribitol dehydrogenase family protein.	58	66	52	137	126	124	58.3928433	129.144567	2.211650594
Os02g0211000|Os02g(	Viral coat and capsid protein family protein.	56	39	16	43	165	36	36.90453	81.60244	2.211176785
Os04g0547900|AK060	MutT domain protein-like.	536	654	615	1444	1040	1506	601.7162	1329.95767	2.210273991
Os08g0550700|Os08g(	Auxin induced protein.	87	90	82	86	417	70	86.5341633	191.120027	2.208607783
Os11g0255300|AK071	Peptidase C1A, papain family protein.	12	9	7	6	44	11	9.21832633	20.3591617	2.208552934
Os07g0116900|AK065	Conserved hypothetical protein.	50	72	23	83	159	78	48.3835733	106.810547	2.207578715
POsControl0045|art|NA	NONE	12	13	7	10	48	13	10.6793573	23.569646	2.2070285
Os02g0658500|AK107	Hypothetical protein.	10	8	7	13	29	13	8.359135	18.4456667	2.206647777
Os03g0225900|AK061	Allene oxide synthase (EC 4.2.1.92).	123	153	160	331	297	331	145.0722	319.876133	2.204944389
osa-miR397b|Os02|NA	miRNA	70	51	32	46	137	157	51.4183367	113.26404	2.20279471
Os07g0162900|AK106	Esterase/lipase/thioesterase domain containing protein.	94	112	102	226	210	243	102.742417	226.209333	2.201713184
Os10g0439800|Os10g(	Cytochrome P450 family protein.	76	95	102	140	230	230	90.78422	199.844233	2.201310242

Os12g0276300|AK109(	Hypothetical protein.	9	7	7	7	32	11	7.55444167	16.629557	2.201295309
Os08g0201100|CI5500	(No Hit)	152	178	201	405	316	447	176.8483	389.218733	2.20086217
Os07g0489200|CI4857	(No Hit)	342	336	313	654	978	550	330.552033	727.337333	2.200371681
Os11g0474800|AB118(	Terpene synthase-like domain containing protein.	133	163	157	370	311	315	151.0326	332.253533	2.199879584
Os02g0731700|AK072:	CONSTANS-like 1 protein.	7	16	13	17	48	14	11.8924473	26.1415633	2.198165155
Os03g0648500|AK107	Plastocyanin-like domain containing protein.	12	6	6	21	13	18	7.93079567	17.42896	2.197630696
Os04g0278900|AK108	Dihydrouridine synthase, DuS family protein.	8	7	6	22	13	11	6.91091767	15.18749	2.197608296
osa-miR167i|Os06|NA|	miRNA	9	7	6	7	14	29	7.46126767	16.3922213	2.196975375
Os04g0107900|AK105:	Heat shock protein 81-1 (HSP81-1) (Heat shock protein 83).	10	10	6	7	41	11	8.957045	19.6757373	2.19667729
Os06g0655200|AK121(	Pollen allergen Lol p2 family protein.	8	6	5	6	12	24	6.23673467	13.6993653	2.196560551
Os09g0423800|AK111(	AP2-1 protein (Fragment).	1415	2010	1982	4788	2912	4175	1802.187	3958.078	2.196263762
Os01g0117600|AK065	Protein kinase domain containing protein.	43	44	53	108	100	99	46.6123133	102.32859	2.195312412
osa-miR419|Os12|NA|c	miRNA	8	6	5	8	23	10	6.20431733	13.619559	2.195174468
Os12g0109100|CI1220	(No Hit)	10	6	5	6	31	10	7.112816	15.611536	2.194846036
Os08g0544300|AK119:	Hypothetical protein.	33	23	19	24	113	27	25.0463533	54.94232	2.193625526
Os06g0625300|Os06g(	Peptidoglycan-binding LysM domain containing protein.	75	64	54	140	142	140	64.1395167	140.676067	2.1932823
Os07g0106700|AK060(	Hypothetical protein.	1654	4632	1309	8432	3516	4705	2531.746	5551.01833	2.192565263
Os02g0702700|CI5429	(No Hit)	14	14	15	20	63	12	14.5042233	31.7677233	2.190239533
Os01g0227800|CI4078	Cytochrome P450 family protein.	38	37	23	51	126	39	32.8467167	71.9373367	2.19009216
Os08g0402100|CI1020	(No Hit)	10	7	6	7	33	12	7.79848533	17.074922	2.189517742
Os04g0547600|AK109	Pathogenesis-related transcriptional factor and ERF domain containing	13	10	12	12	55	11	11.87508	25.99984	2.189445461
Os07g0204900|AK062(	Zeta-carotene desaturase (Fragment).	2811	3079	3360	7594	5387	7263	3083.08933	6748.15533	2.188764127
osa-miR159a|Os01|NA	miRNA	8	6	5	6	26	10	6.40716	14.0231387	2.188666846
Os01g0731600|Os01g(	Conserved hypothetical protein.	125	96	111	230	268	228	110.519213	241.882733	2.188603466
Os02g0585200|AU174	Heavy metal transport/detoxification protein domain containing protein.	529	528	590	1197	1051	1353	548.604867	1200.44733	2.188182071
Os03g0758900|AY341	WRKY transcription factor 34.	15	14	12	25	45	20	13.7734033	30.1336033	2.187811001
Os06g0476200|AK068	Phosphoglucomutase precursor (EC 5.4.2.2).	12	7	7	7	39	12	8.76100367	19.1668967	2.187751244
Os09g0346500|AK104:	Chlorophyll a-b binding protein 1, chloroplast precursor (LHCII type I C.	231	146	95	257	430	347	157.577387	344.557567	2.186592721
Os06g0264800|AK099	Conserved hypothetical protein.	32	16	15	20	107	11	21.03215	45.9749333	2.185935976
POsControl0006|genon	NONE	60	36	24	35	127	102	40.1746267	87.8154867	2.185844498
Os06g0598500|AU076:	Ribulose bisphosphate carboxylase large chain precursor (EC 4.1.1.39	2930	2695	3053	6112	4730	8128	2892.754	6323.05267	2.185824535
Os11g0518600|AK106	Hypothetical protein.	11	18	7	16	52	11	12.0406737	26.3157867	2.185574279
Os03g0842900|AK107	Short-chain dehydrogenase/reductase SDR family protein.	10	8	7	11	32	12	8.52519	18.63008	2.185297923
Os04g0517700|Os04g(	EGF-like calcium-binding domain containing protein.	42	19	16	20	82	65	25.5326867	55.7927533	2.18515012
Os01g0917100|AK107:	Conserved hypothetical protein.	40	21	14	18	89	56	24.9576267	54.5352867	2.185115091
Os04g0351300|Os04g(	Protein prenyltransferase domain containing protein.	9	7	6	8	27	11	7.13584033	15.5884103	2.184523421
Os11g0289700|AK060:	E-class P450, group I family protein.	8	42	35	135	22	27	28.1496977	61.4935533	2.18451914
Os01g0805200|AK104	Conserved hypothetical protein.	148	159	166	397	267	368	157.5816	343.951767	2.182689899
Os11g0668600|Os11g(	Hypothetical protein.	8	30	14	57	30	26	17.3997977	37.9774333	2.182636492
Os05g0436000|CI3298	(No Hit)	84	68	53	53	172	224	68.4000133	149.268757	2.182291339
Os01g0944700|AK070(	Glucan endo-1,3-beta-glucosidase GII precursor (EC 3.2.1.39) ((1->3)-	22	8	6	15	53	11	12.0458623	26.2844233	2.182029199

osa-miR167f|Os10|NA|	miRNA	30	9	10	7	49	50	16.3308283	35.629272	2.181718604
Os08g0539400|AY336	Resistance protein T10rga2-1A.	10	8	9	8	14	35	8.820905	19.2443177	2.181671571
osa-miR395g|Os04|NA	miRNA	27	19	15	14	101	17	20.1964033	44.0575933	2.181457392
Os10g0484800|X9181	Anther-specific protein YY2.	9	14	6	9	41	11	9.340274	20.3708987	2.18097442
Os01g0721000|AK105	NB-ARC domain containing protein.	3940	5403	5449	11620	9813	10822	4930.65933	10751.4057	2.180520888
Os03g0403400|AK111	TolB, C-terminal domain containing protein.	19	12	5	10	59	10	11.9941123	26.153147	2.180498754
Os05g0545300|AK109	Protein kinase domain containing protein.	110	158	111	260	312	255	126.636133	275.9068	2.178736769
Os09g0493000|AK107	Conserved hypothetical protein.	173	270	210	656	370	396	217.604733	474.0886	2.178668601
Os04g0452100|AK072	Cryptochrome 1 apoprotein (Blue light photoreceptor).	8	6	5	20	12	10	6.36498633	13.8618423	2.177827509
Os01g0948900|AK100	Ankyrin repeat containing protein.	117	96	80	234	196	209	97.7858967	212.909333	2.177301028
Os09g0550500|AK109	Conserved hypothetical protein.	9	8	6	7	33	12	7.888768	17.1754437	2.177202279
Os03g0108300|AK061	Cellulase (EC 3.2.1.4).	23	37	20	112	23	39	26.6460767	57.9995233	2.176662781
osa-miR395r|Os04|NA|	miRNA	24	16	7	23	70	10	15.7539023	34.290565	2.17663943
Os05g0268500|AK107	Serine carboxypeptidase II-2 precursor (EC 3.4.16.6) (CP-MII.2) (Fragr	119	87	60	75	446	59	88.79327	193.251823	2.176424219
Os07g0650600|AK067	BLE2 protein.	342	383	488	965	649	1027	404.521667	880.3657	2.176312847
POsControl0015|genon	NONE	28	17	12	18	61	46	19.1516433	41.6780633	2.176213425
Os11g0655100|AK063	Hypothetical protein.	8	6	6	6	27	10	6.64912633	14.461089	2.174885583
POsControl0045|art|NA	NONE	15	7	9	17	39	11	10.3838097	22.5825567	2.174785304
Os10g0552200|CI2409	(No Hit)	50	35	42	26	126	124	42.2633967	91.89078	2.174240294
POsControl0030|rando	NONE	29	17	9	18	91	12	18.5424587	40.3081233	2.173828404
Os08g0250200|Os08g	ATP synthase epsilon chain, mitochondrial (EC 3.6.3.14).	75	66	50	116	182	119	63.9938067	139.035867	2.172645665
Os01g0137200|Os01g	Receptor serine/threonine kinase PR5K.	31	29	28	36	145	10	29.18308	63.385029	2.171978729
Os05g0435300|Os05g	Protein of unknown function DUF1218 family protein.	20	32	126	145	114	127	59.3606367	128.862733	2.170844866
Os01g0517500|AK069	Polygalacturonase (Fragment).	18	19	17	7	101	10	18.14086	39.3802163	2.170802064
Os06g0727900|Os06g	Conserved hypothetical protein.	20	34	29	84	50	47	27.7886767	60.3104667	2.170325251
PGmControl0003|AF03	NONE	22	8	6	11	50	18	12.0465827	26.1343167	2.169438204
Os01g0227700|CI4665	Cytochrome P450 family protein.	8	19	5	12	43	14	10.649184	23.0993933	2.169123318
Os10g0141400|Os10g	26S proteasome non-ATPase regulatory subunit 4 (26S proteasome re	22	8	13	39	45	11	14.5078223	31.4643033	2.168781958
Os01g0711900|AK108	Conserved hypothetical protein.	9	7	6	8	13	27	7.49044833	16.2439243	2.168618434
Os01g0952900|AK069	Conserved hypothetical protein.	136	118	122	243	293	279	125.307	271.699	2.168266737
Os11g0644700|AK119	Plant disease resistance response protein family protein.	9	20	7	6	62	10	11.9572813	25.924371	2.168082382
Os09g0562400|Os09g	SWIM Zn-finger domain containing protein.	33	21	9	31	91	14	20.966385	45.4566367	2.16807221
Os03g0201500|CI5623	(No Hit)	67	114	111	259	139	236	97.50978	211.329033	2.167259872
Os01g0126000|AK109	Non-protein coding transcript, uncharacterized transcript.	16	7	15	7	28	48	12.6697977	27.4427253	2.165995547
Os06g0493100|AY256	Hypothetical protein.	1318	2648	1829	3587	4697	4267	1931.64033	4183.65833	2.165857826
Os09g0566700|AK072	Hypothetical protein.	14	7	6	9	39	11	9.12590867	19.764799	2.165789701
Os10g0136400|Os10g	Serine/threonine kinase.	90	107	94	208	214	210	97.2792433	210.555667	2.164445975
Os07g0160100|AK120	YABBY2.	14	11	5	16	41	10	10.341725	22.3835233	2.164389725
Os08g0173600|AK062	Hypothetical protein.	72	58	47	106	179	99	59.1824333	128.08686	2.164271605
osa-miR440|Os11|NA|	miRNA	21	18	12	13	88	10	17.1217467	37.0548167	2.164196059
Os03g0702000|AK121	UDP-glucuronosyl/UDP-glucosyltransferase family protein.	724	828	870	1818	1666	1758	807.443467	1747.437	2.164160182

Os08g0161800|AK059	Cholinephosphate cytidylyltransferase.	26	13	10	17	69	21	16.429275	35.5476133	2.163675106
Os02g0249600|CI1572	Glutelin type-B 2 precursor.	14	8	5	9	42	10	9.24639033	20.003439	2.16337817
Os03g0790500|AK107	Esterase/lipase/thioesterase domain containing protein.	163	148	129	259	407	288	146.9882	317.970933	2.163241222
Os01g0728000|AK106	Hypothetical protein.	8	6	6	8	26	10	6.80187167	14.7140427	2.163234384
Os07g0204900|AK065	Zeta-carotene desaturase (Fragment).	1171	1367	1502	3268	2074	3399	1346.76367	2913.343	2.163217699
Os11g0579900|AK102	ARM repeat fold domain containing protein.	8	6	5	13	12	17	6.402326	13.8446667	2.162443254
Os02g0727100|AK069	Amino acid/polyamine transporter II family protein.	11	8	5	6	36	9	7.909592	17.1027277	2.162276849
Os04g0510600|AK107	TPR-like domain containing protein.	22	20	9	7	35	68	17.028013	36.7984473	2.161053514
Os06g0119900|AK067	Hypothetical protein.	11	10	5	6	40	9	8.46541167	18.293821	2.161007842
Os02g0564300|CI4964	Conserved hypothetical protein.	22	24	20	53	47	42	21.8499133	47.2173833	2.160987214
Os11g0650700|CI2345	(No Hit)	96	90	90	173	223	199	91.7466767	198.194867	2.160240282
Os03g0828600|CI1438	(No Hit)	10	9	11	8	43	13	9.83988367	21.2552633	2.160113275
Os04g0212100|Os04g	Conserved hypothetical protein.	59	53	57	73	92	200	56.32843	121.646493	2.159593181
PAtControl0001|U4334	NONE	30	22	10	8	115	10	20.55574	44.386873	2.159342013
Os05g0176800|AC144	HCO3-transporter domain containing protein.	32	21	22	24	127	11	25.12484	54.20237	2.157321997
Os09g0499400|AK102	Hypothetical protein.	17	9	5	25	35	10	10.70215	23.0832237	2.156877232
Os10g0559500|AK072	2OG-Fe(II) oxygenase domain containing protein.	569	685	632	1480	1251	1335	628.4513	1355.47167	2.156844399
Os03g0405500|AK104	PDI-like protein.	5605	6253	5727	13383	11217	13325	5861.80467	12641.5467	2.15659637
Os06g0583900|AK072	Pectate lyase homolog (EC 4.2.2.2).	18	10	5	12	49	9	10.8479357	23.378691	2.155128102
Os08g0140700|Os08g	Conserved hypothetical protein.	10	9	7	7	34	13	8.378669	18.0536623	2.154717215
Os03g0326200|AK099	Phospholipid-transporting ATPase 1 (EC 3.6.3.1) (Aminophospholipid f	137	164	147	349	325	291	149.355067	321.7853	2.15449872
Os08g0193600|CI1441	Cyclin-like F-box domain containing protein.	15	7	6	9	29	22	9.18545833	19.7857987	2.154034992
osa-miR395c|Os04|NA	miRNA	15	7	111	7	269	11	44.412003	95.615867	2.1529285
Os02g0812600|Os02g	Cyclin-like F-box domain containing protein.	30	38	35	160	53	10	34.6217833	74.5117633	2.15216422
Os12g0428300|Os12g	Retrotransposon gag protein family protein.	12	15	16	17	21	54	14.3175167	30.81069	2.151957684
Os06g0288100|AK068	Receptor-like protein kinase.	2709	2805	2704	5378	6188	6115	2739.36633	5893.777	2.151511073
Os01g0911700|AK105	Transcription activator VP1-rice.	8	9	7	6	37	10	8.26931	17.790564	2.151396428
Os04g0469300|CI4414	(No Hit)	67	76	73	192	162	111	72.03223	154.964367	2.151319856
Os07g0601900|AK105	NADPH HC toxin reductase (Fragment).	47	19	27	16	94	91	31.05439	66.77767	2.150345571
Os12g0189500|Os12g	Flavin-containing monooxygenase FMO family protein.	8	6	17	11	46	10	10.4311197	22.4304167	2.150336434
Os08g0163800|AK107	Protein of unknown function DUF260 domain containing protein.	8	9	6	12	25	10	7.46270067	16.0471567	2.150314931
Os03g0349800|CB658	Hypothetical protein.	25	18	18	17	102	12	20.27257	43.5877233	2.15008375
Os02g0247600|CI5558	(No Hit)	8	7	6	6	28	10	6.89289933	14.8185243	2.149824568
Os05g0406200|Os05g	Inosine/uridine-preferring nucleoside hydrolase domain containing prot	39	34	22	24	158	21	31.3943633	67.49223	2.149819994
Os04g0664900|CI5509	Cell wall invertase (EC 3.2.1.26).	1393	1902	1837	3641	3484	3908	1710.69133	3677.626	2.149789344
Os05g0217800|AK106	BURP domain containing protein.	9	7	6	13	22	11	7.11374633	15.2912233	2.149531712
Os12g0429800|Os12g	Hypothetical protein.	9	7	6	7	30	11	7.43976167	15.9861207	2.148740965
Os03g0325000|Os03g	Pyruvate kinase family protein.	31	30	16	19	85	63	25.82266	55.4764467	2.148362975
Os09g0346500|AK104	Chlorophyll a-b binding protein 1, chloroplast precursor (LHCII type I C	286	140	116	286	467	411	180.646467	388.0832	2.14830219
osa-miR395h|Os08|NA	miRNA	26	17	11	16	88	11	17.7960067	38.2297067	2.148218271
Os02g0738300|AK108	Conserved hypothetical protein.	440	555	424	946	1002	1099	472.8906	1015.8482	2.148167462

Os06g0688700|CI1496	(No Hit)	278	370	283	631	684	685	310.3274	666.569233	2.147954816
Os03g0309400|AK1007	Pectinesterase family protein.	9	14	6	11	39	11	9.51083633	20.4198667	2.147010626
Os01g0102600|AK062(	Shikimate kinase domain containing protein.	242	265	294	640	458	622	267.051533	573.358267	2.14699485
Os02g0755500|AK0663	UDP-glucuronosyltransferase.	15	14	9	10	35	37	12.7575747	27.3837727	2.146471675
Os09g0522000|AF3009	CBF-like protein.	955	1360	1125	2733	2248	2400	1146.63047	2460.235	2.145621516
Os12g0136300|AK1069	Hypothetical protein.	34	33	33	93	61	61	33.52611	71.9274633	2.145416314
Os03g0724500|AK1213	Conserved hypothetical protein.	85	96	84	76	437	54	88.0779067	188.96375	2.145415998
Os06g0137700|AK1064	GDP-mannose 4,6 dehydratase 1 (EC 4.2.1.47) (GDP-D-mannose deh	41	32	30	107	64	49	34.16058	73.2722533	2.144935869
Os12g0411700|AK067(	ABC transporter related domain containing protein.	25	27	11	36	89	10	20.8975133	44.81353	2.144443183
Os01g0952900|AK1049	Conserved hypothetical protein.	142	123	115	270	277	268	126.674233	271.6041	2.144114812
Os02g0673300|Os02g(	Hypothetical protein.	17	14	7	12	43	26	12.5133113	26.8202033	2.143333816
Os06g0176200|AJ5067	Glycosyl transferase, family 31 protein.	11	14	12	41	28	10	12.27964	26.31893	2.143298175
Os12g0639500|CI0654	Conserved hypothetical protein.	337	423	382	854	843	750	380.6315	815.791433	2.143257805
Os01g0597800|AK102(	UDP-glucuronosyl/UDP-glucosyltransferase family protein.	427	456	365	1066	841	767	415.954067	891.3277	2.14285127
Os12g0188700|AK061(	Thioredoxin (TRX).	33	43	26	64	50	105	34.0802433	73.01746	2.142515806
Os07g0635500|AK106(	Cytochrome P450.	998	1060	1137	2342	1945	2558	1064.86533	2281.315	2.142350707
PGmControl0001|AF03	NONE	11	10	5	6	41	10	8.77872967	18.8046047	2.142064442
osa-miR172a|Os09|NA	miRNA	32	24	12	13	95	35	22.3279533	47.7942067	2.140554755
Os01g0675000|AK1007	Protein of unknown function DUF315 domain containing protein.	9	7	6	8	27	11	7.19014967	15.388036	2.140155172
Os10g0138300|CI4694	Conserved hypothetical protein.	41	28	21	22	100	70	29.87791	63.9307067	2.13973155
Os01g0216000|AK0584	Lipolytic enzyme, G-D-S-L family protein.	4279	12990	5962	20754	13893	15047	7743.43267	16564.6267	2.139183923
Os03g0619600|AK1193	Transcriptional factor B3 family protein.	69	53	57	115	173	95	59.61333	127.522203	2.139155845
Os08g0377100|CI4567	(No Hit)	7	8	9	9	16	28	8.22917167	17.594843	2.138106205
Os03g0140400|Os03g(	Cytochrome P450 family protein.	312	158	127	547	373	357	199.08	425.606267	2.137865515
Os06g0192800|AK070(	Zn-finger, RING domain containing protein.	3347	4340	3877	9315	7414	7988	3854.754	8239.05	2.137373747
Os08g0112900|AK069(	Lipolytic enzyme, G-D-S-L family protein.	13	40	29	13	146	16	27.2625567	58.2565733	2.136871242
Os07g0650600|AK0621	BLE2 protein.	2319	2703	3292	6464	4103	7199	2771.515	5922.23467	2.136822159
Os04g0618700|AK1207	Protein kinase domain containing protein.	803	903	910	2217	1316	2054	871.7197	1862.44	2.136512459
Os02g0812400|AK1194	Nucleotidyl transferase domain containing protein.	89	85	62	203	99	202	78.7099167	168.150793	2.136335553
Os02g0127100|AU172(	Conserved hypothetical protein.	19	9	14	9	69	10	13.718558	29.29514	2.135438725
Os06g0568800|Os06g(	Hypothetical protein.	26	11	10	19	71	11	15.684666	33.4855933	2.134925496
Os01g0793300|CI4314	(No Hit)	8	6	6	6	26	10	6.574497	14.0316917	2.134260867
Os06g0688800|CI4316	(No Hit)	489	603	439	992	1153	1120	510.0848	1088.23693	2.133443171
Os08g0282700|AK0718	Non-protein coding transcript, unclassifiable transcript.	13	13	5	12	33	23	10.6110703	22.63021	2.132698143
Os06g0547400|AK1219	Peroxidase P7 (EC 1.11.1.7) (TP7).	1265	1399	1134	2822	2211	3065	1265.706	2699.22667	2.132585819
Os01g0651000|CI4740	Lipolytic enzyme, G-D-S-L family protein.	10	8	7	7	31	13	7.953305	16.9557157	2.13190814
Os09g0123200|AY6545	Paraneoplastic encephalomyelitis antigen family protein.	17	18	14	15	61	29	16.3847233	34.92505	2.131561778
Os03g0561400|CI0771	RNA-directed DNA polymerase (Reverse transcriptase) domain contair	41	38	24	73	99	47	34.2174767	72.9193067	2.131054472
Os05g0137100|AK1108	ABC transporter, transmembrane region domain containing protein.	27	18	6	24	76	10	17.0852397	36.3965953	2.13029469
Os06g0293500|AK1104	Hypothetical protein.	609	718	842	1263	1437	1922	723.276167	1540.66633	2.130121805
PGmControl0002|AF03	NONE	31	22	16	22	87	38	22.9263233	48.8296933	2.129852773

Os06g0116400|AK105!	High-affinity nickel-transporter family protein.	509	560	544	1247	1031	1158	537.743467	1145.313	2.129850144
Os03g0150200|AY339:	Ethylene-responsive element binding protein.	14	9	10	7	52	11	10.9765	23.372033	2.129279187
Os06g0239200|CI2427	Conserved hypothetical protein.	1358	1794	1468	3278	3112	3445	1540.104	3278.444	2.128715983
Os01g0186900|AK105:	Hypothetical protein.	2191	3362	2912	7030	5127	5857	2821.536	6004.43367	2.128072676
Os05g0529700|AK108	Heat shock protein DnaJ family protein.	33	43	38	40	90	112	37.8112067	80.43932	2.127393625
Os11g0202600|AK102!	Transposase, IS4 domain containing protein.	8	6	6	6	12	24	6.63557267	14.1160133	2.127324052
Os04g0189400|AK071:	Gamma Purothionin family protein.	7	6	5	6	24	10	6.19449667	13.1761717	2.127077045
Os02g0615400|Os02g(	Leucine-rich repeat, plant specific containing protein.	36	36	23	34	106	61	31.57801	67.1640667	2.126925245
Os02g0128500|Os02g(	Hypothetical protein.	35	23	24	22	100	53	27.4921167	58.45416	2.126215333
Os07g0238600|Os07g(	Conserved hypothetical protein.	12	17	7	20	43	13	11.8543527	25.1993567	2.125747173
Os05g0515500|AK068!	O-methyltransferase ZRP4 (EC 2.1.1.-) (OMT).	8	6	5	6	13	23	6.58785867	14.002223	2.125458925
Os06g0116400|AK058:	High-affinity nickel-transporter family protein.	495	553	552	1253	1018	1129	533.2737	1133.41933	2.125398896
Os01g0885500|AK064	Conserved hypothetical protein.	20	16	7	19	63	10	14.44004	30.6814467	2.124748039
Os11g0129800|CI3938	Hypothetical protein.	61	46	39	61	120	129	48.6144033	103.292953	2.124739712
Os04g0446700|AK073(	Conserved hypothetical protein.	8	11	13	18	38	10	10.356232	22.00181	2.124499528
osa-miR167g|Os03|NA	miRNA	33	24	22	36	123	11	26.59844	56.50824	2.124494519
Os07g0582400|AK102(	Sorbitol transporter.	13114	14847	17644	32545	29471	34842	15201.7367	32286.0267	2.123838044
Os04g0691600|AK099:	30S ribosomal protein S17, chloroplast precursor (CS17).	909	257	888	1629	1135	1596	684.535367	1453.39167	2.123179806
Os11g0626700|AK120	Hypothetical protein.	25	19	7	24	62	22	17.0191587	36.0997433	2.121123849
Os07g0571800|AK072(	Non-protein coding transcript, uncharacterized transcript.	8	6	5	6	12	23	6.46605167	13.713301	2.120815253
osa-miR171i|Os03|NA|(	miRNA	11	15	9	30	36	10	11.8173953	25.0620983	2.120780225
Os03g0820400|AK119(	ZPT2-13.	218	411	326	799	541	685	318.338933	675.103867	2.120707824
Os01g0226600|AK121	C4-dicarboxylate transporter/malic acid transport protein family protein.	83	133	62	239	182	167	92.4005133	195.932567	2.120470543
POsControl0022|genon	NONE	8	7	5	10	22	10	6.56390233	13.9166983	2.120186686
POsControl0030|rando	NONE	24	20	10	18	80	16	18.05931	38.26805	2.119020605
Os04g0272700|AK071(	UDP-glucuronosyl/UDP-glucosyltransferase family protein.	25	13	13	27	40	40	16.6920333	35.3641233	2.118622856
Os01g0114600|AU032(	Receptor-like kinase ARK1AS (Fragment).	26	17	18	24	70	36	20.57186	43.58283	2.118565361
Os11g0181800|AK071!	Short-chain dehydrogenase Tic32.	33	27	24	58	94	26	28.0445833	59.4132233	2.118527583
Os02g0186800|Os02g(	Cytochrome P450 family protein.	144	156	166	357	295	334	155.1695	328.7248	2.118488492
Os09g0304800|Os09g(	Conserved hypothetical protein.	36	19	26	32	79	58	26.7365867	56.6364833	2.118313906
Os09g0429300|AK064!	Non-protein coding transcript, putative npRNA.	43	41	47	76	70	132	43.8232167	92.81279	2.117890859
osa-miR395i|Os08|NA|(	miRNA	32	17	14	38	83	14	21.2331233	44.9676767	2.117807916
Os10g0146300|Os10g(	Hypothetical protein.	64	52	40	57	156	119	52.2634167	110.681843	2.117768994
Os02g0692400|CI0380	(No Hit)	403	436	412	966	844	838	416.879533	882.761333	2.117545388
Os10g0567400|AK067	Rieske [2Fe-2S] region domain containing protein.	53	41	34	35	212	23	42.48801	89.96603	2.117445133
Os04g0650700|AK068(	L-asparaginase (L-asparagine amidohydrolase).	543	428	461	940	960	1134	477.454267	1010.91833	2.117309246
Os10g0113900|AK068	NADPH-dependent codeinone reductase (EC 1.1.1.247).	222	278	318	625	504	604	272.693033	577.2782	2.116952505
Os01g0864300|AK063(	Harpin-induced 1 domain containing protein.	56	48	34	106	141	44	45.8377367	97.0139833	2.116465393
Os09g0366500|AK101:	Conserved hypothetical protein.	17	11	5	22	39	10	11.203209	23.7086833	2.116240386
Os03g0800500|AK070:	Putative small multi-drug export family protein.	325	326	335	734	616	735	328.4665	695.0515	2.116049886
Os07g0516700|CI5439	(No Hit)	713	762	707	1465	1412	1738	727.341067	1538.061	2.11463517

Os10g0487600|AK061	Conserved hypothetical protein.	17	20	12	22	69	12	16.3031867	34.4710467	2.114374777
Os06g0726200|AY378	Endochitinase precursor (EC 3.2.1.14).	2877	3743	3349	5950	7285	7840	3322.74267	7024.98633	2.114213178
osa-miR439g|Os08|NA	miRNA	11	9	7	8	12	39	9.36528767	19.7978137	2.113956813
Os09g0518200|AK121	UDP-glucuronosyl/UDP-glucosyltransferase family protein.	311	476	445	1037	736	829	410.406033	867.289267	2.113246873
Os05g0457400|Os05g	Ribosomal protein L40e family protein.	59	46	26	39	219	21	44.0135433	92.9950067	2.11287253
Os07g0577600|AK119	Lhca2 protein.	27	28	12	59	61	19	21.9920967	46.4634667	2.112734742
Os10g0408700|AK106	Protein phosphatase 2C-like domain containing protein.	63	97	83	133	242	140	81.2147567	171.4488	2.111054777
POsControl0008|genon	NONE	7	6	5	6	22	9	5.91793533	12.4906897	2.110649908
Os11g0208900|AK111	Leucine rich repeat containing protein kinase.	11	16	10	7	52	20	12.4830267	26.3406157	2.110114507
Os01g0346900|CI5273	Histone-fold domain containing protein.	32	15	17	24	95	14	21.0198167	44.35253	2.110034103
Os08g0200300|AK121	Photosystem II 10 kDa polypeptide, chloroplast precursor.	8	6	5	20	12	10	6.594526	13.9117167	2.109585536
Os02g0579800|AK059	Fw2.2.	432	508	587	1097	818	1306	509.121867	1073.6537	2.108834388
Os09g0470000|AK059	Plastidic general dicarboxylate transporter.	233	158	178	361	414	424	189.601133	399.8363	2.108828639
Os09g0343100|CB661	Conserved hypothetical protein.	8	12	8	16	33	10	9.403285	19.82767	2.108589711
Os05g0586900|CI4059	Protein prenyltransferase domain containing protein.	38	59	45	102	104	95	47.6494467	100.47062	2.108536972
Os05g0480800|CI2601	(No Hit)	39	31	31	32	63	118	33.84979	71.3374067	2.107469697
Os01g0876100|CI3542	(No Hit)	18	21	11	22	71	11	16.4205433	34.5936233	2.106728299
Os12g0236800|CI4130	(No Hit)	35	30	15	19	79	71	26.6055067	56.0360867	2.106183783
Os02g0211500|AK109	Non-protein coding transcript, unclassifiable transcript.	20	23	22	57	50	29	21.4593433	45.1951133	2.10608091
Os08g0178400|AK062	Non-protein coding transcript, unclassifiable transcript.	156	101	119	285	211	297	125.540333	264.387433	2.105995948
Os05g0550700|Os05g	Protein kinase domain containing protein.	386	382	460	870	742	975	409.5361	862.3485	2.105671515
Os09g0380000|AK068	AMP-dependent synthetase and ligase domain containing protein.	8	7	6	6	28	10	7.04002	14.8195637	2.105045677
Os09g0522200|AK071	DRE-binding protein 1A.	4066	5207	5315	10059	10684	9965	4862.66767	10236.0253	2.1050226
Os03g0438900|AK111	Hypothetical protein.	35	18	6	20	94	10	19.5964927	41.2479567	2.104864241
Os01g0594500|AK069	Conserved hypothetical protein.	8	6	6	6	26	10	6.797114	14.3062153	2.104748476
PGmControl0001|AF03	NONE	10	12	5	6	41	10	9.03835867	19.0233753	2.104737822
Os04g0638100|AK103	Transferase family protein.	131	165	158	360	228	366	151.3013	318.336667	2.103991616
Os02g0232900|AK068	Major intrinsic protein.	65	78	70	125	184	137	70.6418567	148.601267	2.103586651
Os04g0669600|AK110	Phospholipase/Carboxylesterase family protein.	152	192	220	452	329	404	187.868733	395.164833	2.10340926
POsControl0044|art|NA	NONE	14	6	5	7	38	10	8.665637	18.2194703	2.10249637
osa-miR438|Os06|NA|c	miRNA	8	7	6	6	27	11	6.99954367	14.7163473	2.102472395
Os08g0143600|AU173	Lipase, class 3 family protein.	46	52	52	80	111	124	50.0358933	105.177997	2.102050941
Os08g0250900|AK120	Hypothetical protein.	944	988	959	2068	1909	2098	963.4954	2025.29167	2.102025258
Os03g0100100|CI2246	(No Hit)	8	8	6	6	29	11	7.332027	15.4080803	2.101476213
Os04g0561000|CI1251	MscS Mechanosensitive ion channel family protein.	217	266	263	584	444	539	248.6073	522.388133	2.101258223
Os09g0522000|AY166	CBF-like protein.	2925	4355	3602	8522	7059	7282	3627.15433	7621.11933	2.101129049
Os09g0499400|AK101	Hypothetical protein.	11	8	9	12	36	13	9.58150167	20.12225	2.100114439
Os08g0320200|AK121	Oligopeptide transporter OPT superfamily protein.	8	7	6	6	26	11	6.867308	14.4197063	2.099761119
Os11g0553500|Os11g	Protein kinase domain containing protein.	51	45	30	41	117	108	42.2857533	88.7878133	2.099709863
Os04g0627000|AK065	Roc1.	11	6	5	25	11	10	7.268153	15.2601407	2.099589905
Os04g0650700|AK121	L-asparaginase (L-asparagine amidohydrolase).	7091	5787	6741	12432	11916	16840	6539.71167	13729.37	2.099384606

Os04g0531300|AK072(	Dihydrouridine synthase, DuS family protein.	78	68	84	81	69	334	76.7709167	161.107443	2.098547866
Os04g0599000|AK111!	Protein kinase domain containing protein.	349	484	470	1026	690	1018	434.432667	911.3506	2.097794825
Os05g0425600|AK064	Hypothetical protein.	30	25	6	29	85	12	20.0646067	42.0809	2.097270118
Os04g0392300|CI0466	(No Hit)	8	6	6	10	22	10	6.68251667	14.00715	2.096089048
Os08g0293200|AK071	Conserved hypothetical protein.	22	24	37	47	16	110	27.6054867	57.8292567	2.094846483
Os04g0303500|Os04g(	Protein kinase domain containing protein.	127	195	169	367	298	364	163.753233	342.9442	2.094274373
Os05g0456200|Os05g(	Aldo/keto reductase (Fragment).	50	69	40	100	145	88	52.89663	110.7615	2.093923564
Os03g0821400|AK069	Hypothetical protein.	21	19	14	42	58	13	17.8581733	37.39361	2.093921327
Os12g0171300|CI3782	Hypothetical protein.	215	322	403	756	488	724	313.4073	656.240067	2.0938889
Os07g0635500|AK121	Cytochrome P450.	651	728	765	1535	1185	1770	714.954333	1496.79333	2.093550963
Os11g0707000|AB110	Ribulose-bisphosphate carboxylase activase (EC 6.3.4.-) (Fragments).	9	7	6	12	14	22	7.44262267	15.5799533	2.093341827
Os09g0522200|AF494	DRE-binding protein 1A.	1877	2404	2390	4687	4896	4381	2223.94367	4654.64067	2.092966983
Os12g0168100|AK107	AP2 domain containing protein RAP2.6 (Fragment).	19	26	17	46	43	40	20.55487	43.0206033	2.092964019
Os11g0683500|AJ4913	Glycoside hydrolase, family 1 protein.	12	8	7	13	31	12	8.92637433	18.6756067	2.09218278
Os09g0503000|AK109	Conserved hypothetical protein.	38	31	16	17	124	36	28.33881	59.2892267	2.09215654
Os11g0670100|Os11g(	Endonuclease/exonuclease/phosphatase family protein.	30	40	46	94	85	65	38.7922267	81.1473767	2.091846322
Os10g0110800|AK071	Nitrate transporter (Fragment).	9	7	6	6	28	11	7.14226	14.9381887	2.091521265
Os02g0831200|AK098	Protein of unknown function DUF177 domain containing protein.	28	44	37	75	86	67	36.3873367	76.09449	2.091235495
POsControl0030|rando	NONE	17	19	9	16	68	11	15.1217073	31.6202767	2.09105202
Os10g0318200|AK105	Hypothetical protein.	10	8	6	7	32	11	7.99691467	16.719832	2.090785346
Os04g0105700|AK104	Conserved hypothetical protein.	500	559	616	1332	888	1279	558.076367	1166.69187	2.09055953
Os11g0470500|Os11g(	Protein of unknown function DUF26 domain containing protein.	15	24	7	20	62	12	15.053615	31.45894	2.08979305
POsControl0010|genon	NONE	25	29	24	25	74	64	25.9734367	54.2731733	2.089564582
Os09g0522200|AF300	DRE-binding protein 1A.	4031	5310	5247	10025	10353	10098	4862.35767	10158.7	2.089253958
Os08g0163400|AK065	Sigma-70 factor family protein.	183	230	246	480	390	506	219.6599	458.673567	2.088107873
Os08g0163400|AB005	Sigma-70 factor family protein.	193	230	252	530	398	481	224.9511	469.701267	2.08801498
